# Altered brain vascularization and transcriptional changes in embryos lacking ABCA1 support a role of cholesterol in brain angiogenesis

**DOI:** 10.3389/fcell.2026.1783696

**Published:** 2026-04-22

**Authors:** Brenda Becerra, Oriana Ramírez-Herrera, Javiera Barrios, Josefina Madrid, Susan Calfunao, Fujiko Saavedra, Patricia Romo-Toledo, Jesús Juárez-Balarezo, Ignacio Casanova-Maldonado, Thomas Arnold, Verónica Palma, Dolores Busso, Nicolas Santander

**Affiliations:** 1 Instituto de Ciencias de la Salud, Universidad de O´Higgins, Rancagua, Chile; 2 Centro de Investigación e Innovación Biomédica, Universidad de los Andes, Santiago, Chile; 3 Laboratory of Stem Cells and Developmental Biology, Faculty of Sciences, Universidad de Chile, Santiago, Chile; 4 Department of Pediatrics, University of California, San Francisco, CA, United States; 5 Newborn Brain Research Institute, University of California, San Francisco, CA, United States

**Keywords:** ABCA1, angiogenesis, blood-brain barrier, brain, cholesterol

## Abstract

**Introduction:**

Lipoproteins are the main lipid carriers in extracellular fluids and mediate the exchange of hydrophobic molecules between cells and their environment in animals, including cholesterol. Lipoprotein metabolism has been implicated in the regulation of angiogenesis, suggesting that cholesterol is important for this process, but a direct role for this lipid has not been unequivocally demonstrated, particularly in the central nervous system (CNS).

**Methods:**

Here, we used genetic and pharmacological models to address this issue in different models of CNS angiogenesis.

**Results:**

We show that inactivation of ABCA1, one of the main cholesterol efflux pumps, leads to altered brain vascularization and a longer angiogenic window, while inactivation of the bidirectional cholesterol transporter SR-B1 has no effect. These functional changes are accompanied by consistent transcriptomic changes in genes involved in cholesterol synthesis and angiogenesis. In support of the role of cholesterol, experimental reduction of this lipid in cultured brain endothelial cells leads to a transcriptomic signature showing the opposite direction in those genes.

**Discussion:**

These studies support a role for ABCA1 and cholesterol in regulating a trascriptional program governing angiogenesis in the brain.

## 1 Introduction

Tissue vascularization is an absolute requirement for homeostatic function. This process occurs during development and is remarkably tissue-specific, wherein blood vessels from different organs contain endothelial cells with different properties and regulated by distinct signaling pathways (Barnett et al., 2024). Particularly, in the central nervous system (CNS) endothelial cells grow in response to specific signals (WNT/β-catenin and TGFβ (Daneman et al., 2009; Arnold et al., 2014)) in addition to classical cues, such as VEGF. In the developing brain, these signals produce sprouting from preformed vessels in a perineural plexus, which grow towards the ventricles to form a periventricular plexus in a sex-independent manner (Vasudevan and Bhide, 2008; Collignon et al., 2024). Vascular sprouting in the CNS has been extensively studied in the mouse retina; it involves specification of tip cells that extend filopodia to interact with other endothelial cells and to form new vessels in the developing plexus. Concomitantly, lagging blood vessels are stabilized by pericytes and tight junctions between endothelial cells, forming the blood-brain barrier (BBB). However, angiogenesis and BBB formation can be genetically separated: inactivation of cellular junction proteins or transporters can lead to impaired barrier with normal vessel morphogenesis, or a normal BBB with vascular malformations (Nitta et al., 2003; Santander et al., 2020).

In parallel to common signals, endothelial cell metabolism controls angiogenesis. It has been hypothesized that this effect occurs through providing appropriate anabolism and/or by the production of regulatory metabolites (Li et al., 2019). Evidence from two research groups implicates lipoprotein metabolism as a metabolic regulator of angiogenesis in zebrafish and the mouse retina (Avraham-Davidi et al., 2012; Fang et al., 2013; Mao et al., 2017). Lipoproteins are specialized structures to transport hydrophobic lipids in the bloodstream and the interstitial fluid: they contain anchor proteins that interact with cognate receptors in the cell surface, allowing uptake of lipids into the cell by endocytosis or by direct transport. Cholesterol is transported into and from the plasma membrane by these mechanisms and, therefore, at least part of the effect of lipoproteins on angiogenesis has been attributed to modulation of cellular cholesterol. In addition, lipoproteins can also induce activation of multiple signaling pathways directly by binding to their cellular receptors, especially in endothelial cells (Robert et al., 2021). The potential direct role of cholesterol in the regulation of angiogenesis in the CNS therefore remains an outstanding question. Answering this question could be relevant for the prevention of developmental vascular disorders and the management of adult cerebrovascular diseases, such as germinal matrix hemorrhage and stroke, respectively.

In this study, we have addressed this issue using genetic, biochemical, and pharmacologic interventions *in vitro* and *in vivo*. We found that lipoprotein receptors are differentially expressed during developmental brain angiogenesis in endothelial cells. Inactivation of Abca1, a cholesterol exporter and the gene mutated in Tangier disease, was associated with delayed formation of the characteristic vascular network in the brain. We defined the molecular signature associated with this vascular phenotype and found that it aligns with the molecular changes observed in cultured brain endothelial cells with experimental cholesterol reduction. These studies suggest a role of lipid metabolism in regulating key processes of CNS angiogenesis.

## 2 Methods

### 2.1 Mice

Animal procedures were conducted in accordance with the Guidelines of the AVMA and was approved by the IACUCs at Universidad de O’Higgins and Universidad de los Andes.

Mice carrying a null mutation in the Scarb1 gene (B6; 129S2-Scarb1tm1Kri/J) were maintained in a mixed C57Bl6/J × 129 background. ABCA1 KO mice in the DBA background were originally generated by Dr. Omar Francone (Homology Medicines, Inc., Bedford, MA, US) (McNeish et al., 2000) and are currently maintained at Dr. Dolores Busso’s lab. Wild-type C57Bl6/J mice for pharmacological studies were purchased from the Chilean Public Health Institute.

Mutant mice lines were maintained in the animal facility at Universidad de los Andes, while C57Bl6/J mice were housed in the animal facility at Universidad de O’Higgins under controlled light and temperature conditions with *ad libitum* food and water. Females (2–4 months old) were caged together with males (2–6 months old) and the presence of a vaginal plug was checked daily in the morning, designated that day as embryonic day 0.5 (E0.5).

To study the effect of mutations during intrauterine development, we crossed heterozygous mice to produce wild-type and mutant embryos under the same maternal conditions. Fetuses were recovered at E14.5 or E18.5. Pregnant dams were deeply anesthetized with ketamine and xylazine i.p., (0.18 mg: 0.012 mg per gram of body weight), and uterine horns were excised to dissect fetuses free of extraembryonic membranes. Fetuses were genotyped individually as described elsewhere (Quiroz et al., 2020).

Adult brains were obtained from 4-months-old male mice from each experimental group. Animals were deeply anesthetized as described above and whole brains were collected after transcardial perfusion with 10 mL PBS to remove blood.

### 2.2 Chicken chorioallantoic membranes

All procedures complied with Chilean regulations and were approved by the Institutional Animal Care and Use Committee (CICUA) of the University of Chile. We employed the chicken chorioallantoic membrane (CAM) model, with minor modifications from a previously described protocol (Casas et al., 2018). Fertilized broiler chicken eggs (Agrícola Chorombo S.A., Chile) were incubated at 38.5 °C with constant 75% humidity at the dependencies of Universidad de Chile. On embryonic day 2 (E2), 3 mL of albumin was extracted from each egg. At E4, a window was opened in the eggshell to expose the CAM. On E8, 20 µL of the assigned treatment (PBS or 20 mM methyl-β-cyclodextrin in PBS) were applied directly onto the CAM surface, which was then covered with a 12 mm glass coverslip and incubated for 4 h. At the end of this acute exposure period, the CAM region beneath the coverslip was dissected, rinsed in ice-cold PBS, and flash-frozen in liquid nitrogen. Samples were stored at −80 °C until nucleic acids extraction.

### 2.3 Cell culture

bEnd.3 cells were purchased from American Type Culture Collection (ATCC) and were maintained according to the provider´s instructions. Cells were grown in Dulbecco´s Modified Eagle Medium (DMEM) supplemented with 5% Fetal Bovine Serum (FBS), 1X GlutaMAX (Thermo), and 1% Penicillin-Streptomycin mixture (Thermo). For experiments, cells were seeded in 6-well plates and assays were performed in technical triplicates on three separate days.

On the day of the experiment, cells were washed 3 times with DMEM without FBS and then incubated for 7 h with 1 mM methyl-β-cyclodextrin (CD) in culture medium without FBS, or culture medium without FBS alone. Since CD was readily dissolved in medium, vehicle was medium alone.

For sequencing, cells were harvested directly with lysis buffer from the kit PureLink RNA Micro (Thermo). RNA was purified following manufacturer´s instructions.

### 2.4 Tissue processing

Heads and brains were fixed in 4% paraformaldehyde (PFA), washed 3 times in PBS, and cryoprotected in 30% sucrose. Heads from E14.5 fetuses were embedded whole in OCT, while E18.5 brains were dissected free from the head after cryoprotection. We obtained 20 μm coronal sections from E14.5 heads, and E18.5 and adult brains.

Vascular fragments were isolated as previously described (Santander and Arnold, 2021). Briefly, brains from E18.5 fetuses were quickly dissected and lightly disrupted in PBS. After centrifugation at 4,000 g for 20 min at 4 °C, the supernatant was discarded and the pellet was resuspended in 18% dextran 70 kDa in PBS. Samples were centrifuged at 4,000 g for 20 min at room temperature and the pelleted vascular fraction was kept at −80 °C until RNA extraction.

### 2.5 Immunofluorescence

Tissue cryosections (20 μm) were blocked and permeabilized with blocking buffer (5% normal donkey serum, 1% bovine serum albumin, 0.1% Triton X-100 in PBS) for 2 h at room temperature and immediately incubated with primary antibodies overnight at 4 °C. After 3 washes in 0.1% Tween 20 in PBS, sections were incubated with secondary antibodies (1:330) for 2 h at room temperature, washed 3 times again, and mounted with Fluoromount. Primary antibodies used and their concentrations are as follow: anti-PECAM1 1:300 (RnD AF3628), anti-CLDN5 1:200 (CST 49564S), anti-AQP4 1:300 (CST 59678S), anti-phosphoH3 1:200 (CST 9701S). Secondary antibodies raised in donkey were: anti-rabbit IgG (Thermo A-21206), anti-goat IgG (Thermo A-21432). Exogenous sulfo-NHS-biotin was detected with Streptavidin coupled to Alexa 647 (Thermo S21374).

### 2.6 Image analysis

Images were acquired using a Zeiss Axio Observer A1 inverted epifluorescence microscope. In fetal sections, we imaged and quantified one whole hemisphere section. In adult sections, we imaged four fields each from the cortex and striatum across two sections per animal. PECAM1+ blood vessels were manually traced to measure vascular area. Data are expressed as absolute area, as the total area was equal for all adult samples.

To quantify filopodia, four 40X images were randomly taken from the pallium and subpallium, along the ventricular zone where filopodia are much more abundant. Number of filopodia per vascular area (Filopodia density) was determined in all images. For E18.5, we only determined the number of isolated vessels extending filopodia, since control animals have almost none.

### 2.7 Cholesterol measurement

Cell pellets were lysed in T-Per buffer (Thermo) and extracted in 2:1 chloroform:methanol. The organic phase was allowed to evaporate and cholesterol was determined with the Amplex Red Cholesterol Assay kit (Thermo), following manufacturer´s instructions. An aliquot of the lysate was used to determine total protein content with the bicinchoninic acid method.

### 2.8 RNA sequencing

Total RNA was extracted from vascular fragments, cultured cells, or chicken CAMs with the PureLink micro RNA kit (Thermo), following manufacturer´s instructions. Libraries were constructed from SR-B1 mutant vascular fragments and cultured cells with the NEBNext Ultra II RNA Library Prep kit (New England Biolabs) and with the MGIEasy RNA Library Prep kit for vascular fragments from ABCA1 mutants and chicken CAMs. For the first set, sequencing was performed using a HiSeq4000 machine (Illumina), while the second set of samples were sequenced on a DNBSEQ-G400 (MGI). Both sequencing runs were done in 150 bp paired-end settings.

Raw reads were aligned to the mouse genome (GRCm39) or chicken genome (bGalGal1.mat.broiler.GRCg7b) using Rsubread and quantified with FeatureCounts. Differential expression analysis was performed with DESeq2, and genes were considered differentially expressed at FDR<0.1. Volcano plots were constructed with the EnhancedVolcano package. Enrichment analyses were done with PANTHER. Heatmaps show z-score and were made using the pheatmap package.

Datasets generated in this study (ABCA1, SR-B1, bEnd.3 CD cells, and chicken CAMs) were deposited in GEO GSE310310. Datasets obtained from the literature are as follows: the dataset containing brain endothelial cells during development is available in GEO GSE79306; the dataset of different brain cell-types is in GSE73721.

### 2.9 Statistical analyses

Researchers were not blinded to experimental groups. For fetuses, the number of samples corresponded to those coming from 3 different litters. Since heterozygous crosses can produce any number of wild-type and homozygous mutants, the final number was only pre-defined as at least 4, which is the necessary sample size to obtain 80% power to detect a 50% change in means with a standard deviation of 20% of the mean and at alpha = 0.05; these parameters were based on our previous studies of brain vascular morphology (Santander et al., 2020). In the case of sequencing experiments, we set the sample size to 3 for cost constraints. We used 3 samples per group from at least 2 litters for fetuses. For *in vitro* studies, we used samples from 3 different experiments (done on different days), with 3 technical replicates within each experiment.

Means of continuous data were compared with the t-test with Welch correction. Number of vessels with filopodia were compared with a generalized linear model assuming Poisson distribution and Wald´s test. Sequencing data were analyzed with DESeq2, which builds generalized linear models assuming a negative binomial distribution for each gene, followed by Wald´s test and Benjamini-Hochberg correction (FDR).

Graphs show mean and standard deviation along with individual data points. Exact p-values are reported for all comparisons. We considered p < 0.05 as statistically significant, while 0.05 < p < 0.07 was considered a trend based on size effect. Genes detected in sequencing experiments were considered differentially expressed at FDR<0.1.

## 3 Results

### 3.1 Expression of APOA1 binding partners correlates with developmental angiogenesis in the brain

**FIGURE 1:**
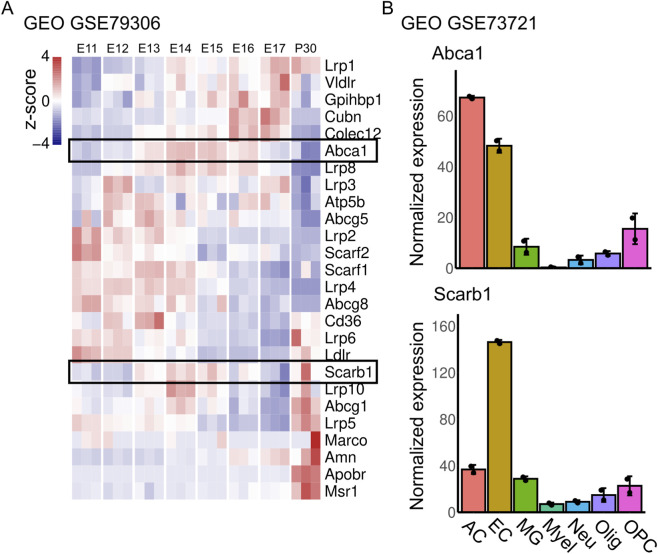
Developmental expression of lipoprotein receptors in brain endothelial cells. **(A)** Expression of all genes coding for proteins with apolipoprotein binding activity in endothelial cells from the brain during embryonic development and adulthood. **(B)** Expression of Abca1 and Scarb1 in different cell types in the adult mouse brain. AC: astrocytes, EC: endothelial cells, MG: microglia, Myel: myeloid cells, Neu: neurons, Olig: oligodendrocytes, OPC: oligodendrocyte progenitor cells.

Lipoprotein metabolism is an important regulator of angiogenesis in the zebrafish and the mouse retina by modulating cellular cholesterol (Avraham-Davidi et al., 2012; Fang et al., 2013; Mao et al., 2017), but its relevance in other angiogenic niches of the CNS has not been explored. Importantly, the role of lipoprotein receptors in brain vascularization might differ from the retina, since their developmental windows are non-overlapping, and the brain develops in a relatively hypoxic environment. To begin to address this question, we exploited published sequencing data (Hupe et al., 2017) to determine expression levels of all lipoprotein receptors in brain endothelial cells during brain vascularization in mice (Figure 1A). All receptors showed marked changes in expression along embryonic development and into adulthood. We noticed that Abca1, Lrp8, and Scarb1 were most highly expressed between E13 and E16, which is the angiogenic window in the fetal brain in mice (Figure 1A). Lrp8 (also known as APOER2) has pleiotropic effects given by its ability to modulate cell signaling, depending on the expression of each of multiple splicing variants (Passarella et al., 2022). Its role in brain development has been studied; it regulates neuron migration, at least in part by acting as a Reelin receptor. Since our focus was on the role of lipid metabolism, we did not study Lrp8 further. On the other hand, ABCA1 (coded by Abca1) and SR-B1 (coded by Scarb1) are the main APOA1 receptors and are known to mediate cholesterol transport between the plasma membrane and lipid-poor APOA1 or mature HDL, respectively (Rigotti et al., 2003; Wang and Tall, 2003). Mutations in Abca1 cause the rare genetic disorder Tangier disease (OMIM #205400) (Oram, 2000), characterized by very low HDL cholesterol, high risk of atherosclerosis, and peripheral neuropathy. Inactivation of the Abca1 in mice recapitulates all aspects of the human disease: they show extremely low plasma HDL cholesterol, and consequent multisystemic abnormalities (Christiansen-Weber et al., 2000; Timmins et al., 2005). In zebrafish, Abca1 was shown to interact genetically with Aibp to regulate angiogenesis in the trunk (Fang et al., 2013), but its role in mammalian CNS angiogenesis remains unclear, as is the case for SR-B1. Interestingly, both genes are highly expressed in endothelial cells in the adult brain, compared to most other cell types (Figure 1B; dataset from (Zhang et al., 2016)). We note that astrocytes also show high levels of Abca1 expression; however, the mouse brain lack mature asctrocytes during intrauterine development, but they play a regulatory role in brain and retinal angiogenesis after birth (Ma et al., 2012; Farhy-Tselnicker and Allen, 2018). These data suggest that these receptors might be involved in angiogenesis and/or vascular function in the brain.

### 3.2 ABCA1 deficiency is associated with vascular alterations in the brain

Tangier disease is a rare disorder of lipoprotein metabolism, wherein HDL are severely reduced leading to multiorgan dysfunction, and the development of associated pathologies, i.e., atherosclerosis (Oram, 2000). Despite the presence of peripheral neuropathies in Tangier disease, brain development has been poorly studied. Moreover, brain angiogenesis in models of the disease remain unexplored.

We explored the role of ABCA1 and SR-B1 in brain angiogenesis by analyzing brain vascularization at different developmental timepoints in control and knock-out mice. In the mouse, brain angiogenesis is maximal at E14.5 and declines rapidly, leading to vascular remodeling with almost non-existent tip cells by E18.5 (Arnold et al., 2014). In adult animals, no angiogenesis is normally observed outside of pathological conditions. We obtained fetal brains at E14.5 and E18.5, and adult brains at P120 to detect blood vessels and quantified vascular coverage and branching (see Methods).

**FIGURE 2:**
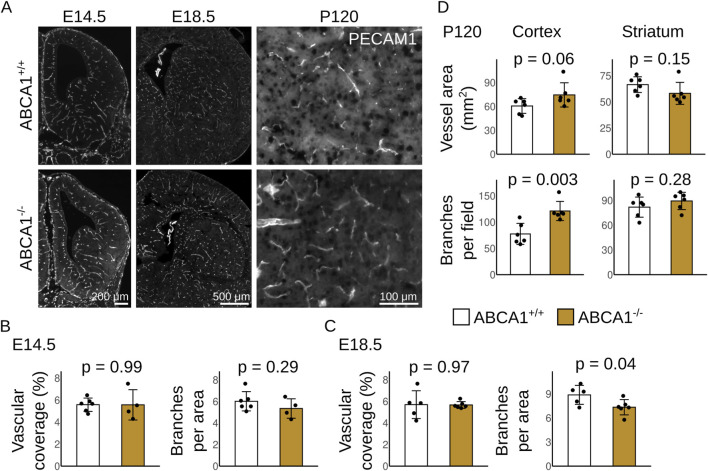
Alterations in the brain vasculature of fetuses lacking ABCA1. **(A)** Representative coronal sections of fetal brains obtained from ABCA1^+/+^ and ABCA1^−/−^ fetuses at E14.5, E18.5 and P120 and stained for PECAM1 to reveal blood vessels. Bars: 200 μm in E14.5; 500 μm in E18.5; 100 μm in P120. **(B–D)** Morphometric analyses in blood vessels at indicated developmental stages. N: E14.5 N = 6 ABCA1^+/+^ and 4 ABCA1^−/−^; E18.5 N = 6 per group; P120 N = 6 per group. Exact p-values are shown; t-test with Welch correction.

In fetuses lacking Abca1 (ABCA1^−/−^), the area of the brain covered by blood vessels was normal at both E14.5 and E18.5, compared to control wild-type fetuses (ABCA1^+/+^) (Figures 2A–C). In contrast, adult ABCA1^−/−^ mice showed a trend to elevated vascular coverage in the cortex (p = 0.06; Figure 2D). This effect was not observed in the striatum (Figure 2D). Strikingly, the brain vasculature in ABCA1^−/−^ fetuses was less branched at E18.5 compared to ABCA1^+/+^ fetuses (Figure 2C). However, this phenotype was reverted by adulthood, when ABCA1^−/−^ mice had more branches than their control counterparts (Figure 2D). This phenotypic switch suggests that ABCA1 could have different functions along intrauterine and postnatal development. It is likely that ABCA1 in astrocytes modulates angiogenic remodelling during postnatal brain expansion, consistent with previous studies (Ma et al., 2012).

**FIGURE 3:**
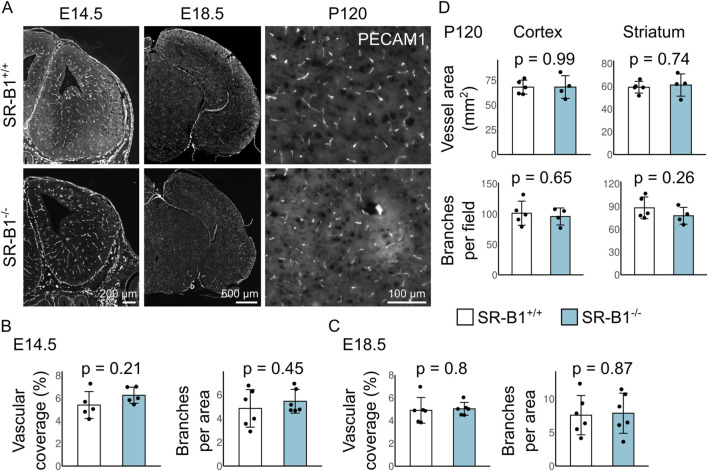
Brain vascular morphogenesis is normal in SR-B1 mutants. **(A)** Coronal sections from SR-B1^+/+^ and SR-B1^−/−^ fetal brains were stained to reveal PECAM1 at E14.5, E18.5 and P120. Bars: 200 μm in E14.5; 500 μm in E18.5; 100 μm in P120. **(B–D)** Analyses of morphometric parameters. N: E14.5 N = 5–6; E18.5 N = 6 per group; P120 N = 5 SR-B1^+/+^ and 4 SR-B1^−/−^. Differences were evaluated with t-test with Welch correction.

SR-B1, the protein product of the Scarb1 gene, is a multifunctional transporter that binds mature HDL and mediates the transfer of cholesterol and lipophilic vitamins between the lipoprotein and the membrane (Rigotti et al., 2003). It has been implicated in maintenance of BBB integrity after experimental stroke (Tran-Dinh et al., 2020) through an undescribed mechanism. We have described that a proportion of fetuses lacking SR-B1 (SR-B1^−/−^) develop exencephaly due to impaired vitamin E transport (Santander et al., 2013; 2017); we only analyzed grossly normal fetuses to avoid potential vascular malformations produced solely because of abnormal brain growth in exencephaly. Here, we observed normal vascular development in SR-B1−/− compared to controls (SR-B1^+/+^) (Figure 3A). We did not detect differences in vascular coverage or branching at E14.5, E18.5, or P120 in brains of SR-B1^−/−^ animals compared to controls (Figures 3B–D).

These studies indicate that ABCA1 -but not SR-B1- is important for developmental angiogenesis in the brain. Lack of ABCA1 produces a dynamic phenotype characterized by abnormal vascular growth in the cortex.

### 3.3 Lack of ABCA1 alters the transcriptome of brain blood vessels and changes their developmental dynamics

**FIGURE 4:**
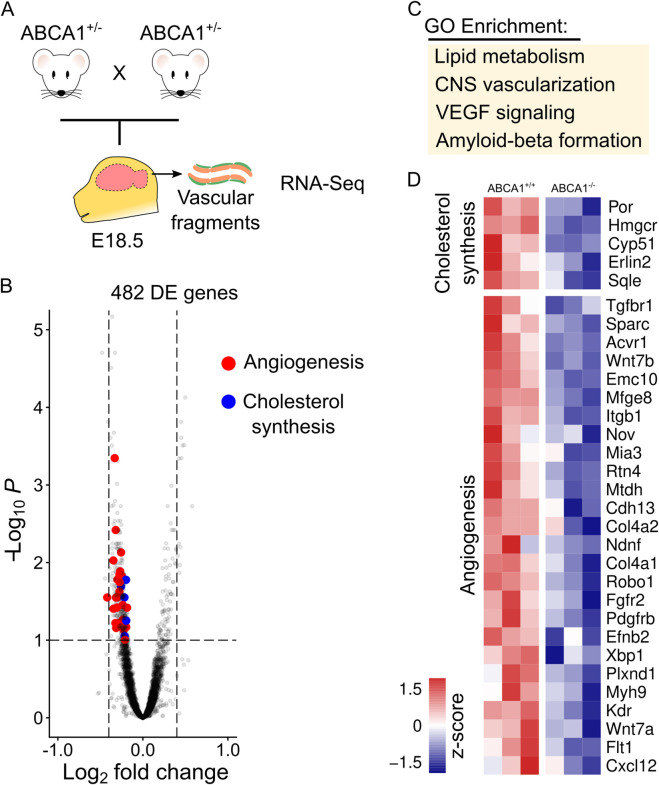
Transcriptomic changes associated with ABCA1 deficiency. **(A)** Fetal brains from heterozygous intercrosses were collected at E18.5 and vascular fragments were isolated for transcriptomic profiling. **(B)** Volcano plot showing differentially expressed genes. Genes involved in cholesterol synthesis (blue dots) or angiogenesis (red dots) are highlighted. **(C)** Enriched biological processes in the list of differentially expressed genes. **(D)** Levels of differentially expressed genes involved in cholesterol synthesis and angiogenesis.

Brain angiogenesis is controlled by the complex interaction of multiple signaling pathways and metabolic cues, which are difficult to understand in isolation. In order to understand the landscape of changes associated with Abca1 inactivation in brain blood vessels during development, we isolated vascular fragments from the brain of ABCA1^−/−^ fetuses (and their controls) at E18.5 and used RNA sequencing to define their transcriptional profiles (Figure 4A). Vascular fragments obtained by our protocol are enriched for endothelial cells and depleted of neural progenitors, but they are expected to contain some mural cells (Santander et al., 2020). Importantly, we did not detect major differences in the cellular composition of the preparations, by analyzing the expression of markers of each brain cell-type (Supplementary Table S1). In these samples, we detected 482 differentially expressed genes, most of which were downregulated in ABCA1^−/−^ brain vascular fragments (Figure 4B; Supplementary Table S2).

Enrichment analysis based on gene ontology (GO) terms revealed that gene changes associated with ABCA1 deficiency included genes related to lipid and angiogenic processes (Figure 4C). These terms correlated well with the known ABCA1 function in lipoprotein metabolism and with the vascular phenotype observed here. Since ABCA1 is a lipid pump that extrudes cholesterol from the plasma membrane to lipid-poor APOA1, we expected cholesterol to accumulate in cells from mutant animals. Consistently, we observed reduced expression of key enzymes involved in cholesterol synthesis (Hmgcr, Sqle) (Figure 4D). These enzymes are negatively regulated by cellular cholesterol via reduced activity of the master regulator SREBP2. Interestingly, we also observed downregulation of all differentially expressed genes involved in angiogenesis (Figure 4D). Of these genes, several were associated with TGFβ signaling, which has been shown previously to restrict sprouting angiogenesis (Arnold et al., 2014). Additionally, Flt1 (coding for VEGFR1) downregulation could reduce the VEGF scavenging by the soluble decoy receptor. On the other hand, strong proangiogenic genes (Kdr, Wnt7a/b) were also downregulated. We suggest that the balance of these complex gene expression changes leads to the dynamic phenotype of ABCA1^−/−^ animals: downregulation of proangiogenic molecules impairs fetal angiogenesis, while downregulation of antiangiogenic genes produces a transition to increased angiogenesis after E18.5. Notably, transcriptomic analysis of vascular fragments from SR-B1^−/−^ vascular fragments showed minimal gene expression changes, yielding only 25 differentially expressed genes compared to control samples (Figure 7A; Supplementary Table S3). This paucity of transcriptomic changes is consistent with the lack of phenotypic consequences of SR-B1 deletion in the brain vasculature and is associated with inconsistent variation in cholesterol synthesis genes (Figure 7D).

**FIGURE 5:**
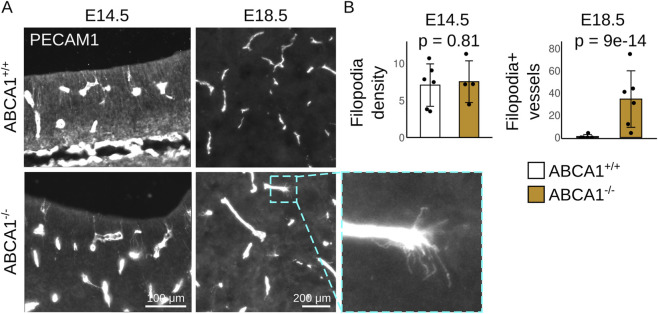
ABCA1 deficiency extends the timing of angiogenesis in the brain. **(A)** Detection of filopodia in brains from ABCA1^+/+^ and ABCA1^−/−^ fetuses at E14.5 and E18.5. A vessel with several filopodia is shown at higher magnification in the inset. Bars: 100 μm in E14.5; 200 μm in E18.5. **(B)** Quantitative analyses of filopodia. N: E14.5 N = 6 ABCA1^+/+^ and 4 ABCA1^−/−^; E18.5 N = 6 per group. Exact p-values are shown; t-test with Welch correction (E14.5) or generalized linear model with Wald´s test (E18.5).

The complexity of the transcriptomic changes led us to investigate functional consequences indicating an effect on the angiogenic state of endothelial cells in ABCA1^−/−^ brains. During sprouting angiogenesis in the brain, endothelial cells extend filopodia that interact with neighboring cells and facilitate plexi formation. We evaluated filopodia extension in endothelial cells from control and ABCA1^−/−^ brains. We analyzed pallium and subpallium separately, but we found no difference between filopodia density at the ventricular zone in these two regions; thus, we present consolidated data from both anatomical regions. At E14.5, when blood vessels extend copious amounts of filopodia in the periventricular plexus, we did not detect any difference in filopodia density in ABCA1^−/−^ brains, compared to ABCA1^+/+^ samples (Figures 5A,B). Blood vessel growth by sprouting angiogenesis decays rapidly after E14.5-E15.5, leading to vascular remodeling and lack of filopodia by E18.5 (Arnold et al., 2014). This paucity of filopodia at E18.5 was observed in brains from ABCA1^+/+^ fetuses, but ABCA1^−/−^ brains contained varying numbers of blood vessels extending several filopodia (Figures 5A,B). These vessels were found throughout the regions analyzed, both close to the ventricle and deep in the subpallium.

**FIGURE 6:**
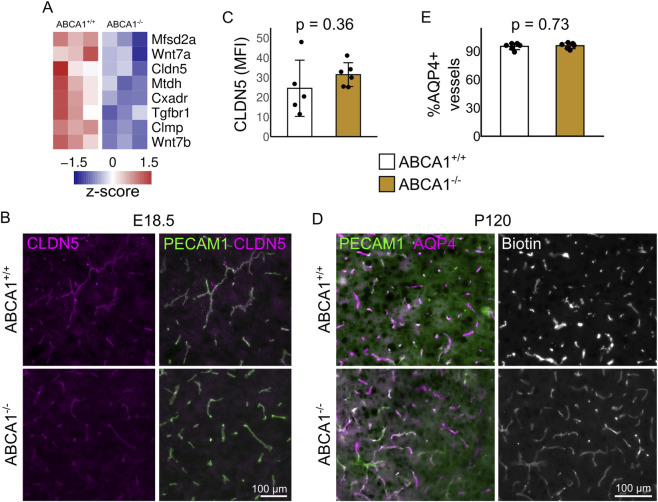
Evaluation of the blood-brain barrier in animals lacking ABCA1. **(A)** Expression levels of differentially expressed genes in vascular fragments from ABCA1^+/+^ and ABCA1^−/−^ fetal brains. **(B)** Representative images of E18.5 fetal brains stained for CLDN5 and PECAM1. Bar: 100 μm. **(C)** Quantification of fluorescence intensity for CLDN5 in E18.5 fetal brains. Groups were compared with t-test with Welch correction. N = 6 per group. **(D)** Representative stainings of adult brains to reveal AQP4, PECAM1, and intracardially infused sulfo-NHS-biotin. Bar: 100 μm. **(E)** Quantification of AQP4 positive vessels in ABCA1^+/+^ and ABCA1^−/−^ fetal brains. Groups were compared with t-test with Welch correction. N = 6 per group.

Developmental angiogenesis in the CNS is temporally coupled with blood vessel stabilization and formation of the BBB. By E18.5 the BBB is mostly functional and can exclude high molecular weight molecules (Ben-Zvi et al., 2014). This is correlated with the expression of tight junction proteins, such as CLDN5. Since brain vascular development seemed to be altered in ABCA1^−/−^ fetuses, we studied BBB formation in the brain of these animals. We observed reduced expression of a subset of genes involved in BBB formation and maintenance at E18.5 (Figure 6A), including Mfsd2a and Cldn5, an important lipid transporter at the BBB and a key member of tight junctions, respectively. Reduced expression of Cldn5 could directly lead to BBB dysfunction (Nitta et al., 2003), so we evaluated the presence of the protein *in situ*; we readily detected CLDN5 protein in blood vessels in control and mutant brains (Figure 6B), and their levels were not significantly different (Figure 6C). Furthermore, the BBB function was maintained in the brain of adult ABCA1^+/+^ and ABCA1^−/−^ mice, as shown by lack of permeability to exogenous sulfo-NHS-biotin (∼1 kDa) and normal vessel coverage by AQP4+ astrocyte endfeet (Figures 6D,E).

The combination of altered gene expression, continued production of filopodia, and morphological and functional characteristics of blood vessels in the brain of ABCA1^−/−^ fetuses, suggest that the angiogenic window is modified in these animals during development leading to an extended period of angiogenesis.

### 3.4 Cholesterol regulation of gene expression during angiogenesis in other contexts

**FIGURE 7:**
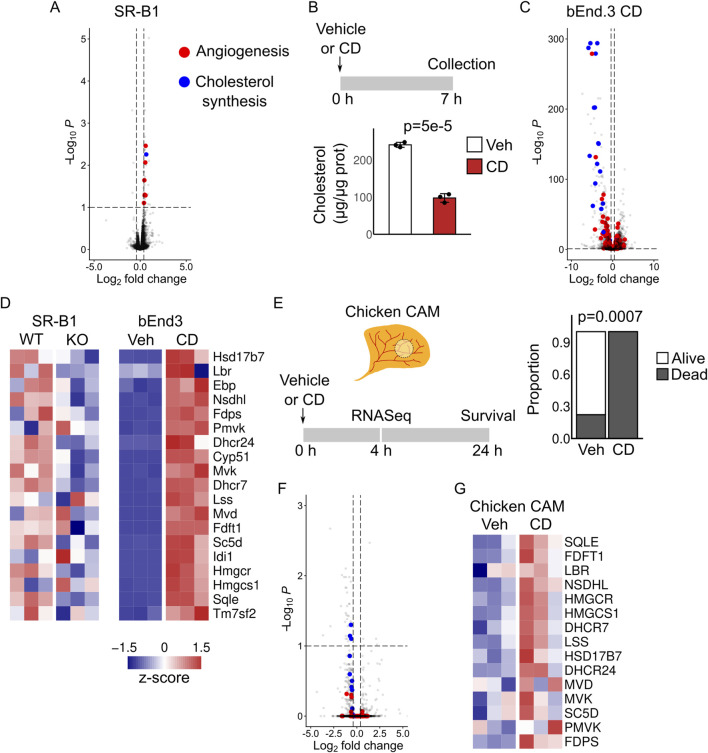
Transcriptomic effects of cholesterol reduction in bEnd3 cells and the chicken chorioallantoic membrane. **(A)** Volcano plot comparing vascular fragments from SR-B1^−/−^ and SR-B1^+/+^ fetal brains at E18.5. Genes involved in cholesterol synthesis (blue dots) or angiogenesis (red dots) are highlighted. **(B)** Bend3 cells were incubated with methyl-β-cyclodextrin (CD) or vehicle (medium alone) for 7 h and total cholesterol was measured. P-value is indicated; t-test with Welch correction. **(C)** Volcano plot showing differentially expressed genes in bEnd.3 cells exposed to CD versus vehicle. Genes involved in cholesterol synthesis (blue dots) or angiogenesis (red dots) are highlighted. **(D)** Heatmaps showing expression levels of all genes in the cholesterol synthesis pathway in mouse samples. **(E)** Chicken chorioallantoic membranes (CAM) were treated in ovo with Ringer´s solution (vehicle) or methyl-β-cyclodextrin. Survival at 24 h is shown. Exact p-value was obtained by Fisher´s exact test. N: Veh = 9; CD = 10. **(F)** Chicken CAMs treated with CD or vehicle for 4 h were used for RNA sequencing. The volcano plot shows differentially expressed genes in CAMs treated with CD. Genes involved in cholesterol synthesis (blue dots) or angiogenesis (red dots) are highlighted. **(G)** CAM expression levels of all genes in the cholesterol synthesis pathway after treatment with CD.

ABCA1 functions as a cholesterol transporter to efflux cholesterol from the plasma membrane to APOA1. As stated before, cholesterol has been indirectly implicated in angiogenesis in zebrafish and the mouse retina (Fang et al., 2013; Mao et al., 2017). In addition, statins have been used to modulate angiogenesis *in vitro* in primary human brain microvascular endothelial cells (Tan et al., 2021), though the authors attributed the observed effects to inhibition of isoprenoid production and not cholesterol synthesis. More recently, the master regulator of cholesterol synthesis, SREBP2, and HDL metabolism have been shown to safeguard lymphangiogenesis during normal development in mice and zebrafish (Kim et al., 2025; Kondo et al., 2025). These evidence raised the possibility that ABCA1 modulates angiogenesis by controlling endothelial cellular cholesterol content, though we cannot directly determine endothelial cholesterol in our preparations, due to the presence of other cell types. We next sought to identify a putative direct role of cholesterol in the functional regulation of brain endothelial cells. We used bEnd3 cells, a cell line from a mouse brain endothelioma, as a model and experimentally reduced cellular cholesterol with methyl-β-cyclodextrin (CD). CD is a molecular cage that directly removes free cholesterol from the plasma membrane. We incubated cells for a maximum of 7 h with the reagent, as longer times (24 h) proved to be evidently toxic and led to loss of cell adherence. This incubation time was sufficient to reduce cellular cholesterol content by ∼60% (Figure 7B). This reduction was associated with massive changes in transcriptional profiles of cultured cells (Figure 7C; Supplementary Table S4). These changes were characterized by increased expression of genes that synthesize cholesterol (Figure 7D) and promote angiogenesis, and reduced expression of genes restricting angiogenesis.

Finally, we used the chicken chorioallantoic membrane (CAM) as a model of non-CNS developmental angiogenesis, to evaluate if cholesterol depletion was associated with similar changes in expression outside the brain. Incubation of the CAM with CD in ovo at high concentration led to embryo death after 24 h (Figure 7E). At 4 h of CD incubation, we observed a small number of differentially expressed genes (43 genes) (Figure 7F; Supplementary Table S5). Consistent with reduced cholesterol content, there was increased expression of some cholesterol synthesis enzymes (Figure 7G); however, there was no consistent change in the expression of angiogenic genes, suggesting that the genetic interaction between these processes does not occur in the chicken CAM.

## 4 Discussion

In this work, we have shown that deficiency of Abca1 is associated with altered vascular development in the brain, extending the developmental angiogenic window and adding support for a putative role of cholesterol in this process. In addition, we used transcriptomic profiling in different models to provide molecular underpinnings of ABCA1 and cholesterol-associated regulation of brain endothelial cell function.

The role of lipoprotein metabolism in blood vessel development has been previously studied by other groups outside the brain (Avraham-Davidi et al., 2012; Fang et al., 2013; Mao et al., 2017). Seemingly conflicting results in these studies might stem from the ability of lipoproteins to activate multiple signaling pathways by directly binding to cellular receptors, by delivering protein or miR cargo, or by providing lipophilic ligands (e.g., sphingosine-1-phosphate). Indeed, recent work shows that endothelial endocytosis of HDL induced by AIBP leads to miR-223 uptake and CXCR4 regulation to restrict collateral vessel growth in the peripheral circulation (Zhu et al., 2025). Here, we provide additional evidence for a role of cholesterol in CNS angiogenesis *in vivo* using genetic models of lipoprotein receptors deficiency alongside complementary *in vitro* cell culture experiments. Our results indicate that cholesterol depletion activates pro-angiogenic programs in bEnd3 cells, consistent with reduced vascularization of the brain in ABCA1^−/−^ fetuses, where cholesterol accumulation is expected, and where a mixed expression signature was observed. We interpret this signature as a transitional stage at the end of intrauterine development from a less angiogenic phenotype towards a more angiogenic phenotype. We note, however, that the vascular phenotype in ABCA1^−/−^ is mild compared to the transcriptional changes observed. We detected reduced expression of both pro- and anti-angiogenic genes, which could lead to a relative compensation at the phenotypic level and might generate the extended angiogenic window. Alternatively, changes in the composition of vascular cells could account for some of the transcriptional alterations. We detected a minor reduction in the expression of one out of three *bona fide* pericyte marker, i.e., Pdgfrb. This could reflect a small reduction in the pericytes adhered to endothelial cells during the preparation of vascular fragments and could influence the transcriptomic profile. However, it is unlikely that this small change in pericytes would produce the large changes observed at the transcriptomic level; indeed, many of the differentially expressed genes were endothelial specific or enriched.

Previous studies using AIBP-deficient animals suggested a functional interaction between membrane cholesterol and VEGF and NOTCH signaling (Fang et al., 2013; Mao et al., 2017). We did not observe strong evidence of NOTCH activation in our models, but components of VEGF signaling were dysregulated in association with ABCA1 deficiency and cholesterol changes. Our results are, therefore, complementary to previous studies and help understand the molecular underpinnings of angiogenic regulation in relation to cholesterol metabolism.

A more direct implication of our study lies in the pathophysiology of Tangier disease, wherein no cerebrovascular alterations have been reported. We propose that altered vascular development might leave the brain susceptible to vasculopathies during the perinatal window. Although, currently, there is no evidence for a role of ABCA1 in neonatal vascular pathologies, a study showed that ABCA1 protects the BBB against overt damage after stroke in adult mice (Cui et al., 2015). Since our results show that ABCA1 is not necessary for BBB functionality in homeostasis, this evidence suggests that ABCA1 deficiency makes the BBB more susceptible to damage. Further studies should evaluate the role of ABCA1 and cholesterol in neonatal cerebrovascular events, such as germinal matrix hemorrhage and vascular malformations.

## Data Availability

The datasets presented in this study can be found in online repositories. The names of the repository/repositories and accession number(s) can be found in the article/Supplementary Material.
