# Supplementary material for: Altered brain vascularization and transcriptional changes in embryos lacking ABCA1 support a role of cholesterol in brain angiogenesis
Source: Front Cell Dev Biol. 2026 Apr 22;14:1783696. doi: 10.3389/fcell.2026.1783696 (PMC13143920; doi:10.3389/fcell.2026.1783696)
Supplement: Supplementary file 1 [file Table1.docx]

Supplementary Material

# Supplementary Tables

**Supplementary Table 1. Comparison of the expression of cellular markers in vascular preparations of ABCA1+/+ and ABCA1-/- fetal brains.**

| **Cell type** | **Gene** | **Log2FC** | **Nominal p-value** | **Adjusted p-value** |
| --- | --- | --- | --- | --- |
| Progenitors | Sox2 | 0.16 | 0.070 | 0.34 |
| Progenitors | Nes | 0.09 | 0.31 | 0.71 |
| Neuron lineage | Tbr1 | -0.08 | 0.27 | 0.67 |
| Astrocyte lineage | Sox9 | 0.02 | 0.80 | 0.96 |
| Macrophages | Cx3cr1 | 0.15 | 0.31 | NA |
| Oligodendrocytes | Pdgfra | -0.17 | 0.064 | 0.33 |
| Pericytes | Pdgfrb | -0.31 | 0.0014 | 0.04 |
| Pericytes | Anpep | -0.32 | 0.10 | NA |
| Pericytes | Acta2 | -0.28 | 0.65 | NA |

NA adjusted p-values indicate low detected counts of that gene.

**Supplementary Table 2. Differentially expressed genes in ABCA1^-/-^ fetal brains.**

| **Symbol** | **Log2FC** | **padj** |
| --- | --- | --- |
| Epha4 | -0.22 | 0.0142 |
| Acsl3 | -0.21 | 0.0642 |
| Lamc1 | -0.27 | 0.0483 |
| Slc39a10 | -0.27 | 0.0167 |
| Xpr1 | -0.20 | 0.0575 |
| Tram1 | -0.32 | 0.0280 |
| Itm2c | -0.23 | 0.0831 |
| Pgap1 | -0.22 | 0.0266 |
| Ptma | 0.17 | 0.0659 |
| Hspe1 | 0.39 | 0.0104 |
| Kcnq5 | -0.36 | 0.0095 |
| Eif4e2 | 0.22 | 0.0959 |
| Gm10222 | 0.33 | 0.0031 |
| Gm29216 | 0.22 | 0.0994 |
| Bai3 | -0.23 | 0.0329 |
| Prox1 | 0.38 | 0.0038 |
| Bmpr2 | -0.24 | 0.0173 |
| Lrrn2 | -0.28 | 0.0220 |
| Plxna2 | -0.21 | 0.0267 |
| Atp1b1 | -0.21 | 0.0906 |
| Pam | -0.20 | 0.0743 |
| Lhx9 | 0.35 | 0.0258 |
| Igfbp2 | -0.27 | 0.0162 |
| Gm5835 | 0.30 | 0.0403 |
| B3galt2 | -0.27 | 0.0462 |
| Igsf8 | -0.26 | 0.0249 |
| Cadm3 | -0.27 | 0.0102 |
| Dbi | 0.30 | 0.0142 |
| Mia3 | -0.23 | 0.0740 |
| Fam134a | -0.25 | 0.0142 |
| Chpf | -0.25 | 0.0936 |
| Tmem198 | -0.31 | 0.0482 |
| Chst10 | -0.23 | 0.0458 |
| Atp5e | 0.30 | 0.0766 |
| Surf4 | -0.25 | 0.0293 |
| Ntsr1 | -0.36 | 0.0066 |
| B3galt1 | -0.22 | 0.0740 |
| Cers6 | -0.18 | 0.0992 |
| Dynlrb1 | 0.22 | 0.0973 |
| Map1a | 0.24 | 0.0856 |
| Pdia3 | -0.35 | 0.0018 |
| Serinc3 | -0.19 | 0.0935 |
| Rims4 | 0.23 | 0.0997 |
| Pigt | -0.25 | 0.0934 |
| Swi5 | 0.26 | 0.0403 |
| Fjx1 | -0.32 | 0.0063 |
| D430041D05Rik | -0.21 | 0.0829 |
| Prnp | -0.23 | 0.0180 |
| Atp9a | -0.22 | 0.0403 |
| Gm14303 | 0.45 | 0.0002 |
| Tmx4 | -0.20 | 0.0908 |
| Snap25 | 0.21 | 0.0725 |
| Cd93 | -0.34 | 0.0387 |
| Gpr21 | -0.31 | 0.0173 |
| Acvr1 | -0.30 | 0.0281 |
| Romo1 | 0.30 | 0.0769 |
| Ext2 | -0.35 | 0.0057 |
| Rps21 | 0.45 | 0.0002 |
| Ptpra | -0.26 | 0.0057 |
| Slc27a4 | -0.21 | 0.0989 |
| Hspa5 | -0.28 | 0.0413 |
| Scg5 | -0.23 | 0.0373 |
| Tspan6 | -0.25 | 0.0423 |
| Zdhhc9 | -0.29 | 0.0102 |
| Tmem35 | -0.25 | 0.0168 |
| Hs6st2 | -0.38 | 0.0002 |
| Bex2 | 0.20 | 0.0868 |
| Ngfrap1 | 0.28 | 0.0116 |
| Syp | -0.19 | 0.0659 |
| Gm7331 | 0.58 | 0.0019 |
| Cd99l2 | -0.28 | 0.0038 |
| Gpm6b | -0.17 | 0.0523 |
| Tspan7 | -0.22 | 0.0281 |
| Efnb1 | -0.25 | 0.0322 |
| Pgrmc1 | -0.19 | 0.0659 |
| Kdm5c | 0.27 | 0.0719 |
| Aff2 | -0.26 | 0.0834 |
| Praf2 | -0.37 | 0.0150 |
| Bgn | -0.37 | 0.0886 |
| Rpl3-ps2 | 0.32 | 0.0236 |
| Gpr88 | -0.44 | 0.0160 |
| Ppm1l | -0.21 | 0.0719 |
| Fstl5 | -0.26 | 0.0403 |
| Lppr5 | -0.29 | 0.0554 |
| Glrb | -0.24 | 0.0908 |
| 4932438A13Rik | -0.24 | 0.0630 |
| Gm3788 | 0.43 | 0.0005 |
| Pcdh10 | -0.22 | 0.0329 |
| Ptgfrn | -0.25 | 0.0336 |
| Ssr2 | -0.30 | 0.0171 |
| Pag1 | -0.23 | 0.0754 |
| Tspan5 | -0.26 | 0.0071 |
| Syt11 | -0.20 | 0.0568 |
| Slc16a1 | -0.32 | 0.0351 |
| Gm7536 | 0.23 | 0.0236 |
| Nlgn1 | -0.23 | 0.0516 |
| Hs2st1 | -0.24 | 0.0689 |
| Ssr3 | -0.21 | 0.0418 |
| Sort1 | -0.25 | 0.0150 |
| Slc6a17 | -0.27 | 0.0066 |
| Chrnb2 | -0.23 | 0.0726 |
| Arhgap29 | -0.34 | 0.0218 |
| Hcn3 | -0.24 | 0.0567 |
| Mllt11 | 0.23 | 0.0420 |
| Lphn2 | -0.18 | 0.0448 |
| Tmem59 | -0.26 | 0.0154 |
| Clstn1 | -0.20 | 0.0513 |
| Sdc3 | -0.21 | 0.0279 |
| Ptprd | -0.18 | 0.0567 |
| Tmem50a | -0.26 | 0.0279 |
| Slc2a1 | -0.32 | 0.0102 |
| Ybx1 | 0.29 | 0.0019 |
| Leprot | -0.23 | 0.0724 |
| Mfsd2a | -0.32 | 0.0485 |
| 5730409E04Rik | 0.36 | 0.0179 |
| Epha7 | -0.21 | 0.0598 |
| Ugcg | -0.21 | 0.0643 |
| Rps20 | 0.45 | 0.0005 |
| Impad1 | -0.21 | 0.0282 |
| Cnr1 | -0.21 | 0.0458 |
| Igfbpl1 | -0.23 | 0.0179 |
| Tgfbr1 | -0.29 | 0.0168 |
| Tmeff1 | -0.21 | 0.0457 |
| Lppr1 | -0.29 | 0.0057 |
| Abca1 | -1.43 | 0.0000 |
| Alg2 | -0.22 | 0.0929 |
| Ddost | -0.29 | 0.0304 |
| Atp8a1 | -0.34 | 0.0002 |
| Atp2a2 | -0.25 | 0.0089 |
| Cyp51 | -0.22 | 0.0281 |
| Ociad1 | -0.19 | 0.0513 |
| Atp5k | 0.44 | 0.0063 |
| Kdr | -0.30 | 0.0166 |
| Flt1 | -0.32 | 0.0282 |
| Tmem132b | -0.21 | 0.0479 |
| Hs3st1 | -0.35 | 0.0280 |
| Svop | -0.30 | 0.0010 |
| Rps16-ps2 | 0.25 | 0.0645 |
| Rplp0 | 0.27 | 0.0057 |
| Gpr125 | -0.32 | 0.0202 |
| Sel1l3 | -0.26 | 0.0598 |
| Scarb2 | -0.23 | 0.0187 |
| Stim2 | -0.29 | 0.0458 |
| Pcdh7 | -0.25 | 0.0201 |
| Hrk | 0.38 | 0.0102 |
| Rpl6 | 0.39 | 0.0069 |
| Tmem33 | -0.23 | 0.0278 |
| Pom121 | -0.28 | 0.0586 |
| Rhbdd2 | -0.24 | 0.0664 |
| Por | -0.26 | 0.0205 |
| Fam20c | -0.23 | 0.0348 |
| Ttyh3 | -0.20 | 0.0638 |
| Tmed2 | -0.27 | 0.0124 |
| Mmp17 | -0.36 | 0.0003 |
| Vkorc1l1 | -0.21 | 0.0997 |
| Col26a1 | -0.32 | 0.0423 |
| Denr | 0.33 | 0.0253 |
| Kdelr2 | -0.28 | 0.0349 |
| Lmtk2 | -0.20 | 0.0997 |
| Sppl3 | -0.27 | 0.0250 |
| Erp29 | -0.28 | 0.0142 |
| Cacna2d1 | -0.22 | 0.0447 |
| Slco1c1 | -0.36 | 0.0077 |
| Sec61a1 | -0.25 | 0.0470 |
| Reep1 | -0.22 | 0.0150 |
| Pdia4 | -0.25 | 0.0849 |
| Wnt7a | -0.32 | 0.0601 |
| Tmsb10 | 0.33 | 0.0010 |
| Npy | -0.48 | 0.0000 |
| Tmem106b | -0.20 | 0.0766 |
| Met | -0.29 | 0.0924 |
| Kcna6 | -0.24 | 0.0643 |
| Arl6ip5 | -0.29 | 0.0133 |
| Tspan9 | -0.31 | 0.0066 |
| Chl1 | -0.21 | 0.0142 |
| Rps15-ps2 | 0.42 | 0.0057 |
| Ndufa5 | 0.29 | 0.0887 |
| Nap1l5 | 0.29 | 0.0528 |
| Itpr1 | -0.28 | 0.0528 |
| Snd1 | -0.22 | 0.0429 |
| Ndnf | -0.35 | 0.0393 |
| Ptpro | -0.37 | 0.0000 |
| Rpl32 | 0.32 | 0.0108 |
| Mest | -0.31 | 0.0130 |
| Plxnd1 | -0.28 | 0.0236 |
| Plxna4 | -0.22 | 0.0397 |
| Cxcl12 | -0.31 | 0.0696 |
| Gm15487 | -0.28 | 0.0202 |
| D630045J12Rik | -0.24 | 0.0205 |
| Calu | -0.24 | 0.0272 |
| 8430419L09Rik | -0.22 | 0.0836 |
| Rpn1 | -0.31 | 0.0058 |
| Gapdh | 0.35 | 0.0177 |
| Ephb6 | -0.26 | 0.0425 |
| Emc10 | -0.27 | 0.0129 |
| Grm5 | -0.21 | 0.0158 |
| Nr2f2 | 0.29 | 0.0569 |
| Fam174b | -0.39 | 0.0119 |
| St8sia2 | -0.19 | 0.0853 |
| Snrpd2 | 0.41 | 0.0081 |
| Ntrk3 | -0.20 | 0.0516 |
| Mfge8 | -0.26 | 0.0074 |
| Stx1b | 0.20 | 0.0743 |
| Rps9 | 0.26 | 0.0066 |
| Dkk3 | -0.32 | 0.0160 |
| Syt5 | -0.28 | 0.0173 |
| Cadm4 | -0.23 | 0.0588 |
| Fgfr2 | -0.28 | 0.0602 |
| Spcs2 | -0.26 | 0.0218 |
| Rabac1 | -0.30 | 0.0066 |
| Mki67 | -0.31 | 0.0423 |
| Gabrb3 | -0.22 | 0.0179 |
| Caly | -0.27 | 0.0205 |
| Clcn4-2 | -0.22 | 0.0163 |
| Sepw1 | 0.27 | 0.0229 |
| Slc8a2 | -0.26 | 0.0540 |
| Gde1 | -0.24 | 0.0407 |
| Cdipt | -0.28 | 0.0356 |
| Nucb1 | -0.24 | 0.0528 |
| Slc17a7 | -0.27 | 0.0258 |
| Aplp1 | -0.30 | 0.0023 |
| Cox6b1 | 0.22 | 0.0754 |
| Ttyh1 | -0.23 | 0.0508 |
| Clptm1 | -0.26 | 0.0224 |
| Pvrl2 | -0.35 | 0.0137 |
| Ptprk | -0.26 | 0.0133 |
| Hsp90b1 | -0.27 | 0.0322 |
| Gnptab | -0.21 | 0.0978 |
| Lama4 | -0.33 | 0.0479 |
| Slc16a7 | -0.33 | 0.0197 |
| Dctn2 | 0.20 | 0.0630 |
| Vezt | -0.19 | 0.0795 |
| Atp2b1 | -0.19 | 0.0659 |
| Slc35f1 | -0.29 | 0.0029 |
| Serinc1 | -0.26 | 0.0065 |
| Fabp7 | 0.23 | 0.0118 |
| Syt1 | -0.24 | 0.0192 |
| Gm5428 | 0.31 | 0.0160 |
| Rpl41 | 0.35 | 0.0089 |
| Aig1 | -0.31 | 0.0403 |
| Gm10335 | 0.30 | 0.0066 |
| Rps15 | 0.26 | 0.0094 |
| Sec63 | -0.23 | 0.0487 |
| Slc39a3 | -0.25 | 0.0350 |
| Lrp11 | -0.23 | 0.0573 |
| Bsg | -0.30 | 0.0005 |
| Cirbp | 0.23 | 0.0462 |
| Ranbp2 | -0.21 | 0.0567 |
| Cd24a | -0.19 | 0.0462 |
| Ckap4 | -0.32 | 0.0102 |
| Oaz1 | 0.24 | 0.0659 |
| Smpd3 | -0.27 | 0.0029 |
| Tecr | -0.20 | 0.0602 |
| Lamp1 | -0.22 | 0.0353 |
| Stox2 | 0.21 | 0.0315 |
| Tenm3 | -0.20 | 0.0418 |
| Agpat5 | -0.23 | 0.0630 |
| Gpm6a | -0.25 | 0.0033 |
| Glg1 | -0.19 | 0.0849 |
| Cdh13 | -0.24 | 0.0352 |
| Cpe | -0.25 | 0.0179 |
| Msmo1 | -0.22 | 0.0659 |
| Neto2 | -0.24 | 0.0150 |
| Itfg1 | -0.28 | 0.0057 |
| Lpl | -0.25 | 0.0497 |
| Agpat6 | -0.20 | 0.0630 |
| Sfrp1 | -0.28 | 0.0118 |
| Amfr | -0.23 | 0.0279 |
| Erlin2 | -0.21 | 0.0887 |
| Efnb2 | -0.20 | 0.0677 |
| Col4a1 | -0.32 | 0.0038 |
| Col4a2 | -0.24 | 0.0546 |
| Large | -0.28 | 0.0069 |
| Cdh8 | -0.21 | 0.0936 |
| Leprotl1 | -0.23 | 0.0750 |
| Saraf | -0.24 | 0.0257 |
| Slc7a5 | -0.26 | 0.0645 |
| Pomk | -0.31 | 0.0315 |
| Itgb1 | -0.25 | 0.0150 |
| Calr | -0.33 | 0.0029 |
| Rps13-ps1 | 0.23 | 0.0978 |
| Tm2d2 | -0.24 | 0.0898 |
| Ncan | -0.18 | 0.0989 |
| Has3 | -0.32 | 0.0257 |
| Tnfrsf19 | -0.23 | 0.0887 |
| Ctsb | -0.20 | 0.0613 |
| Cacna2d3 | -0.27 | 0.0408 |
| Dnajc3 | -0.30 | 0.0279 |
| Extl3 | -0.21 | 0.0989 |
| Clu | -0.25 | 0.0257 |
| Tkt | -0.16 | 0.0997 |
| Tm9sf2 | -0.24 | 0.0924 |
| Nefm | 0.33 | 0.0403 |
| Ktn1 | -0.25 | 0.0142 |
| Nid2 | -0.32 | 0.0338 |
| Tpt1 | 0.50 | 0.0001 |
| Gm10132 | 0.19 | 0.0814 |
| Mrpl52 | 0.33 | 0.0836 |
| Bmpr1a | -0.23 | 0.0546 |
| Tspan14 | -0.28 | 0.0191 |
| Slc4a7 | -0.23 | 0.0868 |
| Rps24 | 0.34 | 0.0038 |
| Lrp10 | -0.29 | 0.0354 |
| Itm2b | -0.20 | 0.0383 |
| Aplp2 | -0.20 | 0.0682 |
| Ncam1 | -0.20 | 0.0499 |
| Gm10698 | -0.33 | 0.0115 |
| Tmem30a | -0.23 | 0.0136 |
| Srpr | -0.26 | 0.0969 |
| Manf | -0.35 | 0.0481 |
| Cdon | -0.30 | 0.0383 |
| Rcn2 | -0.23 | 0.0483 |
| Tspan3 | -0.25 | 0.0093 |
| Scn3b | -0.21 | 0.0189 |
| Sorl1 | -0.23 | 0.0989 |
| Sema7a | -0.31 | 0.0057 |
| Gm5620 | 0.33 | 0.0254 |
| Fat3 | -0.32 | 0.0129 |
| Islr2 | -0.21 | 0.0675 |
| Paqr9 | -0.22 | 0.0997 |
| Olfm2 | -0.26 | 0.0224 |
| H2afx | -0.30 | 0.0214 |
| Clstn2 | -0.24 | 0.0528 |
| Rplp1 | 0.36 | 0.0038 |
| Tmem108 | -0.34 | 0.0249 |
| Sidt2 | -0.23 | 0.0756 |
| Cspg5 | -0.22 | 0.0275 |
| Dpy19l1 | -0.31 | 0.0116 |
| Stt3b | -0.25 | 0.0102 |
| Adam10 | -0.25 | 0.0208 |
| Scg3 | -0.19 | 0.0950 |
| Slc38a3 | -0.30 | 0.0484 |
| Grm2 | -0.33 | 0.0166 |
| Rpl4 | 0.19 | 0.0693 |
| Clmp | -0.31 | 0.0005 |
| Elovl5 | -0.21 | 0.0992 |
| Ube4a | -0.25 | 0.0193 |
| Elmod1 | -0.31 | 0.0038 |
| Nptn | -0.28 | 0.0038 |
| Rtn4 | -0.19 | 0.0382 |
| Igfbp4 | -0.34 | 0.0032 |
| Gm11560 | 0.39 | 0.0066 |
| Hap1 | 0.26 | 0.0536 |
| Mgat5b | -0.18 | 0.0935 |
| Gabrb2 | -0.24 | 0.0442 |
| Sez6 | -0.21 | 0.0524 |
| Rnf130 | -0.22 | 0.0254 |
| 9530068E07Rik | -0.22 | 0.0492 |
| Rpl26 | 0.47 | 0.0003 |
| Rasl10b | 0.28 | 0.0275 |
| Sparc | -0.25 | 0.0149 |
| Mfap3 | -0.22 | 0.0925 |
| Gm12254 | 0.32 | 0.0317 |
| Gdpd1 | -0.20 | 0.0665 |
| Dynll2 | 0.23 | 0.0349 |
| Wscd1 | -0.25 | 0.0647 |
| Slc35b1 | -0.25 | 0.0549 |
| Canx | -0.24 | 0.0160 |
| Timp2 | -0.21 | 0.0705 |
| Ccdc47 | -0.20 | 0.0997 |
| Fam171a2 | -0.29 | 0.0168 |
| Nsg2 | -0.17 | 0.0886 |
| Xbp1 | -0.35 | 0.0094 |
| Lpcat1 | -0.21 | 0.0479 |
| Clptm1l | -0.20 | 0.0989 |
| Gm11353 | 0.26 | 0.0417 |
| Cplx2 | 0.19 | 0.0928 |
| Arl10 | -0.23 | 0.0837 |
| Ssr1 | -0.20 | 0.0540 |
| Txndc5 | -0.27 | 0.0658 |
| Vcan | -0.24 | 0.0267 |
| Spock1 | -0.33 | 0.0016 |
| Jmy | -0.20 | 0.0901 |
| Gng4 | 0.31 | 0.0401 |
| F2r | -0.28 | 0.0696 |
| Psma2 | 0.30 | 0.0442 |
| Ntrk2 | -0.22 | 0.0267 |
| Gm9625 | 0.27 | 0.0150 |
| Hiatl1 | -0.28 | 0.0658 |
| Tbca | 0.27 | 0.0853 |
| Hmgcr | -0.20 | 0.0167 |
| Pdia6 | -0.37 | 0.0019 |
| Lrfn5 | -0.23 | 0.0373 |
| Kidins220 | -0.21 | 0.0523 |
| Flrt2 | -0.21 | 0.0353 |
| Ptprn2 | -0.25 | 0.0279 |
| Tspan13 | -0.27 | 0.0224 |
| Rtn1 | -0.22 | 0.0191 |
| Lrrn3 | -0.28 | 0.0073 |
| Nrcam | -0.21 | 0.0602 |
| Lamb1 | -0.31 | 0.0197 |
| Laptm4a | -0.22 | 0.0549 |
| Sel1l | -0.20 | 0.0702 |
| Derl1 | -0.23 | 0.0856 |
| Sqle | -0.20 | 0.0560 |
| Wnt7b | -0.28 | 0.0142 |
| Sub1 | 0.21 | 0.0750 |
| Cdh6 | -0.27 | 0.0520 |
| March6 | -0.22 | 0.0262 |
| Mtdh | -0.27 | 0.0179 |
| Nell2 | -0.33 | 0.0000 |
| Lrp12 | -0.25 | 0.0630 |
| Ext1 | -0.31 | 0.0258 |
| Nov | -0.42 | 0.0281 |
| Tmbim6 | -0.25 | 0.0179 |
| Myh9 | -0.21 | 0.0992 |
| Csdc2 | 0.19 | 0.0784 |
| Grina | -0.21 | 0.0485 |
| Sun2 | -0.21 | 0.0989 |
| Acvr1b | -0.28 | 0.0042 |
| Tmem184b | -0.26 | 0.0458 |
| Fstl1 | -0.32 | 0.0242 |
| Lsamp | -0.21 | 0.0293 |
| Ifngr2 | -0.22 | 0.0745 |
| Cd200 | -0.22 | 0.0533 |
| Cd47 | -0.24 | 0.0407 |
| Dgcr2 | -0.23 | 0.0682 |
| Rtn4r | -0.36 | 0.0168 |
| Epha3 | -0.23 | 0.0513 |
| Cadm2 | -0.25 | 0.0254 |
| Wrb | -0.21 | 0.0693 |
| Robo1 | -0.33 | 0.0005 |
| Robo2 | -0.27 | 0.0041 |
| Gm9843 | 0.25 | 0.0426 |
| Cxadr | -0.24 | 0.0094 |
| Dnajb11 | -0.26 | 0.0802 |
| Masp1 | -0.23 | 0.0712 |
| Atp13a3 | -0.20 | 0.0997 |
| Heg1 | -0.22 | 0.0887 |
| Dirc2 | -0.22 | 0.0516 |
| App | -0.21 | 0.0516 |
| Sst | -0.26 | 0.0429 |
| Cldn5 | -0.27 | 0.0997 |
| Lrfn2 | -0.36 | 0.0193 |
| Tceb2 | 0.28 | 0.0305 |
| Atp6v0c | -0.23 | 0.0408 |
| Abca3 | -0.23 | 0.0353 |
| Atf6b | -0.29 | 0.0267 |
| Man2a1 | -0.30 | 0.0717 |
| Agpat4 | -0.25 | 0.0685 |
| Spast | -0.17 | 0.0974 |
| Ergic1 | -0.24 | 0.0483 |
| Rgmb | -0.23 | 0.0391 |
| Tnfrsf21 | -0.22 | 0.0935 |
| Uqcc2 | 0.31 | 0.0728 |
| Rcan2 | 0.27 | 0.0585 |
| Yipf3 | -0.27 | 0.0686 |
| Cnpy3 | -0.29 | 0.0516 |
| H2-D1 | -0.25 | 0.0293 |
| Syngr3 | -0.23 | 0.0224 |
| Rps28 | 0.43 | 0.0135 |
| Slc8a1 | -0.26 | 0.0197 |
| Rpl10a | 0.30 | 0.0978 |
| Cdh2 | -0.24 | 0.0171 |
| Gm10269 | 0.31 | 0.0505 |
| Galnt1 | -0.22 | 0.0992 |
| Pdgfrb | -0.31 | 0.0380 |
| Fhod3 | -0.41 | 0.0002 |
| Gm7729 | 0.29 | 0.0258 |
| Ablim3 | 0.27 | 0.0167 |
| St8sia3 | -0.24 | 0.0281 |
| PCDHGB6 | -0.28 | 0.0172 |
| Ndfip1 | -0.23 | 0.0142 |
| Yipf5 | -0.25 | 0.0689 |
| Rps19-ps4 | 0.44 | 0.0108 |
| Tmed7 | -0.22 | 0.0664 |
| Syt4 | -0.36 | 0.0000 |
| Slc39a6 | -0.23 | 0.0849 |
| Camk2a | 0.25 | 0.0989 |
| Vldlr | -0.21 | 0.0516 |
| Slc1a1 | -0.22 | 0.0801 |
| Atrnl1 | -0.28 | 0.0063 |
| Pdzd8 | -0.24 | 0.0714 |
| Tm9sf3 | -0.19 | 0.0974 |
| Fth1 | 0.19 | 0.0828 |
| Tmem132a | -0.26 | 0.0136 |
| Atad1 | -0.24 | 0.0284 |
| Rtn3 | -0.26 | 0.0019 |
| Tcf7l2 | 0.27 | 0.0853 |
| mt-Nd2 | 0.23 | 0.0434 |
| mt-Atp8 | 0.49 | 0.0003 |
| mt-Cytb | 0.22 | 0.0586 |

**Supplementary Table 3. Differentially expressed genes in SR-B1^-/-^ fetal brains.**

| **Symbol** | **Log2FC** | **padj** |
| --- | --- | --- |
| Col3a1 | 0.51 | 0.0021 |
| Ptges | 0.58 | 0.0787 |
| Ube2l6 | 0.94 | 0.0078 |
| Aplnr | 0.45 | 0.0228 |
| C1qtnf4 | 0.36 | 0.0349 |
| Dll4 | 0.49 | 0.0521 |
| Vstm2l | 0.43 | 0.0293 |
| Kcns1 | 0.65 | 0.0101 |
| Mmp9 | 0.57 | 0.0521 |
| Bgn | 0.49 | 0.0043 |
| Cp | 0.45 | 0.0521 |
| Hmgcs2 | 0.66 | 0.0055 |
| Fam101a | 0.84 | 0.0491 |
| Scarb1 | 1.35 | 0.0000 |
| Eln | 0.42 | 0.0143 |
| Col1a2 | 0.50 | 0.0984 |
| Bcam | 0.49 | 0.0043 |
| Cadm4 | 0.38 | 0.0143 |
| Lsr | 0.52 | 0.0189 |
| Dkkl1 | 0.88 | 0.0293 |
| Ctgf | 0.50 | 0.0503 |
| Bsg | 0.36 | 0.0619 |
| Cdh5 | 0.41 | 0.0787 |
| Rec8 | 0.94 | 0.0000 |
| Slco2a1 | 0.52 | 0.0640 |
| Pirt | 1.41 | 0.0984 |
| Unc45b | 1.04 | 0.0861 |
| Krt15 | -0.84 | 0.0452 |
| Krt14 | -0.73 | 0.0088 |
| Krt17 | -0.86 | 0.0005 |
| Ptrf | 0.51 | 0.0005 |
| Fam171a2 | 0.38 | 0.0438 |
| Ttyh2 | 0.44 | 0.0293 |
| Foxc1 | 0.55 | 0.0086 |
| Tmed9 | 0.32 | 0.0551 |
| Angptl4 | 0.59 | 0.0035 |
| Dsc3 | 0.88 | 0.0847 |
| Adra2a | 0.45 | 0.0228 |

**Supplementary Table 4. Differentially expressed genes in bEnd3 cells treated with methyl-β-cyclodextrin.**

| **Symbol** | **Log2FC** | **padj** |
| --- | --- | --- |
| Rp1 | 1.15 | 0.0667 |
| Atp6v1h | -0.33 | 0.0063 |
| Pcmtd1 | 0.36 | 0.0066 |
| Rrs1 | -0.57 | 0.0001 |
| Cops5 | -0.32 | 0.0097 |
| Prex2 | 0.98 | 0.0006 |
| Tram1 | 0.49 | 0.0001 |
| Lactb2 | -1.01 | 0.0000 |
| Tceb1 | -0.44 | 0.0076 |
| Tmem70 | -0.70 | 0.0000 |
| Ly96 | -0.61 | 0.0808 |
| Paqr8 | 0.61 | 0.0004 |
| Efhc1 | 1.16 | 0.0001 |
| Tmem14a | 1.69 | 0.0000 |
| 1110058L19Rik | 0.75 | 0.0013 |
| Lmbrd1 | -0.37 | 0.0220 |
| Ptp4a1 | -1.44 | 0.0000 |
| Lgsn | -2.94 | 0.0000 |
| Bag2 | 0.62 | 0.0039 |
| Dst | -0.59 | 0.0073 |
| Ptpn18 | 0.73 | 0.0030 |
| Plekhb2 | -0.46 | 0.0417 |
| Neurl3 | -3.20 | 0.0000 |
| Arid5a | -2.04 | 0.0000 |
| Kansl3 | -0.60 | 0.0000 |
| Cnnm4 | -1.09 | 0.0000 |
| Ankrd39 | 0.37 | 0.0703 |
| Fam178b | 1.68 | 0.0457 |
| Zap70 | 1.48 | 0.0423 |
| 2010300C02Rik | -0.58 | 0.0036 |
| Lipt1 | -0.80 | 0.0023 |
| Eif5b | -0.65 | 0.0000 |
| Aff3 | 0.99 | 0.0001 |
| Pdcl3 | -0.59 | 0.0001 |
| Tbc1d8 | 1.26 | 0.0000 |
| Il1r1 | 0.74 | 0.0000 |
| Il18rap | -0.64 | 0.0044 |
| Slc9a2 | -2.64 | 0.0000 |
| Mfsd9 | 1.42 | 0.0070 |
| Fhl2 | -1.01 | 0.0000 |
| Nck2 | 1.00 | 0.0000 |
| Tpp2 | -0.45 | 0.0007 |
| Tex30 | -0.63 | 0.0063 |
| Kdelc1 | -0.38 | 0.0097 |
| Bivm | 0.65 | 0.0001 |
| Ercc5 | 0.58 | 0.0000 |
| Wdr75 | -0.75 | 0.0000 |
| Slc39a10 | 0.47 | 0.0669 |
| Sdpr | 1.58 | 0.0000 |
| Gls | -0.53 | 0.0001 |
| Nab1 | -0.68 | 0.0004 |
| Tmem194b | 0.64 | 0.0297 |
| Hibch | 0.56 | 0.0023 |
| Pms1 | 1.31 | 0.0004 |
| Osgepl1 | 0.93 | 0.0293 |
| Stk17b | -1.21 | 0.0000 |
| Gtf3c3 | 0.35 | 0.0676 |
| Pgap1 | 1.36 | 0.0000 |
| Ankrd44 | 1.55 | 0.0000 |
| Sf3b1 | -0.25 | 0.0969 |
| Coq10b | -1.00 | 0.0000 |
| Hspd1 | -0.82 | 0.0000 |
| Hspe1 | -0.71 | 0.0001 |
| Mars2 | -0.33 | 0.0654 |
| Plcl1 | -1.20 | 0.0000 |
| 1700066M21Rik | 0.51 | 0.0757 |
| Tyw5 | -0.77 | 0.0055 |
| Aox1 | 0.50 | 0.0000 |
| Bzw1 | -0.40 | 0.0001 |
| Clk1 | -0.52 | 0.0160 |
| Cflar | -1.20 | 0.0000 |
| Trak2 | -0.61 | 0.0018 |
| Fzd7 | 0.51 | 0.0027 |
| Sumo1 | -0.36 | 0.0894 |
| Nop58 | -1.15 | 0.0000 |
| Fam117b | 0.42 | 0.0102 |
| Abi2 | 0.55 | 0.0002 |
| Raph1 | -0.77 | 0.0000 |
| Fastkd2 | -0.43 | 0.0247 |
| Ccnyl1 | -0.36 | 0.0008 |
| Fzd5 | -0.43 | 0.0423 |
| Pikfyve | -0.42 | 0.0037 |
| Kansl1l | -0.51 | 0.0020 |
| Ikzf2 | 0.56 | 0.0958 |
| Atic | -0.74 | 0.0000 |
| Fn1 | -0.60 | 0.0436 |
| Xrcc5 | -0.60 | 0.0000 |
| Rufy4 | -1.66 | 0.0674 |
| Aamp | -0.42 | 0.0059 |
| Pnkd | 1.04 | 0.0000 |
| Tmbim1 | -0.39 | 0.0004 |
| Slc11a1 | -1.96 | 0.0238 |
| Vil1 | 1.40 | 0.0000 |
| Rnf25 | -0.62 | 0.0113 |
| Stk36 | 0.74 | 0.0008 |
| Ttll4 | -0.79 | 0.0000 |
| Slc23a3 | -1.31 | 0.0000 |
| Dnajb2 | -0.72 | 0.0000 |
| Ptprn | -1.03 | 0.0000 |
| Speg | 0.83 | 0.0011 |
| Epha4 | 1.44 | 0.0000 |
| Acsl3 | -2.56 | 0.0000 |
| Scg2 | 0.56 | 0.0161 |
| Ap1s3 | 0.85 | 0.0144 |
| Wdfy1 | -0.36 | 0.0007 |
| Ccl20 | -6.47 | 0.0000 |
| Pid1 | -0.44 | 0.0777 |
| Itm2c | 1.18 | 0.0000 |
| 2810459M11Rik | 3.33 | 0.0000 |
| Armc9 | 1.16 | 0.0000 |
| Ncl | -0.61 | 0.0000 |
| Ptma | -0.42 | 0.0221 |
| Nppc | 0.99 | 0.0000 |
| Eif4e2 | -0.36 | 0.0005 |
| Ngef | -2.08 | 0.0000 |
| Inpp5d | -0.57 | 0.0021 |
| Ugt1a6b | 1.10 | 0.0300 |
| Arl4c | 0.39 | 0.0020 |
| Cops8 | -0.33 | 0.0020 |
| Col6a3 | 0.69 | 0.0969 |
| Lrrfip1 | -0.80 | 0.0000 |
| Scly | 0.45 | 0.0111 |
| Ilkap | -0.28 | 0.0436 |
| Hes6 | 0.61 | 0.0009 |
| Per2 | 0.91 | 0.0000 |
| Traf3ip1 | 0.36 | 0.0102 |
| Capn10 | -0.57 | 0.0000 |
| Kif1a | -2.98 | 0.0000 |
| Sned1 | 1.93 | 0.0000 |
| Ppp1r7 | 0.29 | 0.0385 |
| Sept2 | -0.48 | 0.0310 |
| Bok | 1.18 | 0.0001 |
| Ing5 | -0.36 | 0.0329 |
| D2hgdh | 0.91 | 0.0000 |
| St8sia4 | -0.95 | 0.0000 |
| Tnfrsf11a | 1.45 | 0.0000 |
| Tsn | 0.28 | 0.0279 |
| Nifk | -0.50 | 0.0000 |
| Inhbb | 0.51 | 0.0001 |
| Ralb | -1.07 | 0.0000 |
| Tmem185b | -0.79 | 0.0000 |
| Epb4.1l5 | -0.82 | 0.0052 |
| Ptpn4 | 0.82 | 0.0000 |
| Tmem177 | 0.48 | 0.0137 |
| Tmem37 | 0.91 | 0.0680 |
| Dbi | -1.17 | 0.0000 |
| Insig2 | 0.35 | 0.0563 |
| Ddx18 | -0.53 | 0.0001 |
| Ccnt2 | -0.32 | 0.0520 |
| Zranb3 | 1.01 | 0.0000 |
| Ubxn4 | -0.36 | 0.0007 |
| Mcm6 | 0.45 | 0.0397 |
| Zp3r | -2.93 | 0.0000 |
| Yod1 | -0.44 | 0.0395 |
| Il24 | -2.48 | 0.0000 |
| Mapkapk2 | -0.29 | 0.0595 |
| Dyrk3 | -1.05 | 0.0000 |
| Rassf5 | -1.04 | 0.0000 |
| Ikbke | -4.51 | 0.0000 |
| Fam72a | -0.65 | 0.0017 |
| Mfsd4 | 1.00 | 0.0056 |
| Lemd1 | 1.70 | 0.0790 |
| Dstyk | 1.35 | 0.0000 |
| Mdm4 | -0.30 | 0.0610 |
| Pik3c2b | 1.56 | 0.0000 |
| Ppp1r15b | -0.64 | 0.0000 |
| Atp2b4 | 1.53 | 0.0000 |
| Prelp | 2.57 | 0.0000 |
| Btg2 | 0.45 | 0.0084 |
| Tmem183a | -0.54 | 0.0005 |
| Cyb5r1 | -0.51 | 0.0000 |
| Lgr6 | 0.92 | 0.0610 |
| Arl8a | -0.87 | 0.0000 |
| Timm17a | -0.27 | 0.0559 |
| Shisa4 | -0.99 | 0.0000 |
| Nav1 | 0.66 | 0.0015 |
| Phlda3 | 0.33 | 0.0024 |
| Kif14 | 0.66 | 0.0086 |
| Nek7 | 0.57 | 0.0029 |
| Aspm | 0.71 | 0.0001 |
| Trove2 | 0.73 | 0.0003 |
| Rgs2 | 1.34 | 0.0000 |
| Pla2g4a | 0.52 | 0.0182 |
| Ptgs2 | -2.48 | 0.0000 |
| Hmcn1 | 1.31 | 0.0024 |
| Ivns1abp | -0.58 | 0.0000 |
| Swt1 | -0.51 | 0.0020 |
| 1700025G04Rik | 0.54 | 0.0051 |
| Arpc5 | -0.23 | 0.0421 |
| Lamc2 | -1.69 | 0.0000 |
| Lamc1 | -0.83 | 0.0035 |
| Rgs16 | -2.23 | 0.0000 |
| Glul | 1.18 | 0.0000 |
| Stx6 | -0.40 | 0.0002 |
| Xpr1 | 1.02 | 0.0000 |
| Cep350 | 0.47 | 0.0572 |
| Soat1 | 0.53 | 0.0013 |
| Tor3a | -0.72 | 0.0001 |
| Ralgps2 | 0.94 | 0.0000 |
| Rasal2 | -0.52 | 0.0446 |
| 4930523C07Rik | -0.65 | 0.0000 |
| Dars2 | 0.72 | 0.0000 |
| Klhl20 | 0.84 | 0.0000 |
| Prdx6 | -0.30 | 0.0219 |
| Dnm3 | 1.18 | 0.0000 |
| Vamp4 | 0.52 | 0.0080 |
| Fmo2 | 1.75 | 0.0007 |
| Gorab | -0.54 | 0.0016 |
| Kifap3 | 0.56 | 0.0040 |
| Scyl3 | -0.28 | 0.0467 |
| Sele | -3.06 | 0.0000 |
| Blzf1 | -0.39 | 0.0018 |
| Nme7 | 0.91 | 0.0000 |
| Gpr161 | 1.18 | 0.0033 |
| Mpzl1 | 0.65 | 0.0006 |
| Rcsd1 | -0.74 | 0.0697 |
| Creg1 | 0.44 | 0.0102 |
| Cd247 | 2.04 | 0.0000 |
| Pou2f1 | 0.61 | 0.0003 |
| Tmco1 | 0.64 | 0.0000 |
| Hsd17b7 | -4.22 | 0.0000 |
| Uap1 | -1.04 | 0.0000 |
| Gm7694 | 1.32 | 0.0525 |
| Dusp12 | -0.59 | 0.0224 |
| Adamts4 | -1.51 | 0.0000 |
| Ufc1 | 0.26 | 0.0868 |
| Pfdn2 | -0.51 | 0.0560 |
| F11r | -0.37 | 0.0008 |
| Vangl2 | 1.42 | 0.0000 |
| Pex19 | 0.28 | 0.0806 |
| Dcaf8 | 0.41 | 0.0002 |
| Pea15a | 0.55 | 0.0003 |
| Igsf8 | 0.33 | 0.0229 |
| Pigm | -0.25 | 0.0690 |
| Tagln2 | -1.12 | 0.0000 |
| Aim2 | -0.41 | 0.0046 |
| AI607873 | -0.98 | 0.0004 |
| Ifi204 | -1.45 | 0.0000 |
| Mndal | -0.81 | 0.0009 |
| Mnda | -1.17 | 0.0037 |
| Ifi203 | -0.43 | 0.0595 |
| Ifi202b | -2.16 | 0.0000 |
| Ifi205 | -2.25 | 0.0000 |
| Opn3 | -1.28 | 0.0199 |
| Chml | -0.71 | 0.0066 |
| Adss | -0.44 | 0.0006 |
| Hnrnpu | -0.28 | 0.0226 |
| Smyd3 | 0.97 | 0.0000 |
| Tfb2m | -0.31 | 0.0584 |
| Cnst | 0.74 | 0.0000 |
| Sccpdh | 0.30 | 0.0613 |
| Ahctf1 | -0.47 | 0.0063 |
| Acbd3 | -0.31 | 0.0956 |
| H3f3a | 0.40 | 0.0095 |
| Ephx1 | 0.44 | 0.0000 |
| Dnah14 | -1.15 | 0.0161 |
| Degs1 | -0.46 | 0.0136 |
| Capn2 | -0.19 | 0.0846 |
| Susd4 | 1.02 | 0.0558 |
| Brox | -0.36 | 0.0035 |
| Aida | -0.45 | 0.0005 |
| Taf1a | -0.63 | 0.0000 |
| Hhipl2 | -1.45 | 0.0117 |
| Dusp10 | -1.16 | 0.0000 |
| Hlx | 0.34 | 0.0563 |
| Marc2 | 0.36 | 0.0415 |
| Bpnt1 | 0.56 | 0.0344 |
| Eprs | -0.96 | 0.0000 |
| Lyplal1 | 0.85 | 0.0001 |
| Rrp15 | -0.98 | 0.0000 |
| Kcnk2 | 0.73 | 0.0002 |
| Cenpf | 0.66 | 0.0020 |
| Ptpn14 | 0.55 | 0.0850 |
| Smyd2 | -0.32 | 0.0139 |
| Vash2 | -1.77 | 0.0000 |
| Mfsd7b | -0.42 | 0.0319 |
| Tatdn3 | 0.80 | 0.0001 |
| Batf3 | -1.96 | 0.0000 |
| Atf3 | -2.47 | 0.0000 |
| Ppp2r5a | 0.36 | 0.0026 |
| Nek2 | 0.80 | 0.0009 |
| Hhat | 0.43 | 0.0984 |
| Diexf | -0.46 | 0.0013 |
| Plxna2 | -0.46 | 0.0821 |
| Cd34 | -0.51 | 0.0001 |
| Cr1l | -0.35 | 0.0072 |
| Fam171a1 | 0.30 | 0.0538 |
| Nmt2 | -0.95 | 0.0000 |
| Rpp38 | -0.98 | 0.0015 |
| Cdnf | 0.90 | 0.0229 |
| Frmd4a | -0.73 | 0.0000 |
| Prpf18 | 0.28 | 0.0614 |
| Sephs1 | 0.39 | 0.0009 |
| Phyh | 1.09 | 0.0000 |
| Ccdc3 | 1.26 | 0.0038 |
| Camk1d | -0.81 | 0.0000 |
| Dhtkd1 | 1.68 | 0.0104 |
| Echdc3 | 0.92 | 0.0052 |
| Usp6nl | 1.13 | 0.0000 |
| Celf2 | -0.76 | 0.0010 |
| Gata3 | -1.52 | 0.0000 |
| Kin | -0.53 | 0.0446 |
| Rbm17 | -0.46 | 0.0002 |
| Itga8 | 2.76 | 0.0000 |
| Pter | -0.53 | 0.0000 |
| Cubn | 0.35 | 0.0542 |
| St8sia6 | -0.54 | 0.0207 |
| Ptpla | -0.63 | 0.0067 |
| Stam | -0.40 | 0.0034 |
| Tmem236 | -1.50 | 0.0857 |
| Arl5b | -0.63 | 0.0161 |
| Skida1 | 1.84 | 0.0000 |
| Mllt10 | -0.24 | 0.0756 |
| Commd3 | 0.34 | 0.0113 |
| Pip4k2a | 0.38 | 0.0369 |
| Msrb2 | 1.26 | 0.0000 |
| Arhgap21 | -0.45 | 0.0292 |
| Gad2 | 2.28 | 0.0000 |
| Pdss1 | -0.59 | 0.0050 |
| Mastl | 0.53 | 0.0019 |
| Spopl | -0.31 | 0.0618 |
| Il1f6 | -4.51 | 0.0116 |
| Zmynd19 | -0.50 | 0.0001 |
| Tor4a | 0.63 | 0.0000 |
| Nelfb | -0.23 | 0.0662 |
| Tubb4b | -0.25 | 0.0821 |
| Ndor1 | -0.33 | 0.0334 |
| Tmem203 | 0.39 | 0.0450 |
| Tprn | 0.51 | 0.0001 |
| Dpp7 | -0.85 | 0.0001 |
| Sapcd2 | 1.23 | 0.0000 |
| Npdc1 | -0.43 | 0.0195 |
| BC029214 | -0.68 | 0.0004 |
| Clic3 | -0.93 | 0.0027 |
| Traf2 | -0.81 | 0.0000 |
| Edf1 | -0.50 | 0.0494 |
| Mamdc4 | -0.48 | 0.0374 |
| Camsap1 | -0.48 | 0.0008 |
| Ubac1 | 0.36 | 0.0037 |
| Qsox2 | -0.37 | 0.0602 |
| Gpsm1 | -1.00 | 0.0000 |
| Sdccag3 | -0.62 | 0.0033 |
| Fam69b | 1.71 | 0.0004 |
| Surf6 | -0.38 | 0.0040 |
| Med22 | 0.49 | 0.0229 |
| Rpl7a | -0.55 | 0.0193 |
| Surf2 | -0.66 | 0.0019 |
| Rexo4 | -0.37 | 0.0015 |
| Slc2a6 | -4.28 | 0.0000 |
| Adamtsl2 | 4.62 | 0.0000 |
| Sardh | 0.72 | 0.0000 |
| Vav2 | 0.84 | 0.0000 |
| Wdr5 | -0.58 | 0.0000 |
| Rxra | 1.10 | 0.0000 |
| Col5a1 | -0.60 | 0.0003 |
| 1700007K13Rik | 1.94 | 0.0003 |
| Ralgds | 0.52 | 0.0000 |
| Gtf3c5 | 0.49 | 0.0014 |
| Ddx31 | -0.49 | 0.0007 |
| Rapgef1 | -0.50 | 0.0019 |
| Trub2 | 0.33 | 0.0265 |
| Coq4 | 0.35 | 0.0820 |
| Urm1 | -0.24 | 0.0650 |
| Wdr34 | 0.39 | 0.0084 |
| Set | -0.46 | 0.0002 |
| Zdhhc12 | -0.50 | 0.0441 |
| Zer1 | 0.96 | 0.0000 |
| Tbc1d13 | 1.03 | 0.0000 |
| Dolk | 0.45 | 0.0026 |
| Nup188 | -0.68 | 0.0000 |
| Sh3glb2 | 0.92 | 0.0000 |
| Dolpp1 | 0.42 | 0.0687 |
| Crat | 0.44 | 0.0010 |
| Ier5l | -0.62 | 0.0027 |
| Asb6 | -0.44 | 0.0046 |
| Usp20 | 0.58 | 0.0002 |
| Fnbp1 | 0.37 | 0.0124 |
| Ass1 | 0.46 | 0.0067 |
| Exosc2 | -0.40 | 0.0191 |
| Aif1l | -0.87 | 0.0000 |
| 1110008P14Rik | -0.44 | 0.0264 |
| Lcn2 | -7.12 | 0.0000 |
| Slc25a25 | -1.56 | 0.0000 |
| Fam102a | 1.37 | 0.0000 |
| Dpm2 | -0.53 | 0.0391 |
| Pip5kl1 | -1.33 | 0.0181 |
| St6galnac4 | 0.39 | 0.0003 |
| St6galnac6 | 1.44 | 0.0000 |
| Ak1 | 0.90 | 0.0000 |
| Fpgs | -0.92 | 0.0000 |
| Cdk9 | -0.50 | 0.0224 |
| Sh2d3c | -0.34 | 0.0165 |
| Ttc16 | -0.71 | 0.0630 |
| Ptrh1 | -1.06 | 0.0000 |
| 1700019L03Rik | -1.72 | 0.0000 |
| Stxbp1 | -0.35 | 0.0392 |
| Fam129b | -0.36 | 0.0071 |
| Rpl12 | -1.16 | 0.0000 |
| Slc2a8 | 1.24 | 0.0000 |
| Garnl3 | -1.00 | 0.0007 |
| Zbtb43 | -0.45 | 0.0183 |
| Mvb12b | 0.59 | 0.0000 |
| Pbx3 | 0.68 | 0.0001 |
| Mapkap1 | 0.33 | 0.0684 |
| Hspa5 | -1.13 | 0.0000 |
| Cutal | 1.44 | 0.0127 |
| AI182371 | 1.27 | 0.0081 |
| Cntrl | 0.50 | 0.0003 |
| Gsn | 0.54 | 0.0000 |
| Ggta1 | 0.76 | 0.0000 |
| Ndufa8 | 0.34 | 0.0687 |
| Lhx6 | 0.93 | 0.0000 |
| Ptgs1 | 1.75 | 0.0000 |
| Rc3h2 | -0.29 | 0.0528 |
| Strbp | 0.29 | 0.0853 |
| Dennd1a | 0.61 | 0.0001 |
| Lhx2 | 0.84 | 0.0041 |
| Olfml2a | 2.08 | 0.0000 |
| Scai | 0.65 | 0.0000 |
| Orc4 | -0.37 | 0.0055 |
| Mbd5 | 0.68 | 0.0000 |
| Mmadhc | -0.33 | 0.0050 |
| Rnd3 | -1.02 | 0.0000 |
| Stam2 | -0.49 | 0.0171 |
| Ly75 | -1.31 | 0.0001 |
| Rbms1 | -0.39 | 0.0001 |
| Tank | -0.50 | 0.0005 |
| Psmd14 | -0.46 | 0.0001 |
| Galnt3 | -1.58 | 0.0000 |
| Stk39 | 0.39 | 0.0776 |
| Spc25 | 0.51 | 0.0176 |
| Dhrs9 | -0.97 | 0.0650 |
| Fastkd1 | -0.45 | 0.0200 |
| Klhl23 | 0.95 | 0.0019 |
| Sp5 | -2.47 | 0.0000 |
| Erich2 | -1.79 | 0.0001 |
| Dcaf17 | 0.31 | 0.0669 |
| Slc25a12 | 0.81 | 0.0000 |
| Pdk1 | 1.15 | 0.0000 |
| Zak | 0.33 | 0.0047 |
| Sp3 | 0.31 | 0.0356 |
| Scrn3 | 0.91 | 0.0000 |
| Wipf1 | 0.38 | 0.0107 |
| Chrna1 | 0.86 | 0.0000 |
| Chn1 | 0.41 | 0.0067 |
| Hoxd9 | 0.55 | 0.0000 |
| Hoxd8 | 0.25 | 0.0340 |
| Hnrnpa3 | -0.53 | 0.0000 |
| Nfe2l2 | -0.67 | 0.0000 |
| Ttc30b | 1.90 | 0.0000 |
| Ttc30a2 | 1.81 | 0.0500 |
| Ttc30a1 | 1.21 | 0.0000 |
| Prkra | 0.50 | 0.0005 |
| Fkbp7 | 0.79 | 0.0000 |
| Ccdc141 | -0.47 | 0.0970 |
| Cwc22 | -0.52 | 0.0116 |
| Ube2e3 | 0.47 | 0.0036 |
| Itga4 | 0.43 | 0.0773 |
| Pde1a | 0.68 | 0.0768 |
| Dusp19 | 0.90 | 0.0000 |
| Nup35 | -0.64 | 0.0509 |
| Zc3h15 | -0.64 | 0.0000 |
| Itgav | -1.73 | 0.0000 |
| Fam171b | 1.39 | 0.0000 |
| Calcrl | 1.49 | 0.0000 |
| Tfpi | 0.35 | 0.0026 |
| 2700094K13Rik | 0.47 | 0.0067 |
| Zdhhc5 | 0.42 | 0.0040 |
| Ypel4 | 0.93 | 0.0907 |
| Ube2l6 | -0.51 | 0.0310 |
| Timm10 | -0.83 | 0.0000 |
| Slc43a3 | -0.31 | 0.0032 |
| Prg2 | -1.14 | 0.0000 |
| Tnks1bp1 | -0.28 | 0.0526 |
| Aplnr | 2.92 | 0.0000 |
| 4833423E24Rik | 2.90 | 0.0000 |
| Fnbp4 | -0.50 | 0.0019 |
| Mtch2 | -0.24 | 0.0371 |
| Kbtbd4 | 0.80 | 0.0036 |
| Psmc3 | -0.28 | 0.0598 |
| Slc39a13 | -0.43 | 0.0201 |
| Madd | 0.40 | 0.0838 |
| Acp2 | 0.48 | 0.0066 |
| Ddb2 | 0.51 | 0.0987 |
| 1110051M20Rik | 0.41 | 0.0222 |
| Zfp408 | -0.79 | 0.0000 |
| Atg13 | 0.41 | 0.0335 |
| Dgkz | 0.81 | 0.0000 |
| Creb3l1 | 1.18 | 0.0947 |
| Phf21a | 0.86 | 0.0000 |
| Gyltl1b | 1.59 | 0.0715 |
| Pex16 | -0.24 | 0.0935 |
| 1700029I15Rik | -0.67 | 0.0367 |
| Mapk8ip1 | 1.02 | 0.0000 |
| Cry2 | 0.67 | 0.0043 |
| Chst1 | -0.80 | 0.0000 |
| Syt13 | 1.72 | 0.0000 |
| Prdm11 | 0.69 | 0.0002 |
| Tspan18 | 1.96 | 0.0000 |
| Ext2 | 0.33 | 0.0321 |
| Gm13889 | -2.91 | 0.0000 |
| Alkbh3 | -0.31 | 0.0617 |
| Hsd17b12 | -1.71 | 0.0000 |
| Ttc17 | -0.46 | 0.0004 |
| B230118H07Rik | 0.82 | 0.0001 |
| Fjx1 | -3.36 | 0.0000 |
| Cd44 | -1.39 | 0.0000 |
| Cat | 0.31 | 0.0164 |
| Abtb2 | -0.61 | 0.0469 |
| Nat10 | -0.58 | 0.0001 |
| Lmo2 | 1.06 | 0.0000 |
| Fbxo3 | 0.33 | 0.0072 |
| Rcn1 | -0.56 | 0.0004 |
| Elp4 | 0.49 | 0.0543 |
| Dnajc24 | 0.72 | 0.0003 |
| Ano3 | 1.13 | 0.0000 |
| Lpcat4 | -1.51 | 0.0000 |
| Nop10 | -0.41 | 0.0339 |
| Slc12a6 | 0.60 | 0.0006 |
| Katnbl1 | -0.59 | 0.0000 |
| Dph6 | 0.30 | 0.0343 |
| BC052040 | 0.63 | 0.0002 |
| Meis2 | 1.16 | 0.0000 |
| Spred1 | -0.48 | 0.0085 |
| Fam98b | -0.37 | 0.0025 |
| Thbs1 | -1.27 | 0.0000 |
| Bmf | 3.03 | 0.0000 |
| Bub1b | 0.67 | 0.0000 |
| Plcb2 | -1.14 | 0.0535 |
| Knstrn | 0.33 | 0.0089 |
| Ivd | 1.06 | 0.0000 |
| Bahd1 | -0.23 | 0.0747 |
| Chst14 | 0.34 | 0.0593 |
| Gm14137 | -1.19 | 0.0000 |
| Vps18 | -0.45 | 0.0010 |
| Dll4 | -1.29 | 0.0000 |
| Chac1 | -2.01 | 0.0000 |
| Ino80 | -0.68 | 0.0007 |
| Exd1 | 0.53 | 0.0107 |
| Oip5 | 0.82 | 0.0029 |
| Nusap1 | 0.50 | 0.0036 |
| Mapkbp1 | 0.35 | 0.0089 |
| Pla2g4b | 1.20 | 0.0072 |
| Ehd4 | 0.76 | 0.0000 |
| Pla2g4e | -4.68 | 0.0000 |
| Vps39 | 0.26 | 0.0222 |
| Ganc | 1.37 | 0.0000 |
| Stard9 | 0.90 | 0.0000 |
| Adal | 0.74 | 0.0010 |
| Trp53bp1 | 0.42 | 0.0580 |
| Map1a | -0.53 | 0.0539 |
| Ppip5k1 | 0.74 | 0.0000 |
| Hypk | -0.73 | 0.0235 |
| Mfap1a | 0.28 | 0.0870 |
| Wdr76 | 0.90 | 0.0000 |
| Eif3j1 | -0.62 | 0.0002 |
| B2m | -0.68 | 0.0000 |
| Sord | 1.21 | 0.0000 |
| Duox2 | 1.41 | 0.0000 |
| Gatm | 0.93 | 0.0007 |
| AA467197 | -0.87 | 0.0000 |
| Myef2 | -0.27 | 0.0286 |
| Fbn1 | 1.92 | 0.0000 |
| Eid1 | 0.43 | 0.0029 |
| Galk2 | 0.54 | 0.0001 |
| Fgf7 | -1.67 | 0.0002 |
| Hdc | 0.36 | 0.0974 |
| Ncaph | 0.50 | 0.0004 |
| Tmem127 | 0.24 | 0.0488 |
| Stard7 | 0.68 | 0.0000 |
| Dusp2 | 1.16 | 0.0264 |
| Mal | 1.28 | 0.0000 |
| Mall | 0.81 | 0.0075 |
| Nphp1 | 0.64 | 0.0000 |
| Bub1 | 0.55 | 0.0003 |
| Bcl2l11 | -0.44 | 0.0188 |
| Anapc1 | 0.60 | 0.0030 |
| Mertk | -1.20 | 0.0000 |
| Tmem87b | -0.36 | 0.0051 |
| Ttl | 0.49 | 0.0064 |
| Polr1b | -0.58 | 0.0002 |
| Chchd5 | 0.41 | 0.0276 |
| Slc20a1 | -1.35 | 0.0000 |
| Ckap2l | 0.62 | 0.0019 |
| Ebf4 | 0.65 | 0.0072 |
| Vps16 | -0.71 | 0.0066 |
| Fastkd5 | -1.00 | 0.0002 |
| Slc4a11 | -1.63 | 0.0000 |
| Adam33 | 3.21 | 0.0385 |
| Hspa12b | 0.51 | 0.0001 |
| Spef1 | -0.57 | 0.0134 |
| Cdc25b | 0.64 | 0.0000 |
| Ap5s1 | 0.70 | 0.0001 |
| Pcna | -0.38 | 0.0032 |
| Cds2 | 0.39 | 0.0347 |
| Crls1 | -1.07 | 0.0000 |
| Lrrn4 | 3.60 | 0.0000 |
| Bmp2 | -2.80 | 0.0000 |
| Slx4ip | 0.50 | 0.0777 |
| Jag1 | -0.41 | 0.0244 |
| Btbd3 | 0.47 | 0.0043 |
| Esf1 | -0.84 | 0.0000 |
| Macrod2 | 0.62 | 0.0113 |
| Dstn | -0.24 | 0.0924 |
| Rrbp1 | -0.49 | 0.0000 |
| Dzank1 | 2.27 | 0.0000 |
| Rbbp9 | 0.80 | 0.0000 |
| Dtd1 | 0.53 | 0.0138 |
| Rin2 | 1.29 | 0.0000 |
| Naa20 | -0.80 | 0.0000 |
| Ralgapa2 | -1.20 | 0.0000 |
| Kiz | 0.85 | 0.0000 |
| Thbd | -1.15 | 0.0000 |
| Cst3 | 0.39 | 0.0282 |
| Zfp937 | 1.03 | 0.0001 |
| 3300002I08Rik | 1.43 | 0.0284 |
| Zfp442 | 1.32 | 0.0007 |
| Pygb | 0.37 | 0.0971 |
| Abhd12 | 0.78 | 0.0000 |
| Nsfl1c | -0.72 | 0.0000 |
| Sdcbp2 | -1.16 | 0.0000 |
| Snph | 0.91 | 0.0036 |
| Srxn1 | -1.30 | 0.0000 |
| Tcf15 | 1.19 | 0.0000 |
| Csnk2a1 | -0.28 | 0.0441 |
| Trib3 | -1.77 | 0.0000 |
| Sox12 | 1.18 | 0.0048 |
| H13 | -0.28 | 0.0185 |
| Id1 | -0.71 | 0.0004 |
| Bcl2l1 | -0.54 | 0.0000 |
| Tspyl3 | 1.23 | 0.0001 |
| Pofut1 | 0.47 | 0.0408 |
| Nol4l | 0.59 | 0.0001 |
| Snta1 | 0.37 | 0.0860 |
| 1700003F12Rik | -1.00 | 0.0880 |
| E2f1 | 0.47 | 0.0015 |
| Pxmp4 | 1.07 | 0.0000 |
| Chmp4b | -0.34 | 0.0021 |
| Eif2s2 | -0.85 | 0.0000 |
| Ahcy | -0.77 | 0.0016 |
| Pigu | -0.42 | 0.0092 |
| Trp53inp2 | -1.04 | 0.0000 |
| Acss2 | -3.32 | 0.0000 |
| Edem2 | 0.28 | 0.0446 |
| Procr | -0.59 | 0.0001 |
| Mmp24 | -1.06 | 0.0213 |
| Eif6 | -0.97 | 0.0000 |
| Fam83c | -3.18 | 0.0000 |
| Cep250 | 0.27 | 0.0276 |
| Spag4 | -0.61 | 0.0826 |
| Rbm39 | -0.54 | 0.0001 |
| Dlgap4 | -0.67 | 0.0000 |
| Tgif2 | 1.22 | 0.0010 |
| 1110008F13Rik | 0.46 | 0.0492 |
| Ndrg3 | 1.00 | 0.0000 |
| Soga1 | 0.72 | 0.0730 |
| Samhd1 | 0.46 | 0.0276 |
| Manbal | -0.36 | 0.0268 |
| Src | -1.09 | 0.0000 |
| Blcap | -0.61 | 0.0000 |
| Nnat | -1.53 | 0.0000 |
| Ctnnbl1 | 1.05 | 0.0000 |
| Vstm2l | 4.40 | 0.0073 |
| Rprd1b | 0.68 | 0.0017 |
| Tgm2 | -0.53 | 0.0002 |
| Lbp | 0.94 | 0.0000 |
| Fam83d | 0.60 | 0.0010 |
| Mafb | 2.34 | 0.0000 |
| Plcg1 | 0.68 | 0.0000 |
| Zhx3 | 0.77 | 0.0000 |
| Srsf6 | -0.63 | 0.0001 |
| Ift52 | 0.40 | 0.0007 |
| Tox2 | 1.64 | 0.0402 |
| Oser1 | -0.90 | 0.0000 |
| Fitm2 | -0.60 | 0.0007 |
| Ada | 0.62 | 0.0202 |
| Pabpc1l | -0.74 | 0.0938 |
| Sdc4 | -0.95 | 0.0000 |
| Pigt | 0.29 | 0.0359 |
| Zswim1 | 0.60 | 0.0063 |
| Spata25 | 1.84 | 0.0900 |
| Neurl2 | 1.45 | 0.0004 |
| Pltp | 0.87 | 0.0000 |
| Cd40 | -0.61 | 0.0000 |
| Elmo2 | 0.77 | 0.0000 |
| Zfp334 | 0.67 | 0.0106 |
| Prex1 | 0.67 | 0.0000 |
| Trp53rk | -0.47 | 0.0185 |
| Ddx27 | -0.64 | 0.0000 |
| 1500012F01Rik | -0.44 | 0.0569 |
| B4galt5 | -0.56 | 0.0000 |
| Spata2 | -0.56 | 0.0000 |
| Cebpb | -1.27 | 0.0000 |
| Ptpn1 | -0.62 | 0.0000 |
| Nfatc2 | 1.71 | 0.0205 |
| Zfp64 | -0.67 | 0.0000 |
| Pfdn4 | -0.49 | 0.0771 |
| Fam210b | 0.41 | 0.0389 |
| Cstf1 | -0.31 | 0.0123 |
| Cass4 | -0.66 | 0.0808 |
| Fam209 | -1.68 | 0.0309 |
| Rae1 | -0.53 | 0.0003 |
| Zbp1 | -1.86 | 0.0057 |
| Pmepa1 | -0.60 | 0.0000 |
| Vapb | 0.24 | 0.0372 |
| Npepl1 | -0.31 | 0.0916 |
| Nelfcd | 0.48 | 0.0015 |
| Slmo2 | -0.31 | 0.0240 |
| Gm8923 | 0.54 | 0.0216 |
| Gm14288 | 0.46 | 0.0938 |
| Zfp931 | 1.00 | 0.0000 |
| Sycp2 | 1.23 | 0.0000 |
| Ppp1r3d | 0.98 | 0.0000 |
| Fam217b | 1.39 | 0.0000 |
| Cdh26 | 1.36 | 0.0000 |
| Osbpl2 | 0.27 | 0.0903 |
| Adrm1 | -0.43 | 0.0548 |
| Cables2 | 0.81 | 0.0000 |
| Mrgbp | -0.66 | 0.0034 |
| Ogfr | -0.26 | 0.0349 |
| Slc17a9 | -1.26 | 0.0321 |
| Col20a1 | -0.45 | 0.0573 |
| Ppdpf | 0.70 | 0.0051 |
| Srms | 0.87 | 0.0277 |
| Gmeb2 | -0.43 | 0.0026 |
| Arfrp1 | -0.51 | 0.0011 |
| Znf512b | 0.45 | 0.0128 |
| Sox18 | 1.36 | 0.0000 |
| Rgs19 | -0.38 | 0.0193 |
| Pcmtd2 | 0.57 | 0.0000 |
| Polr3k | 0.58 | 0.0001 |
| Clcn5 | -1.03 | 0.0000 |
| Usp27x | -1.27 | 0.0000 |
| Prickle3 | 0.85 | 0.0001 |
| Magix | 1.49 | 0.0005 |
| Gpkow | 0.42 | 0.0398 |
| Tfe3 | -0.66 | 0.0000 |
| Gripap1 | -0.39 | 0.0198 |
| Otud5 | -0.60 | 0.0002 |
| Pim2 | -0.71 | 0.0012 |
| Rbm3 | -0.59 | 0.0002 |
| Tbc1d25 | -0.80 | 0.0000 |
| Ebp | -0.70 | 0.0000 |
| Porcn | 1.01 | 0.0001 |
| Tspan7 | 0.33 | 0.0221 |
| Atp6ap2 | -0.51 | 0.0002 |
| 1810030O07Rik | 0.27 | 0.0577 |
| Med14 | 0.81 | 0.0009 |
| Nyx | 1.57 | 0.0000 |
| Fundc1 | 0.62 | 0.0000 |
| Kdm6a | 0.81 | 0.0006 |
| 4930578C19Rik | 1.98 | 0.0000 |
| Slc9a7 | 2.01 | 0.0171 |
| Jade3 | -0.46 | 0.0137 |
| Usp11 | 0.51 | 0.0055 |
| Timp1 | -1.01 | 0.0000 |
| Cfp | -0.97 | 0.0021 |
| Uxt | -0.75 | 0.0183 |
| Zfp300 | 1.73 | 0.0004 |
| Il13ra1 | -1.25 | 0.0000 |
| Lonrf3 | -0.76 | 0.0828 |
| Pgrmc1 | 0.44 | 0.0050 |
| Akap17b | 0.86 | 0.0001 |
| Nkrf | -0.79 | 0.0000 |
| Sept6 | 1.19 | 0.0000 |
| Rhox5 | -1.48 | 0.0000 |
| Zbtb33 | 0.36 | 0.0772 |
| Gria3 | 0.59 | 0.0008 |
| Tenm1 | 1.31 | 0.0088 |
| Apln | -0.76 | 0.0003 |
| Xpnpep2 | -1.37 | 0.0686 |
| Zdhhc9 | -0.81 | 0.0000 |
| Utp14a | -0.90 | 0.0000 |
| Enox2 | 0.53 | 0.0796 |
| Mbnl3 | 1.74 | 0.0076 |
| Phf6 | 0.36 | 0.0997 |
| Hprt | -0.27 | 0.0817 |
| Fam122b | 1.22 | 0.0000 |
| Cxx1c | 0.97 | 0.0000 |
| Cxx1a | 0.47 | 0.0104 |
| Cxx1b | 0.67 | 0.0000 |
| Ddx26b | 0.84 | 0.0000 |
| Mmgt1 | 0.42 | 0.0178 |
| Htatsf1 | -0.26 | 0.0356 |
| Arhgef6 | 0.82 | 0.0000 |
| Ids | 0.62 | 0.0002 |
| BC023829 | 0.41 | 0.0116 |
| Mamld1 | 0.81 | 0.0000 |
| Mtmr1 | 0.55 | 0.0002 |
| Cd99l2 | 0.60 | 0.0038 |
| Prrg3 | 1.77 | 0.0273 |
| Nsdhl | -3.41 | 0.0000 |
| Abcd1 | 1.37 | 0.0000 |
| Irak1 | -0.29 | 0.0814 |
| Flna | -0.67 | 0.0011 |
| Emd | -0.30 | 0.0116 |
| Rpl10 | -0.99 | 0.0000 |
| Atp6ap1 | -0.30 | 0.0050 |
| Fam50a | -0.58 | 0.0018 |
| Lage3 | 0.40 | 0.0797 |
| Ubl4 | -0.31 | 0.0284 |
| G6pdx | -0.26 | 0.0199 |
| Gab3 | 0.90 | 0.0150 |
| Dkc1 | -0.61 | 0.0020 |
| Mpp1 | 0.32 | 0.0027 |
| F8 | 0.97 | 0.0001 |
| Mtcp1 | 0.61 | 0.0154 |
| Brcc3 | 0.30 | 0.0322 |
| Prkx | -0.36 | 0.0369 |
| Tmem47 | -0.79 | 0.0000 |
| Dmd | 1.24 | 0.0002 |
| Pdk3 | 1.19 | 0.0000 |
| Klhl15 | -0.45 | 0.0723 |
| Maged1 | 0.32 | 0.0029 |
| Spin4 | 0.85 | 0.0022 |
| Arhgef9 | 1.25 | 0.0000 |
| Amer1 | 0.44 | 0.1000 |
| Zc4h2 | 1.00 | 0.0000 |
| Las1l | -0.72 | 0.0000 |
| Msn | -0.57 | 0.0015 |
| Heph | 0.80 | 0.0102 |
| Eda2r | -0.50 | 0.0033 |
| Ar | 1.01 | 0.0000 |
| Ophn1 | 0.88 | 0.0000 |
| Stard8 | 0.65 | 0.0000 |
| Efnb1 | 0.62 | 0.0015 |
| Pja1 | 0.38 | 0.0402 |
| Foxo4 | 0.61 | 0.0000 |
| Gm614 | -2.05 | 0.0648 |
| Il2rg | -1.92 | 0.0000 |
| Nlgn3 | -0.95 | 0.0010 |
| Zmym3 | 0.70 | 0.0000 |
| Taf1 | -0.63 | 0.0001 |
| Ogt | -0.42 | 0.0054 |
| Nhsl2 | 0.98 | 0.0000 |
| Rgag4 | 1.29 | 0.0008 |
| Chic1 | 0.61 | 0.0005 |
| Slc16a2 | 1.66 | 0.0594 |
| C77370 | 1.61 | 0.0000 |
| Uprt | 1.00 | 0.0151 |
| Zdhhc15 | 1.55 | 0.0017 |
| Pbdc1 | -0.54 | 0.0007 |
| Magee1 | 0.87 | 0.0001 |
| Atrx | 0.39 | 0.0324 |
| Pgk1 | -0.74 | 0.0000 |
| Taf9b | 0.95 | 0.0000 |
| Brwd3 | 0.65 | 0.0018 |
| Hmgn5 | 0.41 | 0.0481 |
| Sh3bgrl | 0.28 | 0.0514 |
| Hdx | -0.78 | 0.0016 |
| Klhl4 | 1.28 | 0.0000 |
| Nap1l3 | 0.84 | 0.0010 |
| Diap2 | 0.64 | 0.0107 |
| Tspan6 | 0.35 | 0.0392 |
| Trmt2b | 0.84 | 0.0000 |
| Timm8a1 | -1.29 | 0.0000 |
| Rpl36a | -0.61 | 0.0522 |
| Armcx4 | 1.43 | 0.0000 |
| Fam199x | 0.61 | 0.0025 |
| Tbc1d8b | 0.95 | 0.0008 |
| Tsc22d3 | -1.22 | 0.0000 |
| Mid2 | 0.46 | 0.0262 |
| Atg4a | 0.42 | 0.0343 |
| Ammecr1 | 0.93 | 0.0000 |
| Alg13 | -0.53 | 0.0514 |
| Amot | 0.67 | 0.0010 |
| Lrch2 | 1.15 | 0.0075 |
| Apex2 | -0.97 | 0.0000 |
| Fgd1 | 1.00 | 0.0000 |
| Fam120c | 0.91 | 0.0466 |
| Phf8 | 0.55 | 0.0214 |
| Hsd17b10 | 0.43 | 0.0204 |
| Iqsec2 | 0.54 | 0.0006 |
| Tspyl2 | -0.87 | 0.0001 |
| Shroom2 | 1.28 | 0.0000 |
| Mageh1 | 0.98 | 0.0000 |
| Ubqln2 | 0.69 | 0.0028 |
| Spin2c | 1.58 | 0.0002 |
| Sms | -1.07 | 0.0001 |
| A830080D01Rik | -1.04 | 0.0000 |
| Sh3kbp1 | -0.24 | 0.0586 |
| Scml2 | 1.15 | 0.0500 |
| Nhs | 1.22 | 0.0000 |
| Reps2 | 2.00 | 0.0000 |
| Txlng | -0.46 | 0.0056 |
| Ap1s2 | -0.38 | 0.0113 |
| Zrsr2 | -0.43 | 0.0022 |
| Car5b | 0.71 | 0.0000 |
| Bmx | 1.17 | 0.0704 |
| Pir | 0.53 | 0.0022 |
| Fancb | 0.44 | 0.0467 |
| Rab9 | -0.69 | 0.0000 |
| Tmsb4x | -0.54 | 0.0117 |
| Prps2 | 0.86 | 0.0000 |
| Msl3 | 0.49 | 0.0300 |
| Arhgap6 | -0.35 | 0.0026 |
| Mid1 | 0.61 | 0.0228 |
| Pkia | 0.90 | 0.0014 |
| Hey1 | 1.67 | 0.0000 |
| Zbtb10 | -0.67 | 0.0000 |
| Zfp704 | -0.49 | 0.0178 |
| Zfand1 | 0.47 | 0.0162 |
| Chmp4c | -1.05 | 0.0001 |
| Snx16 | -0.69 | 0.0002 |
| Lrrcc1 | 0.26 | 0.0757 |
| 1810022K09Rik | -1.15 | 0.0010 |
| Car13 | -0.39 | 0.0885 |
| Car2 | -0.66 | 0.0005 |
| Pde7a | -0.71 | 0.0000 |
| Cp | -1.75 | 0.0029 |
| Hps3 | -0.33 | 0.0363 |
| Nceh1 | 0.60 | 0.0025 |
| Tnfsf10 | 2.29 | 0.0000 |
| Tnik | 0.94 | 0.0021 |
| Slc2a2 | 1.08 | 0.0147 |
| Eif5a2 | 0.73 | 0.0023 |
| Rpl22l1 | -1.07 | 0.0000 |
| Phc3 | 0.46 | 0.0156 |
| Zmat3 | 0.41 | 0.0192 |
| Gnb4 | 0.90 | 0.0000 |
| Mccc1 | 0.76 | 0.0001 |
| Exosc9 | -0.46 | 0.0056 |
| Ccna2 | 0.53 | 0.0021 |
| Bbs12 | -0.39 | 0.0224 |
| Spata5 | -0.96 | 0.0000 |
| Ankrd50 | 0.42 | 0.0885 |
| Fat4 | 1.06 | 0.0000 |
| Intu | 1.01 | 0.0016 |
| Mfsd8 | 0.65 | 0.0025 |
| Larp1b | -0.32 | 0.0541 |
| D3Ertd751e | 1.76 | 0.0000 |
| Slc7a11 | -2.35 | 0.0000 |
| Ccrn4l | -2.77 | 0.0000 |
| Naa15 | -0.54 | 0.0000 |
| Maml3 | 1.58 | 0.0000 |
| Foxo1 | -0.46 | 0.0239 |
| Cog6 | 0.52 | 0.0063 |
| Gm5641 | -0.38 | 0.0003 |
| Alg5 | -0.39 | 0.0262 |
| Nbea | 0.55 | 0.0634 |
| Tm4sf1 | -1.28 | 0.0000 |
| Tsc22d2 | -1.03 | 0.0000 |
| Erich6 | -1.56 | 0.0142 |
| Siah2 | -1.47 | 0.0000 |
| Rap2b | -0.70 | 0.0009 |
| Arhgef26 | 1.45 | 0.0000 |
| E130311K13Rik | 0.56 | 0.0125 |
| Tiparp | -0.80 | 0.0000 |
| Rsrc1 | 0.28 | 0.0601 |
| Ppm1l | 0.89 | 0.0004 |
| Nmd3 | -0.82 | 0.0000 |
| Pdcd10 | -0.47 | 0.0051 |
| Fnip2 | -0.39 | 0.0342 |
| Ppid | -0.68 | 0.0000 |
| Etfdh | 0.52 | 0.0062 |
| 4930579G24Rik | 0.44 | 0.0838 |
| Ctso | 0.64 | 0.0016 |
| Tlr2 | -1.08 | 0.0000 |
| D930015E06Rik | 0.44 | 0.0133 |
| Tmem154 | -1.10 | 0.0000 |
| Gatb | 0.40 | 0.0429 |
| Lrba | 0.57 | 0.0431 |
| Dclk2 | 0.41 | 0.0584 |
| Cd1d2 | 2.45 | 0.0000 |
| Cd1d1 | 1.16 | 0.0000 |
| Etv3 | -0.42 | 0.0042 |
| Lrrc71 | -1.00 | 0.0000 |
| Hdgf | -0.23 | 0.0941 |
| Rrnad1 | -0.40 | 0.0784 |
| Isg20l2 | -0.46 | 0.0005 |
| Nes | -0.88 | 0.0000 |
| Iqgap3 | -0.37 | 0.0140 |
| Cct3 | -0.80 | 0.0000 |
| Smg5 | -0.32 | 0.0034 |
| Lmna | -0.52 | 0.0000 |
| Ubqln4 | -0.34 | 0.0022 |
| 2810403A07Rik | -0.39 | 0.0051 |
| Msto1 | -0.33 | 0.0473 |
| Fdps | -5.58 | 0.0000 |
| Clk2 | -0.54 | 0.0203 |
| Fam189b | -0.80 | 0.0214 |
| Slc50a1 | 0.58 | 0.0036 |
| Efna1 | 1.25 | 0.0000 |
| Efna3 | 1.05 | 0.0000 |
| Efna4 | -1.31 | 0.0001 |
| Adam15 | -0.40 | 0.0093 |
| Dcst1 | 0.74 | 0.0039 |
| Shc1 | 0.36 | 0.0017 |
| Pbxip1 | 0.33 | 0.0017 |
| Pmvk | -3.04 | 0.0000 |
| She | 0.96 | 0.0000 |
| Atp8b2 | 0.36 | 0.0863 |
| Hax1 | -0.81 | 0.0000 |
| Tpm3 | -0.31 | 0.0270 |
| Nup210l | -0.97 | 0.0001 |
| Jtb | -0.55 | 0.0095 |
| Slc39a1 | -0.41 | 0.0001 |
| Crtc2 | -0.44 | 0.0001 |
| Ilf2 | -0.26 | 0.0513 |
| Snapin | 0.28 | 0.0732 |
| S100a13 | 0.39 | 0.0116 |
| Sprr2a1 | -3.40 | 0.0000 |
| Sprr2a2 | -2.41 | 0.0013 |
| Sprr2b | -4.79 | 0.0000 |
| Sprr1a | -2.30 | 0.0000 |
| Crct1 | -3.23 | 0.0639 |
| S100a11 | -0.95 | 0.0008 |
| Them4 | -0.64 | 0.0410 |
| Riiad1 | -1.18 | 0.0908 |
| Psmb4 | -0.25 | 0.0704 |
| Selenbp1 | 1.80 | 0.0000 |
| Zfp687 | 0.49 | 0.0086 |
| Pip5k1a | -0.41 | 0.0018 |
| Vps72 | -0.44 | 0.0395 |
| Lysmd1 | 0.92 | 0.0000 |
| Sema6c | 0.81 | 0.0696 |
| Mllt11 | -0.59 | 0.0000 |
| Prune | 0.50 | 0.0057 |
| Setdb1 | -0.36 | 0.0096 |
| Golph3l | 0.61 | 0.0010 |
| Mcl1 | -0.47 | 0.0000 |
| Tars2 | -0.48 | 0.0146 |
| Mtmr11 | -0.31 | 0.0424 |
| Txnip | 1.30 | 0.0000 |
| Polr3gl | 0.63 | 0.0000 |
| Pias3 | 0.64 | 0.0000 |
| Rnf115 | 0.27 | 0.0960 |
| Gpr89 | 0.36 | 0.0785 |
| Gja5 | 2.45 | 0.0000 |
| Acp6 | 0.91 | 0.0000 |
| Bcl9 | 0.71 | 0.0000 |
| Fmo5 | 1.79 | 0.0000 |
| Prkab2 | -0.53 | 0.0053 |
| Pde4dip | -0.77 | 0.0000 |
| Notch2 | -1.14 | 0.0001 |
| Phgdh | -0.77 | 0.0000 |
| Zfp697 | -0.35 | 0.0468 |
| Wars2 | -0.39 | 0.0668 |
| Wdr3 | -0.60 | 0.0013 |
| Igsf3 | -1.50 | 0.0000 |
| Ngf | -1.20 | 0.0233 |
| Tspan2 | 0.78 | 0.0034 |
| Csde1 | -0.25 | 0.0162 |
| Nras | -0.36 | 0.0004 |
| Dennd2c | 0.80 | 0.0077 |
| Ptpn22 | -2.43 | 0.0602 |
| Fam19a3 | -0.90 | 0.0000 |
| Ppm1j | -1.52 | 0.0000 |
| Rhoc | -1.20 | 0.0000 |
| Mov10 | 0.53 | 0.0011 |
| Capza1 | -0.23 | 0.0984 |
| Kcnd3 | -0.54 | 0.0076 |
| Wdr77 | -0.92 | 0.0000 |
| Ovgp1 | -0.97 | 0.0226 |
| Chil5 | 0.29 | 0.0498 |
| Chil3 | -1.31 | 0.0959 |
| Dram2 | 0.28 | 0.0524 |
| Lrif1 | -0.79 | 0.0000 |
| Slc16a4 | 1.45 | 0.0000 |
| Rbm15 | -0.68 | 0.0002 |
| Csf1 | -2.73 | 0.0000 |
| Gstm5 | 0.86 | 0.0090 |
| Gstm7 | 1.01 | 0.0056 |
| Gstm2 | 0.40 | 0.0417 |
| Amigo1 | 1.32 | 0.0060 |
| Psma5 | -0.54 | 0.0106 |
| Sort1 | 1.43 | 0.0000 |
| Sars | -0.81 | 0.0000 |
| Tmem167b | 0.68 | 0.0243 |
| Gpsm2 | 0.43 | 0.0329 |
| Stxbp3a | -0.48 | 0.0005 |
| Prpf38b | -0.55 | 0.0010 |
| Fam102b | 1.45 | 0.0000 |
| Prmt6 | -0.36 | 0.0350 |
| Rnpc3 | -0.49 | 0.0028 |
| S1pr1 | -0.91 | 0.0000 |
| Dph5 | -1.14 | 0.0000 |
| Slc30a7 | -0.31 | 0.0385 |
| Vcam1 | -4.27 | 0.0000 |
| Dbt | 0.76 | 0.0004 |
| Sass6 | 0.54 | 0.0596 |
| Hiat1 | -0.47 | 0.0017 |
| Agl | 1.28 | 0.0000 |
| Palmd | 1.29 | 0.0000 |
| Ptbp2 | -0.68 | 0.0189 |
| Cnn3 | -0.46 | 0.0000 |
| Abca4 | -1.03 | 0.0000 |
| Gclm | -0.75 | 0.0000 |
| Dnttip2 | -0.63 | 0.0000 |
| Bcar3 | -1.13 | 0.0000 |
| Camk2d | -0.39 | 0.0028 |
| Larp7 | -0.40 | 0.0075 |
| Zgrf1 | 0.42 | 0.0095 |
| Alpk1 | -1.07 | 0.0000 |
| Elovl6 | -2.37 | 0.0000 |
| Gar1 | -0.93 | 0.0000 |
| Pla2g12a | 0.39 | 0.0790 |
| Casp6 | 1.03 | 0.0000 |
| Rpl34 | -0.85 | 0.0009 |
| Hadh | 1.45 | 0.0000 |
| Cyp2u1 | 1.67 | 0.0675 |
| Sgms2 | -1.52 | 0.0000 |
| Papss1 | 0.34 | 0.0040 |
| Dkk2 | 2.33 | 0.0000 |
| Npnt | 0.45 | 0.0767 |
| Ppa2 | 0.39 | 0.0508 |
| Tet2 | 0.34 | 0.0473 |
| Cxxc4 | 1.15 | 0.0068 |
| Bdh2 | 2.56 | 0.0299 |
| Ube2d3 | -0.45 | 0.0000 |
| Nfkb1 | -0.50 | 0.0029 |
| Ppp3ca | 0.33 | 0.0182 |
| Ddit4l | 1.17 | 0.0000 |
| Dnajb14 | 0.33 | 0.0136 |
| Lamtor3 | -0.34 | 0.0155 |
| Dapp1 | 0.67 | 0.0053 |
| Eif4e | -0.44 | 0.0004 |
| Tspan5 | 0.30 | 0.0491 |
| Pdlim5 | -1.12 | 0.0000 |
| Gbp5 | -1.45 | 0.0000 |
| Gbp2 | -0.95 | 0.0018 |
| Ccbl2 | 1.77 | 0.0000 |
| Hs2st1 | 0.47 | 0.0141 |
| Clca3 | 1.72 | 0.0932 |
| Znhit6 | -0.93 | 0.0000 |
| Cyr61 | -2.60 | 0.0000 |
| Ddah1 | -1.52 | 0.0000 |
| Bcl10 | -1.35 | 0.0000 |
| 2410004B18Rik | -0.59 | 0.0142 |
| Mcoln2 | -1.58 | 0.0002 |
| Gng5 | -0.53 | 0.0264 |
| Rpf1 | -0.61 | 0.0008 |
| Prkacb | 0.36 | 0.0704 |
| Lphn2 | -1.15 | 0.0000 |
| Eltd1 | 0.99 | 0.0000 |
| Ifi44 | -0.53 | 0.0511 |
| Fubp1 | -0.52 | 0.0002 |
| Ak5 | 0.92 | 0.0000 |
| Pigk | 0.35 | 0.0015 |
| St6galnac3 | 1.79 | 0.0000 |
| Asb17 | 4.07 | 0.0031 |
| Msh4 | 1.68 | 0.0000 |
| Rabggtb | -0.87 | 0.0000 |
| Acadm | 0.24 | 0.0927 |
| Cryz | 1.13 | 0.0000 |
| Zranb2 | -0.30 | 0.0156 |
| Cth | -2.05 | 0.0000 |
| Srsf11 | -0.51 | 0.0028 |
| Wls | 0.37 | 0.0276 |
| Tgs1 | -0.32 | 0.0502 |
| Lyn | 0.42 | 0.0166 |
| Plag1 | 1.24 | 0.0000 |
| Ubxn2b | 0.59 | 0.0008 |
| Sdcbp | -0.32 | 0.0647 |
| Tox | 2.56 | 0.0000 |
| Rab2a | -0.30 | 0.0066 |
| Chd7 | -0.76 | 0.0000 |
| Asph | 1.53 | 0.0000 |
| 2610301B20Rik | 0.67 | 0.0037 |
| Plekhf2 | -0.40 | 0.0096 |
| Ccne2 | 0.64 | 0.0003 |
| Dpy19l4 | 0.47 | 0.0629 |
| Gem | 1.79 | 0.0000 |
| Tmem67 | 0.82 | 0.0000 |
| Triqk | 1.73 | 0.0609 |
| Tmem55a | -0.42 | 0.0004 |
| Tmem64 | 1.37 | 0.0000 |
| Decr1 | 0.71 | 0.0000 |
| Nbn | -0.34 | 0.0658 |
| Osgin2 | -0.63 | 0.0002 |
| Ripk2 | -2.72 | 0.0000 |
| Cpne3 | 0.33 | 0.0174 |
| Ggh | 0.48 | 0.0503 |
| Ccnc | -0.40 | 0.0048 |
| Tstd3 | 0.62 | 0.0174 |
| Mms22l | 0.49 | 0.0093 |
| Ndufaf4 | -0.71 | 0.0002 |
| Ufl1 | 0.24 | 0.0898 |
| Mdn1 | -1.04 | 0.0000 |
| Ube2j1 | -0.42 | 0.0022 |
| Pnrc1 | -1.14 | 0.0000 |
| Akirin2 | -0.49 | 0.0002 |
| Slc35a1 | 0.59 | 0.0006 |
| 3110043O21Rik | -0.45 | 0.0013 |
| Aco1 | -0.61 | 0.0063 |
| Topors | -0.24 | 0.0749 |
| Smu1 | -0.33 | 0.0024 |
| B4galt1 | -0.77 | 0.0000 |
| Bag1 | -0.32 | 0.0858 |
| Nfx1 | -0.25 | 0.0820 |
| Aqp7 | -2.38 | 0.0253 |
| Ubap2 | -0.33 | 0.0079 |
| Dcaf12 | -0.24 | 0.0589 |
| Ubap1 | -0.82 | 0.0000 |
| AI464131 | 1.20 | 0.0988 |
| 1110017D15Rik | -1.33 | 0.0160 |
| Dnaic1 | 1.84 | 0.0000 |
| Enho | 1.92 | 0.0000 |
| Gm13305 | 0.78 | 0.0972 |
| 1700022I11Rik | -1.41 | 0.0518 |
| Vcp | -0.48 | 0.0048 |
| Fancg | 0.81 | 0.0000 |
| Ccdc107 | -0.61 | 0.0308 |
| Arhgef39 | 0.81 | 0.0024 |
| Gba2 | 0.34 | 0.0876 |
| Hint2 | 0.52 | 0.0191 |
| Tmem8b | 0.74 | 0.0000 |
| Hrct1 | 1.15 | 0.0000 |
| Reck | 0.78 | 0.0097 |
| Glipr2 | -1.68 | 0.0000 |
| Zcchc7 | -0.52 | 0.0153 |
| Grhpr | 1.07 | 0.0000 |
| Fbxo10 | 1.17 | 0.0000 |
| Tomm5 | -0.42 | 0.0872 |
| Shb | -0.67 | 0.0000 |
| Tdrd7 | -0.34 | 0.0333 |
| 5830415F09Rik | -0.91 | 0.0004 |
| Anp32b | -0.89 | 0.0000 |
| Nans | 0.37 | 0.0374 |
| Tbc1d2 | -0.43 | 0.0082 |
| Anks6 | 0.94 | 0.0000 |
| Tgfbr1 | -0.46 | 0.0122 |
| Alg2 | 0.72 | 0.0000 |
| Nr4a3 | -0.93 | 0.0215 |
| Stx17 | 0.69 | 0.0000 |
| Invs | 0.36 | 0.0774 |
| Nipsnap3b | -0.58 | 0.0000 |
| Abca1 | 2.72 | 0.0000 |
| Slc44a1 | 0.77 | 0.0000 |
| Tmem38b | 0.56 | 0.0091 |
| Klf4 | -0.98 | 0.0000 |
| Ctnnal1 | 0.49 | 0.0185 |
| Tmem245 | 0.36 | 0.0779 |
| Txn1 | -0.50 | 0.0017 |
| Gng10 | 0.97 | 0.0076 |
| Ugcg | -0.76 | 0.0000 |
| Susd1 | 1.13 | 0.0000 |
| Hsdl2 | 1.06 | 0.0000 |
| Slc31a1 | -0.54 | 0.0021 |
| Wdr31 | 1.59 | 0.0111 |
| Bspry | 1.41 | 0.0036 |
| Hdhd3 | 0.81 | 0.0000 |
| Pole3 | 0.29 | 0.0514 |
| 4933430I17Rik | 1.57 | 0.0007 |
| Rgs3 | 0.82 | 0.0000 |
| Col27a1 | -0.69 | 0.0003 |
| Orm2 | -0.93 | 0.0053 |
| Akna | -0.31 | 0.0963 |
| Atp6v1g1 | -0.56 | 0.0293 |
| Tnc | -2.10 | 0.0000 |
| Tlr4 | -0.65 | 0.0086 |
| Cdk5rap2 | 0.32 | 0.0702 |
| Megf9 | 0.89 | 0.0006 |
| Lurap1l | 1.24 | 0.0000 |
| Nfib | -0.56 | 0.0000 |
| Cntln | 0.72 | 0.0063 |
| Adamtsl1 | 1.60 | 0.0000 |
| Plin2 | -0.32 | 0.0154 |
| Acer2 | 1.14 | 0.0000 |
| Mllt3 | 0.86 | 0.0000 |
| Ptplad2 | 2.28 | 0.0000 |
| Mtap | -0.78 | 0.0000 |
| Cdkn2b | -1.49 | 0.0000 |
| Tek | 0.55 | 0.0001 |
| Jun | -1.23 | 0.0000 |
| Fggy | 0.69 | 0.0046 |
| Cyp2j6 | 0.35 | 0.0303 |
| Nfia | 1.64 | 0.0000 |
| Kank4 | 2.08 | 0.0040 |
| Alg6 | 1.26 | 0.0000 |
| Itgb3bp | 0.50 | 0.0850 |
| Pgm2 | 0.27 | 0.0507 |
| Cachd1 | -0.79 | 0.0000 |
| Leprot | 0.22 | 0.0774 |
| Pde4b | -1.06 | 0.0000 |
| Sgip1 | 0.72 | 0.0000 |
| Slc35d1 | -0.36 | 0.0161 |
| Ppap2b | 0.68 | 0.0000 |
| Dhcr24 | -4.81 | 0.0000 |
| Acot11 | 2.63 | 0.0001 |
| Ssbp3 | 0.99 | 0.0000 |
| Cyb5rl | 0.70 | 0.0001 |
| Tmem59 | 0.50 | 0.0016 |
| Yipf1 | 0.42 | 0.0020 |
| Glis1 | -0.80 | 0.0001 |
| Lrp8 | -2.22 | 0.0000 |
| Magoh | -0.45 | 0.0538 |
| 0610037L13Rik | -0.49 | 0.0007 |
| Cpt2 | 0.75 | 0.0000 |
| Podn | 1.22 | 0.0057 |
| Scp2 | 0.64 | 0.0001 |
| Zcchc11 | 0.81 | 0.0083 |
| Cc2d1b | -0.47 | 0.0135 |
| Zfyve9 | 0.33 | 0.0454 |
| Txndc12 | 0.37 | 0.0161 |
| Kti12 | -0.31 | 0.0229 |
| Osbpl9 | -0.23 | 0.0281 |
| Ttc39a | 1.02 | 0.0000 |
| Spata6 | 0.43 | 0.0010 |
| Slc5a9 | 2.21 | 0.0001 |
| Efcab14 | 0.54 | 0.0053 |
| Atpaf1 | 0.57 | 0.0004 |
| Nsun4 | -0.58 | 0.0008 |
| Pomgnt1 | -0.36 | 0.0099 |
| Pik3r3 | 0.78 | 0.0000 |
| Mast2 | -0.35 | 0.0040 |
| Ipp | 1.02 | 0.0000 |
| Toe1 | -0.39 | 0.0426 |
| Urod | 0.99 | 0.0000 |
| Hectd3 | 0.53 | 0.0000 |
| Eif2b3 | -0.63 | 0.0000 |
| Ptch2 | 2.28 | 0.0000 |
| Plk3 | -1.02 | 0.0000 |
| Rps8 | -0.65 | 0.0459 |
| Tmem53 | 1.45 | 0.0044 |
| Rnf220 | -0.33 | 0.0207 |
| Slc6a9 | -1.56 | 0.0000 |
| Ccdc24 | -1.47 | 0.0346 |
| Atp6v0b | -0.58 | 0.0009 |
| Dph2 | -0.87 | 0.0000 |
| Ipo13 | -0.31 | 0.0257 |
| St3gal3 | 0.71 | 0.0000 |
| Med8 | -0.70 | 0.0001 |
| Elovl1 | -0.74 | 0.0000 |
| Cdc20 | 0.65 | 0.0000 |
| Tie1 | 0.46 | 0.0000 |
| Ebna1bp2 | -0.91 | 0.0000 |
| Slc2a1 | -1.46 | 0.0000 |
| Ccdc30 | 0.84 | 0.0989 |
| Ppcs | 0.49 | 0.0013 |
| Hivep3 | 0.60 | 0.0903 |
| Ctps | -1.32 | 0.0000 |
| Ppt1 | 0.69 | 0.0000 |
| Mfsd2a | -4.50 | 0.0000 |
| Trit1 | -0.51 | 0.0380 |
| Ppie | -0.43 | 0.0124 |
| Rragc | -0.52 | 0.0000 |
| Utp11l | -0.37 | 0.0177 |
| Fhl3 | -1.27 | 0.0000 |
| Yrdc | -1.08 | 0.0000 |
| Gnl2 | -1.04 | 0.0000 |
| Zc3h12a | -2.35 | 0.0000 |
| Oscp1 | 1.85 | 0.0000 |
| Stk40 | -1.01 | 0.0000 |
| Sh3d21 | -0.94 | 0.0418 |
| Map7d1 | -0.42 | 0.0000 |
| Adprhl2 | -0.42 | 0.0642 |
| Tekt2 | -0.99 | 0.0758 |
| Ago1 | 0.80 | 0.0000 |
| Ago4 | 0.97 | 0.0390 |
| Clspn | 0.42 | 0.0156 |
| Ncdn | -0.31 | 0.0608 |
| Sfpq | -0.28 | 0.0183 |
| Gja4 | 0.93 | 0.0000 |
| A3galt2 | -1.35 | 0.0001 |
| Zfp362 | 0.90 | 0.0000 |
| Trim62 | 1.62 | 0.0000 |
| Ak2 | -0.39 | 0.0601 |
| Yars | -0.93 | 0.0000 |
| C77080 | -0.35 | 0.0012 |
| Rbbp4 | 0.35 | 0.0220 |
| Marcksl1 | -1.16 | 0.0000 |
| Fam167b | 1.12 | 0.0000 |
| Ccdc28b | 0.95 | 0.0006 |
| Nkain1 | -1.26 | 0.0000 |
| Tmem200b | -1.56 | 0.0005 |
| Ythdf2 | -0.31 | 0.0172 |
| Trnau1ap | -0.59 | 0.0062 |
| Rcc1 | -0.88 | 0.0000 |
| Phactr4 | -0.56 | 0.0016 |
| Sesn2 | -0.33 | 0.0127 |
| Eya3 | 0.33 | 0.0343 |
| Xkr8 | 0.44 | 0.0356 |
| Rpa2 | 0.33 | 0.0311 |
| Fam76a | -0.40 | 0.0015 |
| Gpr3 | -2.07 | 0.0781 |
| Map3k6 | 0.40 | 0.0481 |
| Wdtc1 | 0.74 | 0.0001 |
| Gpn2 | -0.43 | 0.0704 |
| Sfn | -1.18 | 0.0016 |
| Zdhhc18 | -0.69 | 0.0001 |
| Rps6ka1 | 1.18 | 0.0000 |
| Dhdds | 0.43 | 0.0104 |
| Zfp593 | -1.37 | 0.0208 |
| Grrp1 | 1.25 | 0.0000 |
| Stmn1 | 0.71 | 0.0000 |
| Man1c1 | 1.08 | 0.0000 |
| Ldlrap1 | 0.59 | 0.0031 |
| Rsrp1 | -0.95 | 0.0000 |
| Clic4 | -0.74 | 0.0001 |
| Rcan3 | 1.10 | 0.0002 |
| Nipal3 | 1.14 | 0.0000 |
| Srsf10 | -0.49 | 0.0000 |
| Fuca1 | 0.26 | 0.0674 |
| Lypla2 | -0.69 | 0.0010 |
| Id3 | -0.50 | 0.0375 |
| E2f2 | 0.67 | 0.0151 |
| Asap3 | 1.94 | 0.0000 |
| Rap1gap | 0.79 | 0.0017 |
| Alpl | 0.51 | 0.0006 |
| Ece1 | -0.35 | 0.0964 |
| Sh2d5 | -2.96 | 0.0000 |
| Kif17 | -2.39 | 0.0000 |
| Pink1 | 0.59 | 0.0000 |
| Cda | 0.86 | 0.0000 |
| Mrto4 | -1.02 | 0.0000 |
| Iffo2 | -0.76 | 0.0000 |
| Aldh4a1 | 0.37 | 0.0449 |
| Atp13a2 | 0.74 | 0.0000 |
| Crocc | 0.79 | 0.0004 |
| Fbxo42 | -0.55 | 0.0001 |
| Epha2 | -0.87 | 0.0000 |
| Zbtb17 | -0.57 | 0.0002 |
| Plekhm2 | -0.40 | 0.0024 |
| Efhd2 | -0.31 | 0.0600 |
| Kazn | -1.66 | 0.0464 |
| Dhrs3 | 0.76 | 0.0012 |
| Vps13d | 0.47 | 0.0758 |
| Tnfrsf8 | -1.36 | 0.0859 |
| Fv1 | 1.01 | 0.0381 |
| Plod1 | 0.36 | 0.0326 |
| 2510039O18Rik | -0.34 | 0.0229 |
| Clcn6 | -1.36 | 0.0000 |
| Agtrap | 0.99 | 0.0000 |
| Exosc10 | -0.36 | 0.0263 |
| Srm | -1.05 | 0.0000 |
| Masp2 | -1.19 | 0.0009 |
| Tardbp | -0.24 | 0.0293 |
| Casz1 | 0.73 | 0.0142 |
| Pgd | -1.20 | 0.0000 |
| Ube4b | 0.40 | 0.0199 |
| Rbp7 | 0.85 | 0.0019 |
| Slc25a33 | -0.79 | 0.0010 |
| H6pd | 0.75 | 0.0000 |
| Eno1 | -0.54 | 0.0501 |
| Dnajc11 | -0.29 | 0.0198 |
| Zbtb48 | -0.64 | 0.0357 |
| Nol9 | -0.28 | 0.0395 |
| Plekhg5 | 0.41 | 0.0086 |
| Espn | 0.68 | 0.0043 |
| Acot7 | -0.90 | 0.0000 |
| Gpr153 | 0.95 | 0.0000 |
| Chd5 | -1.96 | 0.0000 |
| Kcnab2 | 2.62 | 0.0466 |
| Tprgl | 0.26 | 0.0634 |
| Megf6 | 1.47 | 0.0000 |
| Arhgef16 | 1.95 | 0.0835 |
| Fam213b | 1.22 | 0.0047 |
| Morn1 | 0.87 | 0.0557 |
| Prkcz | 0.77 | 0.0000 |
| Cdk11b | -0.51 | 0.0100 |
| Mmp23 | -0.92 | 0.0680 |
| Mib2 | -0.42 | 0.0161 |
| B930041F14Rik | 3.11 | 0.0000 |
| Atad3a | -0.99 | 0.0000 |
| Vwa1 | 1.01 | 0.0000 |
| Ccnl2 | -0.52 | 0.0170 |
| Mxra8 | 1.99 | 0.0321 |
| Dvl1 | -0.79 | 0.0000 |
| Tas1r3 | -0.56 | 0.0172 |
| Cptp | 0.68 | 0.0006 |
| Pusl1 | -0.57 | 0.0208 |
| Acap3 | 0.74 | 0.0000 |
| Fam132a | -1.21 | 0.0008 |
| B3galt6 | -0.57 | 0.0020 |
| Tnfrsf18 | 2.24 | 0.0969 |
| Isg15 | -0.61 | 0.0016 |
| Noc2l | -1.22 | 0.0000 |
| Fam133b | -0.66 | 0.0000 |
| Pex1 | 0.56 | 0.0013 |
| Gatad1 | 0.40 | 0.0021 |
| Krit1 | -0.30 | 0.0176 |
| Lrrd1 | -1.14 | 0.0772 |
| Cyp51 | -5.40 | 0.0000 |
| Gtpbp10 | -1.03 | 0.0000 |
| Steap4 | -4.79 | 0.0049 |
| Sri | -0.30 | 0.0955 |
| Adam22 | -1.06 | 0.0041 |
| Dbf4 | -0.36 | 0.0144 |
| Abcb1a | -0.72 | 0.0071 |
| Abcb1b | -1.03 | 0.0000 |
| Crot | 0.78 | 0.0000 |
| Dmtf1 | -0.41 | 0.0006 |
| Sema3e | -0.65 | 0.0350 |
| Sema3c | -1.14 | 0.0000 |
| Gnai1 | 0.99 | 0.0013 |
| Rsbn1l | -0.31 | 0.0111 |
| Ptpn12 | -0.62 | 0.0000 |
| Fam185a | -0.39 | 0.0464 |
| Dnajc2 | -0.73 | 0.0000 |
| Psmc2 | -0.42 | 0.0075 |
| Srpk2 | 0.33 | 0.0014 |
| Klhl7 | 0.33 | 0.0294 |
| Kcnh2 | -0.34 | 0.0417 |
| Nos3 | -0.76 | 0.0000 |
| Atg9b | -1.90 | 0.0000 |
| Asic3 | -0.63 | 0.0874 |
| Fastk | -0.54 | 0.0217 |
| Agap3 | -0.43 | 0.0099 |
| Abcf2 | -0.40 | 0.0076 |
| Crygn | 2.42 | 0.0588 |
| Rheb | -0.63 | 0.0000 |
| Prkag2 | 1.59 | 0.0000 |
| Kmt2c | 0.49 | 0.0350 |
| Xrcc2 | 0.65 | 0.0020 |
| Insig1 | -3.73 | 0.0000 |
| En2 | 1.22 | 0.0264 |
| Rnf32 | -0.69 | 0.0009 |
| Lmbr1 | 0.30 | 0.0778 |
| Nom1 | -0.59 | 0.0000 |
| Dnajb6 | -0.27 | 0.0775 |
| Il6 | -5.19 | 0.0000 |
| Ept1 | -0.92 | 0.0000 |
| Drc1 | -3.20 | 0.0001 |
| Slc35f6 | -0.23 | 0.0599 |
| Cenpa | 0.49 | 0.0004 |
| Mapre3 | 0.60 | 0.0017 |
| Tmem214 | -0.26 | 0.0251 |
| Agbl5 | 0.95 | 0.0000 |
| Khk | 0.88 | 0.0001 |
| Tcf23 | 1.16 | 0.0214 |
| Atraid | 0.52 | 0.0000 |
| Zfp513 | -0.61 | 0.0013 |
| Ift172 | 0.57 | 0.0000 |
| Zfp512 | -0.36 | 0.0012 |
| Supt7l | 0.30 | 0.0729 |
| Slc4a1ap | -0.33 | 0.0018 |
| Fosl2 | -1.55 | 0.0000 |
| Pisd | -0.82 | 0.0000 |
| Depdc5 | 0.54 | 0.0040 |
| Ywhah | -0.42 | 0.0000 |
| Slc5a1 | 1.66 | 0.0002 |
| Uvssa | 0.49 | 0.0000 |
| Fam53a | -0.37 | 0.0088 |
| Nat8l | -1.80 | 0.0601 |
| Haus3 | -0.36 | 0.0977 |
| Mxd4 | 1.39 | 0.0000 |
| Tnip2 | -0.56 | 0.0001 |
| Add1 | 0.37 | 0.0905 |
| Nop14 | -0.99 | 0.0000 |
| Grk4 | 0.57 | 0.0110 |
| Rgs12 | 0.48 | 0.0100 |
| Lrpap1 | 0.44 | 0.0004 |
| Acox3 | 0.49 | 0.0000 |
| Afap1 | -0.33 | 0.0818 |
| Tbc1d14 | 0.47 | 0.0773 |
| Bloc1s4 | -0.35 | 0.0866 |
| Man2b2 | 0.36 | 0.0003 |
| Wfs1 | 0.42 | 0.0063 |
| Crmp1 | -1.43 | 0.0000 |
| Evc | 1.31 | 0.0000 |
| Msx1 | -0.85 | 0.0000 |
| Stx18 | 0.26 | 0.0678 |
| Otop1 | -1.40 | 0.0018 |
| Slc2a9 | 1.24 | 0.0000 |
| Wdr1 | -0.38 | 0.0033 |
| Cpeb2 | -0.42 | 0.0484 |
| Cc2d2a | 1.37 | 0.0000 |
| Fbxl5 | -0.52 | 0.0000 |
| Cd38 | 0.73 | 0.0000 |
| Prom1 | 1.57 | 0.0000 |
| Ldb2 | 2.44 | 0.0000 |
| Lap3 | -0.45 | 0.0001 |
| Gpr125 | 0.35 | 0.0693 |
| Pi4k2b | -0.44 | 0.0009 |
| Smim20 | 0.44 | 0.0915 |
| Pcdh7 | -1.65 | 0.0000 |
| Pgm1 | -1.03 | 0.0000 |
| Tbc1d1 | -0.99 | 0.0000 |
| Fam114a1 | 0.58 | 0.0000 |
| Klhl5 | 0.52 | 0.0000 |
| Wdr19 | 1.28 | 0.0000 |
| Rpl9 | -1.09 | 0.0000 |
| Lias | 0.30 | 0.0286 |
| Smim14 | 0.73 | 0.0000 |
| Ube2k | -0.29 | 0.0122 |
| Apbb2 | 0.27 | 0.0203 |
| Uchl1 | -0.93 | 0.0004 |
| Nfxl1 | -0.43 | 0.0046 |
| Tec | -0.52 | 0.0041 |
| Slc10a4 | 0.86 | 0.0000 |
| Fryl | 0.71 | 0.0005 |
| Ociad2 | 1.30 | 0.0000 |
| Dcun1d4 | 0.53 | 0.0028 |
| Usp46 | 0.41 | 0.0549 |
| Rasl11b | -1.81 | 0.0000 |
| Scfd2 | 0.39 | 0.0250 |
| Fip1l1 | -0.21 | 0.0620 |
| Chic2 | -0.96 | 0.0000 |
| Kit | -0.44 | 0.0138 |
| Kdr | 0.58 | 0.0259 |
| Srd5a3 | 0.35 | 0.0073 |
| Arl9 | -1.06 | 0.0219 |
| Noa1 | -0.30 | 0.0184 |
| Igfbp7 | -0.80 | 0.0027 |
| Lphn3 | 0.99 | 0.0000 |
| Tmprss11f | -1.78 | 0.0001 |
| Ythdc1 | -0.47 | 0.0000 |
| Utp3 | -0.39 | 0.0109 |
| Grsf1 | 0.36 | 0.0382 |
| Dck | 1.10 | 0.0000 |
| Slc4a4 | 1.21 | 0.0138 |
| Afp | -1.64 | 0.0000 |
| Cxcl5 | -7.45 | 0.0000 |
| Pf4 | -1.41 | 0.0049 |
| Cxcl3 | -5.57 | 0.0012 |
| Cxcl1 | -6.00 | 0.0000 |
| Cxcl2 | -7.35 | 0.0000 |
| Ereg | -1.14 | 0.0000 |
| Areg | -2.65 | 0.0000 |
| Uso1 | -0.24 | 0.0520 |
| Sdad1 | -1.00 | 0.0000 |
| Cxcl10 | -1.81 | 0.0000 |
| Nup54 | -0.41 | 0.0540 |
| Scarb2 | 0.79 | 0.0001 |
| Ccni | 0.26 | 0.0276 |
| Ccng2 | 0.43 | 0.0004 |
| Cnot6l | 0.78 | 0.0001 |
| Anxa3 | -0.46 | 0.0000 |
| Bmp2k | -0.45 | 0.0028 |
| Hnrnpd | -0.58 | 0.0005 |
| Hnrnpdl | -0.68 | 0.0000 |
| Cops4 | 0.46 | 0.0034 |
| Plac8 | 0.84 | 0.0133 |
| Coq2 | -0.27 | 0.0408 |
| Agpat9 | -0.88 | 0.0000 |
| Cds1 | 2.10 | 0.0063 |
| Wdfy3 | 0.58 | 0.0237 |
| Aff1 | -0.99 | 0.0001 |
| Klhl8 | 0.37 | 0.0283 |
| Hsd17b13 | 2.50 | 0.0000 |
| Hsd17b11 | 0.86 | 0.0000 |
| Sparcl1 | 1.31 | 0.0000 |
| Spp1 | -1.71 | 0.0000 |
| Pkd2 | -0.28 | 0.0704 |
| Abcg3 | 4.97 | 0.0040 |
| Gbp9 | 0.53 | 0.0239 |
| Gbp4 | 0.43 | 0.0029 |
| Lrrc8c | -1.00 | 0.0000 |
| Zfp326 | -0.35 | 0.0038 |
| Zfp644 | -0.25 | 0.0668 |
| Hfm1 | -1.36 | 0.0578 |
| Tgfbr3 | 1.42 | 0.0000 |
| Brdt | 1.52 | 0.0002 |
| Rpl5 | -0.58 | 0.0779 |
| Ccdc18 | 0.52 | 0.0248 |
| Dr1 | -0.31 | 0.0044 |
| Mfsd7a | 1.69 | 0.0000 |
| Slc26a1 | 0.95 | 0.0269 |
| Fgfrl1 | 0.45 | 0.0109 |
| Crlf2 | -0.58 | 0.0007 |
| Plcxd1 | -0.95 | 0.0060 |
| Gtpbp6 | -0.82 | 0.0000 |
| Noc4l | -0.61 | 0.0001 |
| Pus1 | -0.92 | 0.0000 |
| Ulk1 | 0.62 | 0.0000 |
| Hscb | 0.72 | 0.0079 |
| Chek2 | 1.14 | 0.0000 |
| Ttc28 | 0.95 | 0.0000 |
| Mn1 | 1.02 | 0.0000 |
| 2900026A02Rik | 0.46 | 0.0503 |
| Sgsm1 | 1.35 | 0.0000 |
| Cmklr1 | 1.92 | 0.0000 |
| Ficd | 0.55 | 0.0003 |
| Tmem119 | 5.37 | 0.0000 |
| Ssh1 | -0.22 | 0.0920 |
| Svop | 1.16 | 0.0001 |
| Ung | 0.75 | 0.0102 |
| Kctd10 | 0.59 | 0.0024 |
| Ube3b | 0.34 | 0.0498 |
| Mmab | -1.41 | 0.0000 |
| Mvk | -2.36 | 0.0000 |
| Fam222a | 1.92 | 0.0000 |
| Gltp | 0.86 | 0.0000 |
| Tchp | 0.34 | 0.0511 |
| Ankrd13a | 0.52 | 0.0006 |
| Unc119b | 0.65 | 0.0000 |
| Mlec | 0.44 | 0.0262 |
| Cabp1 | -1.35 | 0.0000 |
| Gatc | 0.48 | 0.0011 |
| Cox6a1 | -0.33 | 0.0803 |
| Pxn | -0.25 | 0.0621 |
| Rab35 | -0.37 | 0.0029 |
| Ccdc64 | 0.94 | 0.0000 |
| Cit | 0.39 | 0.0524 |
| Prkab1 | 0.27 | 0.0221 |
| Suds3 | 0.40 | 0.0004 |
| Vsig10 | 0.58 | 0.0022 |
| Wsb2 | -0.33 | 0.0441 |
| Fbxo21 | 0.83 | 0.0000 |
| Rnft2 | 0.85 | 0.0675 |
| 2410131K14Rik | 0.51 | 0.0287 |
| Tbx3 | -0.48 | 0.0880 |
| Rbm19 | -0.62 | 0.0000 |
| Sds | -0.51 | 0.0719 |
| Slc8b1 | -0.32 | 0.0252 |
| Tpcn1 | 0.75 | 0.0000 |
| Ddx54 | -0.65 | 0.0000 |
| Oas3 | -1.86 | 0.0013 |
| Oas1a | -0.40 | 0.0656 |
| Gm15800 | 0.62 | 0.0095 |
| Naa25 | -0.57 | 0.0370 |
| Adam1a | -0.52 | 0.0186 |
| Mapkapk5 | -0.39 | 0.0134 |
| Aldh2 | 0.33 | 0.0028 |
| Acad12 | 1.22 | 0.0000 |
| Ppp1cc | -0.35 | 0.0025 |
| Rad9b | 0.39 | 0.0781 |
| Vps29 | -0.22 | 0.0997 |
| Fam216a | 0.62 | 0.0000 |
| Ift81 | 0.37 | 0.0119 |
| P2rx7 | 0.48 | 0.0119 |
| Camkk2 | 0.66 | 0.0000 |
| Rnf34 | 0.29 | 0.0399 |
| Kdm2b | 0.46 | 0.0229 |
| Orai1 | -0.80 | 0.0000 |
| Morn3 | -1.43 | 0.0073 |
| Tmem120b | -0.77 | 0.0000 |
| Rhof | -0.46 | 0.0278 |
| Setd1b | 0.33 | 0.0848 |
| Psmd9 | 0.32 | 0.0160 |
| Bcl7a | 0.72 | 0.0000 |
| Vps33a | 0.44 | 0.0288 |
| Rsrc2 | -0.43 | 0.0050 |
| Denr | -0.32 | 0.0468 |
| Hip1r | -0.90 | 0.0000 |
| Vps37b | -1.08 | 0.0000 |
| Pitpnm2 | 0.50 | 0.0021 |
| Mphosph9 | 0.76 | 0.0000 |
| 2810006K23Rik | 0.89 | 0.0000 |
| Setd8 | -0.53 | 0.0000 |
| Rilpl2 | 0.68 | 0.0006 |
| Rilpl1 | 0.74 | 0.0000 |
| Dnah10 | -0.93 | 0.0000 |
| Ccdc92 | 0.93 | 0.0000 |
| Ubc | -1.09 | 0.0000 |
| Dhx37 | -0.71 | 0.0000 |
| Bri3bp | -0.87 | 0.0000 |
| Aacs | -2.95 | 0.0000 |
| Slc15a4 | -0.51 | 0.0008 |
| Ran | -0.51 | 0.0000 |
| Sfswap | -0.40 | 0.0186 |
| Gbas | 0.34 | 0.0192 |
| Psph | -1.17 | 0.0000 |
| Cct6a | -0.49 | 0.0000 |
| Gusb | 0.79 | 0.0000 |
| Sbds | -0.70 | 0.0002 |
| Tyw1 | -0.24 | 0.0733 |
| Gatsl2 | 1.21 | 0.0000 |
| Wbscr16 | -0.60 | 0.0010 |
| Gtf2ird2 | -0.34 | 0.0491 |
| Gtf2i | 0.84 | 0.0000 |
| Rfc2 | 0.32 | 0.0752 |
| Lat2 | -0.59 | 0.0564 |
| Eln | 1.09 | 0.0000 |
| Wbscr27 | -0.52 | 0.0018 |
| Cldn4 | -2.77 | 0.0000 |
| Abhd11 | -1.15 | 0.0000 |
| Stx1a | -0.49 | 0.0645 |
| Vps37d | 0.86 | 0.0207 |
| Tbl2 | 0.73 | 0.0029 |
| Nsun5 | -0.29 | 0.0579 |
| Hip1 | 1.69 | 0.0000 |
| Por | -0.68 | 0.0000 |
| Ywhag | -0.78 | 0.0000 |
| Orai2 | 0.36 | 0.0040 |
| Sh2b2 | -1.18 | 0.0000 |
| Cux1 | 0.28 | 0.0353 |
| Ift22 | 0.46 | 0.0037 |
| Plod3 | -0.42 | 0.0002 |
| Ap1s1 | -0.30 | 0.0426 |
| Serpine1 | -4.11 | 0.0000 |
| Trip6 | -0.68 | 0.0001 |
| Slc12a9 | 0.62 | 0.0005 |
| Ephb4 | 0.59 | 0.0063 |
| Pop7 | -0.36 | 0.0542 |
| Fbxo24 | 1.48 | 0.0700 |
| Agfg2 | -0.51 | 0.0058 |
| Tsc22d4 | 0.35 | 0.0009 |
| Mcm7 | 0.33 | 0.0113 |
| Cnpy4 | 0.36 | 0.0815 |
| Lamtor4 | 0.53 | 0.0122 |
| BC037034 | -0.92 | 0.0006 |
| Gpc2 | 0.83 | 0.0021 |
| Zfp157 | 0.42 | 0.0474 |
| A430033K04Rik | 0.63 | 0.0068 |
| Pdgfa | -1.78 | 0.0000 |
| 3110082I17Rik | -0.58 | 0.0062 |
| Zfand2a | -0.99 | 0.0000 |
| Micall2 | -1.26 | 0.0000 |
| Elfn1 | 2.57 | 0.0333 |
| Mad1l1 | 0.58 | 0.0000 |
| Ftsj2 | 0.60 | 0.0023 |
| Snx8 | -0.45 | 0.0000 |
| Chst12 | 1.94 | 0.0000 |
| Lfng | 1.01 | 0.0000 |
| Ttyh3 | 0.52 | 0.0000 |
| Iqce | 1.03 | 0.0000 |
| Amz1 | 0.99 | 0.0018 |
| Radil | -1.64 | 0.0000 |
| Papolb | -1.60 | 0.0109 |
| Wipi2 | -0.68 | 0.0000 |
| Fbxl18 | 0.56 | 0.0022 |
| Actb | -0.48 | 0.0099 |
| Fscn1 | 0.77 | 0.0000 |
| E130309D02Rik | -0.28 | 0.0450 |
| Kdelr2 | -0.25 | 0.0696 |
| Daglb | 0.99 | 0.0000 |
| Cyth3 | -0.47 | 0.0113 |
| Eif2ak1 | 0.26 | 0.0496 |
| Aimp2 | -0.91 | 0.0000 |
| Pms2 | 0.42 | 0.0045 |
| Bri3 | -0.58 | 0.0034 |
| Baiap2l1 | -0.73 | 0.0965 |
| Trrap | 0.48 | 0.0249 |
| Smurf1 | -0.53 | 0.0008 |
| Arpc1b | -0.26 | 0.0545 |
| Bud31 | -0.33 | 0.0668 |
| Zkscan14 | 0.56 | 0.0013 |
| Zfp655 | -0.43 | 0.0259 |
| 1700001J03Rik | -2.03 | 0.0125 |
| Rpl21 | -0.57 | 0.0800 |
| Lnx2 | 0.71 | 0.0012 |
| Polr1d | -0.48 | 0.0127 |
| Flt3 | -0.93 | 0.0000 |
| Slc7a1 | -1.31 | 0.0000 |
| Uspl1 | -0.41 | 0.0030 |
| Alox5ap | 0.58 | 0.0564 |
| Hsph1 | -0.70 | 0.0000 |
| Fry | 0.90 | 0.0326 |
| Brca2 | 0.55 | 0.0032 |
| Pds5b | 0.44 | 0.0700 |
| Samd9l | -0.34 | 0.0671 |
| Bet1 | 0.33 | 0.0471 |
| Casd1 | 0.34 | 0.0169 |
| Asns | -1.05 | 0.0000 |
| Ndufa4 | 0.32 | 0.0374 |
| Thsd7a | 0.74 | 0.0060 |
| Tmem106b | 0.47 | 0.0023 |
| B630005N14Rik | 0.53 | 0.0051 |
| Tfec | -0.47 | 0.0007 |
| Cav2 | 0.67 | 0.0000 |
| Cav1 | 0.54 | 0.0000 |
| Cftr | -1.32 | 0.0000 |
| Tspan12 | 1.11 | 0.0000 |
| Cped1 | 1.38 | 0.0000 |
| Wasl | 0.36 | 0.0042 |
| Pot1a | 0.42 | 0.0141 |
| Gcc1 | 0.26 | 0.0392 |
| Lrrc4 | 1.40 | 0.0321 |
| Rbm28 | -0.36 | 0.0057 |
| Prrt4 | 1.55 | 0.0000 |
| Fam71f2 | -1.63 | 0.0249 |
| Smo | 0.31 | 0.0864 |
| Strip2 | 0.76 | 0.0852 |
| Nrf1 | -0.30 | 0.0426 |
| Ube2h | 0.27 | 0.0267 |
| Klhdc10 | 0.75 | 0.0000 |
| Tmem209 | 0.37 | 0.0743 |
| Cep41 | 0.50 | 0.0080 |
| Mest | 1.94 | 0.0000 |
| Copg2 | 0.64 | 0.0006 |
| Plxna4 | 1.03 | 0.0000 |
| Exoc4 | 0.48 | 0.0338 |
| Lrguk | 3.04 | 0.0006 |
| Slc35b4 | 0.84 | 0.0000 |
| Akr1b8 | -0.59 | 0.0009 |
| Bpgm | 1.44 | 0.0000 |
| Cald1 | 0.66 | 0.0213 |
| Agbl3 | 0.86 | 0.0000 |
| Tmem140 | 0.42 | 0.0690 |
| 3110062M04Rik | 0.37 | 0.0907 |
| Creb3l2 | 0.49 | 0.0038 |
| D630045J12Rik | 0.80 | 0.0023 |
| Zc3hav1l | 0.47 | 0.0136 |
| Zc3hav1 | -0.51 | 0.0070 |
| Ttc26 | 0.58 | 0.0167 |
| Ubn2 | 0.50 | 0.0006 |
| Hipk2 | 1.46 | 0.0000 |
| Slc37a3 | 0.29 | 0.0226 |
| Mkrn1 | -0.24 | 0.0989 |
| Adck2 | 0.39 | 0.0107 |
| E330009J07Rik | 1.53 | 0.0000 |
| Gstk1 | 1.11 | 0.0000 |
| Casp2 | 1.02 | 0.0000 |
| Fam131b | 2.38 | 0.0000 |
| Fam115c | 0.89 | 0.0009 |
| Arhgef5 | 0.47 | 0.0021 |
| Cul1 | -0.30 | 0.0099 |
| Ezh2 | 0.35 | 0.0282 |
| Zfp398 | 0.51 | 0.0104 |
| Krba1 | 0.89 | 0.0000 |
| Gimap8 | -0.51 | 0.0000 |
| Gimap6 | -0.75 | 0.0000 |
| Gimap1 | 0.40 | 0.0003 |
| Tra2a | -0.35 | 0.0634 |
| Ccdc126 | 0.82 | 0.0061 |
| Mpp6 | -1.11 | 0.0000 |
| Dfna5 | -2.26 | 0.0000 |
| Osbpl3 | 0.50 | 0.0022 |
| Cycs | -0.64 | 0.0012 |
| Hnrnpa2b1 | -0.25 | 0.0225 |
| Skap2 | 0.37 | 0.0009 |
| Hoxa3 | 2.10 | 0.0005 |
| Hoxa5 | 2.62 | 0.0000 |
| Hoxa6 | 2.44 | 0.0035 |
| Tax1bp1 | -0.26 | 0.0423 |
| Jazf1 | -0.96 | 0.0000 |
| Creb5 | -0.50 | 0.0001 |
| Tril | 3.56 | 0.0004 |
| Chn2 | 1.98 | 0.0000 |
| Scrn1 | 2.11 | 0.0000 |
| Fkbp14 | 0.96 | 0.0000 |
| Znrf2 | -0.49 | 0.0031 |
| Ggct | -0.47 | 0.0420 |
| Gars | -1.13 | 0.0000 |
| Fam188b | 1.37 | 0.0000 |
| Aqp1 | 1.62 | 0.0000 |
| Ppm1k | 1.13 | 0.0000 |
| Herc6 | -0.65 | 0.0042 |
| Herc3 | 0.81 | 0.0000 |
| Gprin3 | 3.48 | 0.0000 |
| Prdm5 | -0.71 | 0.0019 |
| Mad2l1 | 0.24 | 0.0847 |
| Gadd45a | -2.04 | 0.0000 |
| Serbp1 | -0.25 | 0.0134 |
| Rpia | -0.63 | 0.0000 |
| Reep1 | 0.99 | 0.0233 |
| Immt | -0.46 | 0.0000 |
| Ptcd3 | -0.32 | 0.0203 |
| Polr1a | -0.62 | 0.0016 |
| St3gal5 | 0.96 | 0.0000 |
| Atoh8 | 2.87 | 0.0000 |
| Usp39 | -0.23 | 0.0623 |
| Ggcx | 0.90 | 0.0000 |
| Mat2a | -0.51 | 0.0320 |
| Tcf7l1 | 0.89 | 0.0000 |
| Kcmf1 | -0.43 | 0.0003 |
| Mrpl19 | -0.38 | 0.0224 |
| Eva1a | -0.55 | 0.0020 |
| Pole4 | -0.57 | 0.0070 |
| Hk2 | -2.43 | 0.0000 |
| Htra2 | -0.39 | 0.0517 |
| Mogs | -0.43 | 0.0045 |
| Wbp1 | 0.68 | 0.0007 |
| Rtkn | 0.32 | 0.0212 |
| Wdr54 | 0.61 | 0.0723 |
| 1700003E16Rik | 1.68 | 0.0369 |
| Mthfd2 | -1.21 | 0.0000 |
| Tex261 | 0.61 | 0.0062 |
| Paip2b | 0.69 | 0.0002 |
| Spr | 0.47 | 0.0002 |
| Rab11fip5 | -0.40 | 0.0524 |
| Smyd5 | -0.95 | 0.0000 |
| Cct7 | -0.45 | 0.0001 |
| Alms1 | 0.86 | 0.0002 |
| 1700019G17Rik | 0.99 | 0.0469 |
| Cml1 | 1.36 | 0.0286 |
| Dusp11 | -0.35 | 0.0036 |
| Add2 | 0.83 | 0.0001 |
| Tgfa | -0.56 | 0.0026 |
| Fam136a | -0.71 | 0.0000 |
| Pcyox1 | 0.82 | 0.0000 |
| Pcbp1 | -0.36 | 0.0023 |
| Mxd1 | 1.06 | 0.0000 |
| Gmcl1 | 0.46 | 0.0006 |
| Anxa4 | -0.25 | 0.0162 |
| Antxr1 | 2.22 | 0.0000 |
| Gkn1 | 2.29 | 0.0017 |
| Prokr1 | 4.25 | 0.0000 |
| Aplf | 1.56 | 0.0000 |
| Rab43 | 0.97 | 0.0022 |
| Cnbp | -0.23 | 0.0989 |
| H1fx | 0.68 | 0.0983 |
| Kbtbd12 | 1.74 | 0.0038 |
| Mgll | 1.53 | 0.0000 |
| Mcm2 | 0.55 | 0.0023 |
| Tpra1 | 0.37 | 0.0520 |
| Txnrd3 | -0.39 | 0.0533 |
| Slc41a3 | 0.98 | 0.0001 |
| Iqsec1 | 1.02 | 0.0000 |
| Nup210 | 1.12 | 0.0000 |
| Hdac11 | 2.75 | 0.0193 |
| Fbln2 | 0.58 | 0.0042 |
| Chchd4 | -0.66 | 0.0000 |
| Xpc | 0.28 | 0.0511 |
| Slc6a6 | 0.37 | 0.0674 |
| Fgd5 | 0.85 | 0.0000 |
| Slc25a26 | -0.39 | 0.0651 |
| Eogt | 1.18 | 0.0000 |
| Frmd4b | 0.39 | 0.0335 |
| Foxp1 | 1.11 | 0.0000 |
| Shq1 | -0.79 | 0.0000 |
| Ppp4r2 | -0.88 | 0.0000 |
| Trnt1 | -0.46 | 0.0011 |
| Sumf1 | 0.50 | 0.0119 |
| Itpr1 | 0.93 | 0.0052 |
| Bhlhe40 | -1.46 | 0.0000 |
| Arl8b | 0.44 | 0.0026 |
| Lmcd1 | 2.18 | 0.0000 |
| Srgap3 | -1.07 | 0.0179 |
| Thumpd3 | -0.28 | 0.0269 |
| Setd5 | -0.24 | 0.0572 |
| Lhfpl4 | 0.47 | 0.0002 |
| Mtmr14 | -0.43 | 0.0028 |
| Brpf1 | -0.46 | 0.0000 |
| Ttll3 | -0.85 | 0.0004 |
| Jagn1 | -0.22 | 0.0902 |
| Fancd2 | 0.62 | 0.0168 |
| Vhl | 0.42 | 0.0304 |
| Irak2 | -1.17 | 0.0000 |
| Tatdn2 | -0.58 | 0.0011 |
| Vgll4 | 1.21 | 0.0000 |
| Syn2 | 0.97 | 0.0000 |
| Pparg | 0.68 | 0.0000 |
| Tsen2 | 0.44 | 0.0224 |
| Cand2 | 1.05 | 0.0000 |
| Mbd4 | 1.15 | 0.0000 |
| Ift122 | 0.68 | 0.0000 |
| H1foo | -1.68 | 0.0082 |
| Zfand4 | 1.39 | 0.0000 |
| Alox5 | -2.45 | 0.0314 |
| Cxcl12 | -0.58 | 0.0056 |
| Fxyd4 | -1.31 | 0.0317 |
| Csgalnact2 | -0.32 | 0.0323 |
| Bms1 | -0.34 | 0.0334 |
| Zfp248 | -0.34 | 0.0514 |
| Ankrd26 | 0.66 | 0.0059 |
| Cacna2d4 | 1.06 | 0.0002 |
| Fbxl14 | 0.33 | 0.0075 |
| Rad52 | -0.97 | 0.0000 |
| Il17ra | -0.84 | 0.0000 |
| Cecr5 | 0.30 | 0.0778 |
| Bid | -1.27 | 0.0000 |
| Pex26 | 0.68 | 0.0001 |
| C1rl | 1.42 | 0.0133 |
| C1ra | -0.76 | 0.0859 |
| Emg1 | -0.58 | 0.0058 |
| Phb2 | -0.48 | 0.0008 |
| Grcc10 | 0.29 | 0.0440 |
| Gpr162 | 0.97 | 0.0000 |
| Lag3 | -0.56 | 0.0827 |
| Cops7a | -0.20 | 0.0900 |
| Nop2 | -1.15 | 0.0000 |
| Gapdh | -0.72 | 0.0075 |
| Ncapd2 | 0.69 | 0.0002 |
| Vamp1 | 1.06 | 0.0000 |
| Cd27 | 1.10 | 0.0112 |
| D6Wsu163e | 0.57 | 0.0005 |
| Cracr2a | 2.23 | 0.0000 |
| Tead4 | -0.73 | 0.0000 |
| Tulp3 | 0.32 | 0.0326 |
| Itfg2 | -0.28 | 0.0397 |
| Clec1b | -0.99 | 0.0025 |
| Clec1a | 0.44 | 0.0420 |
| Klra4 | -0.65 | 0.0604 |
| Klra2 | -2.00 | 0.0000 |
| Magohb | -0.83 | 0.0015 |
| Styk1 | -0.55 | 0.0812 |
| Ybx3 | -0.97 | 0.0000 |
| Mansc1 | 1.25 | 0.0000 |
| Loh12cr1 | 0.53 | 0.0630 |
| Dusp16 | -0.57 | 0.0017 |
| Crebl2 | 0.72 | 0.0001 |
| Apold1 | -0.55 | 0.0000 |
| Gprc5a | -0.83 | 0.0000 |
| Gprc5d | -1.87 | 0.0478 |
| 8430419L09Rik | 0.44 | 0.0338 |
| Atf7ip | 1.02 | 0.0000 |
| Rerg | 1.25 | 0.0000 |
| Strap | -0.31 | 0.0074 |
| Aebp2 | -0.31 | 0.0503 |
| Recql | 0.56 | 0.0070 |
| Golt1b | -0.29 | 0.0677 |
| Cmas | -0.28 | 0.0299 |
| C2cd5 | 0.48 | 0.0247 |
| Bcat1 | -1.64 | 0.0000 |
| Kras | -0.36 | 0.0214 |
| Rassf8 | 0.99 | 0.0000 |
| Bhlhe41 | 0.47 | 0.0018 |
| Sspn | 0.55 | 0.0947 |
| Asun | -0.42 | 0.0010 |
| Tm7sf3 | 1.04 | 0.0000 |
| Med21 | -0.39 | 0.0789 |
| Stk38l | -1.10 | 0.0000 |
| Arntl2 | -0.95 | 0.0000 |
| Far2 | -0.89 | 0.0064 |
| Ergic2 | -0.20 | 0.0945 |
| Ipo8 | 0.38 | 0.0595 |
| Caprin2 | 1.47 | 0.0044 |
| Amn1 | -0.51 | 0.0072 |
| 2810474O19Rik | -0.79 | 0.0000 |
| Bicd1 | 0.97 | 0.0084 |
| Prkcg | -1.68 | 0.0000 |
| Cacng8 | 2.39 | 0.0028 |
| Prpf31 | -0.40 | 0.0124 |
| Leng1 | -0.55 | 0.0121 |
| Tsen34 | 0.42 | 0.0013 |
| Lair1 | 1.66 | 0.0599 |
| Ttyh1 | 0.67 | 0.0000 |
| Leng8 | -0.48 | 0.0027 |
| Cdc42ep5 | 0.87 | 0.0002 |
| Rdh13 | -0.29 | 0.0942 |
| Dnaaf3 | -1.58 | 0.0000 |
| Hspbp1 | -0.49 | 0.0347 |
| Il11 | -1.24 | 0.0215 |
| Rpl28 | -0.81 | 0.0159 |
| Ube2s | -0.52 | 0.0118 |
| Isoc2b | 1.30 | 0.0000 |
| Isoc2a | 0.39 | 0.0503 |
| Nat14 | -0.48 | 0.0136 |
| Ssc5d | -0.65 | 0.0450 |
| U2af2 | -0.49 | 0.0008 |
| Zfp78 | 0.66 | 0.0276 |
| Zfp28 | -0.42 | 0.0502 |
| Zfp954 | -0.37 | 0.0618 |
| Zfp418 | 0.71 | 0.0555 |
| Zik1 | 0.55 | 0.0516 |
| Zfp551 | 1.13 | 0.0018 |
| Zfp329 | 0.33 | 0.0257 |
| Zfp128 | 0.69 | 0.0017 |
| 2310014L17Rik | -1.67 | 0.0000 |
| Trim28 | -0.37 | 0.0019 |
| Ube2m | -0.35 | 0.0505 |
| Ehd2 | 0.63 | 0.0001 |
| Gltscr1 | 0.43 | 0.0007 |
| Napa | -0.47 | 0.0002 |
| Inafm1 | -0.73 | 0.0000 |
| Ccdc9 | -0.50 | 0.0001 |
| Arhgap35 | 0.42 | 0.0513 |
| Slc1a5 | -1.69 | 0.0000 |
| Fkrp | -0.28 | 0.0241 |
| Strn4 | -0.67 | 0.0000 |
| Ptgir | 1.55 | 0.0028 |
| Calm3 | 0.42 | 0.0002 |
| Nova2 | 0.66 | 0.0001 |
| Rsph6a | -1.11 | 0.0121 |
| Six5 | 0.84 | 0.0000 |
| Gipr | -1.31 | 0.0015 |
| Eml2 | 0.25 | 0.0874 |
| Opa3 | -0.47 | 0.0004 |
| Vasp | -0.30 | 0.0263 |
| Fosb | -1.15 | 0.0000 |
| Ercc1 | -0.74 | 0.0000 |
| Cd3eap | -0.54 | 0.0098 |
| Ppp1r13l | -0.93 | 0.0000 |
| Mark4 | 0.28 | 0.0252 |
| Exoc3l2 | 1.97 | 0.0000 |
| Nkpd1 | -0.81 | 0.0221 |
| Relb | -2.08 | 0.0000 |
| Bcl3 | -1.94 | 0.0000 |
| Pvr | -2.37 | 0.0000 |
| Zfp108 | 0.70 | 0.0166 |
| Zfp93 | 0.54 | 0.0286 |
| Kcnn4 | 1.49 | 0.0001 |
| Smg9 | -0.49 | 0.0059 |
| Plaur | -1.59 | 0.0000 |
| Ceacam10 | 1.24 | 0.0372 |
| Dedd2 | 0.34 | 0.0184 |
| Zfp526 | 0.80 | 0.0329 |
| Lipe | 0.64 | 0.0130 |
| Ceacam1 | 0.62 | 0.0000 |
| Ceacam2 | 0.67 | 0.0118 |
| Bckdha | 0.39 | 0.0315 |
| B9d2 | -0.36 | 0.0313 |
| Tgfb1 | -0.51 | 0.0000 |
| Hnrnpul1 | 0.25 | 0.0333 |
| Cyp2t4 | -0.87 | 0.0149 |
| BC024978 | -0.81 | 0.0001 |
| Itpkc | -0.90 | 0.0007 |
| Adck4 | -0.34 | 0.0252 |
| Sertad1 | -1.00 | 0.0000 |
| Hipk4 | -0.60 | 0.0616 |
| 2310022A10Rik | -0.66 | 0.0000 |
| Psmc4 | -0.48 | 0.0016 |
| Fbl | -0.82 | 0.0000 |
| Dyrk1b | 0.78 | 0.0000 |
| Eid2 | -0.54 | 0.0525 |
| Eid2b | 0.59 | 0.0021 |
| Timm50 | -0.39 | 0.0119 |
| Supt5 | -0.25 | 0.0746 |
| Plekhg2 | -0.58 | 0.0005 |
| Zfp36 | -1.85 | 0.0000 |
| Paf1 | -0.29 | 0.0340 |
| Lrfn1 | 1.67 | 0.0915 |
| Nfkbib | -1.23 | 0.0000 |
| Ech1 | 0.41 | 0.0328 |
| Capn12 | -1.22 | 0.0309 |
| Map4k1 | 1.12 | 0.0015 |
| Spred3 | -0.73 | 0.0000 |
| Psmd8 | -0.35 | 0.0023 |
| Spint2 | -0.89 | 0.0000 |
| Ppp1r14a | -0.69 | 0.0102 |
| Zfp940 | 0.50 | 0.0777 |
| Zfp566 | -0.94 | 0.0120 |
| Zfp260 | 0.26 | 0.0464 |
| Zfp146 | -0.55 | 0.0005 |
| Gm5113 | 0.54 | 0.0120 |
| Syne4 | -0.65 | 0.0446 |
| Lrfn3 | 1.31 | 0.0119 |
| Arhgap33 | 0.42 | 0.0561 |
| Zbtb32 | -0.85 | 0.0849 |
| Upk1a | -1.13 | 0.0040 |
| Fxyd5 | -0.55 | 0.0161 |
| Lgi4 | 1.27 | 0.0000 |
| Hpn | 0.56 | 0.0124 |
| Pdcd2l | -0.51 | 0.0011 |
| Pepd | -0.24 | 0.0720 |
| Cebpg | -0.71 | 0.0000 |
| Rhpn2 | 0.41 | 0.0102 |
| Cep89 | 1.02 | 0.0000 |
| Nudt19 | 0.63 | 0.0034 |
| Rgs9bp | 2.04 | 0.0026 |
| Ankrd27 | -0.70 | 0.0000 |
| Dpy19l3 | 1.09 | 0.0000 |
| Ccne1 | 0.74 | 0.0000 |
| AI987944 | -0.53 | 0.0006 |
| Ctu1 | -0.45 | 0.0008 |
| Klk7 | -1.41 | 0.0428 |
| Fam71e1 | 0.61 | 0.0787 |
| Pold1 | 0.33 | 0.0774 |
| Nr1h2 | -0.30 | 0.0265 |
| Atf5 | -0.93 | 0.0000 |
| Pnkp | 0.29 | 0.0561 |
| Rras | -0.59 | 0.0040 |
| Prr12 | 0.42 | 0.0002 |
| Fcgrt | 1.46 | 0.0000 |
| Rpl13a | -0.51 | 0.0372 |
| Aldh16a1 | -0.54 | 0.0010 |
| Trpm4 | -0.65 | 0.0000 |
| Snrnp70 | -0.39 | 0.0257 |
| Ftl1 | -0.75 | 0.0008 |
| Bax | -0.56 | 0.0367 |
| Tulp2 | -2.51 | 0.0000 |
| Ppp1r15a | -2.23 | 0.0000 |
| Izumo1 | -1.93 | 0.0000 |
| Rasip1 | -0.92 | 0.0000 |
| Mamstr | -1.19 | 0.0000 |
| Fut2 | -1.89 | 0.0000 |
| Sec1 | -1.07 | 0.0219 |
| Ntn5 | -2.21 | 0.0000 |
| Fam83e | -1.98 | 0.0029 |
| Spaca4 | -4.49 | 0.0161 |
| Cyth2 | -0.41 | 0.0672 |
| Kcnj14 | -1.93 | 0.0004 |
| Abcc6 | 1.61 | 0.0006 |
| Gtf2h1 | -0.33 | 0.0019 |
| Tsg101 | -0.32 | 0.0272 |
| Spty2d1 | -0.75 | 0.0017 |
| Tmem86a | 1.41 | 0.0000 |
| Zdhhc13 | -0.24 | 0.0944 |
| Prmt3 | -0.87 | 0.0000 |
| Nipa1 | 0.80 | 0.0000 |
| Atp10a | -1.53 | 0.0000 |
| Mkrn3 | 0.85 | 0.0297 |
| Peg12 | 0.98 | 0.0014 |
| Mtmr10 | -0.33 | 0.0620 |
| Fan1 | 1.01 | 0.0004 |
| Mphosph10 | -1.05 | 0.0000 |
| Mcee | 0.72 | 0.0276 |
| Tjp1 | 0.66 | 0.0053 |
| Tarsl2 | 1.06 | 0.0000 |
| Pcsk6 | 0.56 | 0.0329 |
| Snrpa1 | -0.56 | 0.0027 |
| Chsy1 | -0.28 | 0.0310 |
| Aldh1a3 | -1.43 | 0.0000 |
| Lins | -0.60 | 0.0000 |
| Adamts17 | 0.47 | 0.0530 |
| Mef2a | -0.50 | 0.0066 |
| Lrrc28 | 0.55 | 0.0012 |
| Ttc23 | 0.67 | 0.0000 |
| Arrdc4 | -2.78 | 0.0000 |
| Rgma | -1.08 | 0.0000 |
| Chd2 | -0.72 | 0.0030 |
| Slco3a1 | -0.62 | 0.0000 |
| Akap13 | 0.69 | 0.0148 |
| Aen | -0.44 | 0.0001 |
| Isg20 | -0.82 | 0.0004 |
| Hapln3 | -1.23 | 0.0079 |
| Abhd2 | 0.64 | 0.0240 |
| Fanci | 0.87 | 0.0021 |
| Ap3s2 | 0.50 | 0.0000 |
| 2610034B18Rik | 0.42 | 0.0055 |
| Idh2 | 0.54 | 0.0128 |
| Sema4b | -1.67 | 0.0000 |
| Ttll13 | 0.69 | 0.0712 |
| Rccd1 | -0.51 | 0.0132 |
| Hddc3 | 0.67 | 0.0278 |
| Man2a2 | 0.76 | 0.0000 |
| Fes | -0.35 | 0.0419 |
| Wdr73 | -0.45 | 0.0120 |
| Sec11a | -0.34 | 0.0082 |
| Zfp592 | -0.27 | 0.0715 |
| Alpk3 | 0.81 | 0.0157 |
| Pde8a | 0.60 | 0.0006 |
| Cpeb1 | -0.74 | 0.0000 |
| Btbd1 | 0.28 | 0.0316 |
| Tm6sf1 | 1.22 | 0.0000 |
| Hdgfrp3 | 0.56 | 0.0020 |
| Fam154b | 2.74 | 0.0500 |
| Mex3b | 0.37 | 0.0926 |
| Il16 | 1.13 | 0.0877 |
| 1700026D08Rik | -1.50 | 0.0484 |
| Mesdc1 | -0.61 | 0.0000 |
| Cemip | 2.01 | 0.0000 |
| Abhd17c | -0.44 | 0.0020 |
| Arnt2 | 0.37 | 0.0836 |
| Fah | 0.81 | 0.0003 |
| Nox4 | -1.23 | 0.0000 |
| Prss23 | -3.73 | 0.0000 |
| Me3 | -2.01 | 0.0000 |
| Picalm | -0.55 | 0.0000 |
| Sytl2 | 1.24 | 0.0002 |
| Ccdc89 | 2.26 | 0.0012 |
| Tmem126b | 0.86 | 0.0000 |
| Pcf11 | -0.56 | 0.0000 |
| Ddias | 0.66 | 0.0009 |
| Prcp | 0.69 | 0.0000 |
| Gab2 | -0.62 | 0.0011 |
| Usp35 | 0.51 | 0.0059 |
| Thrsp | 2.10 | 0.0000 |
| Aamdc | 0.52 | 0.0224 |
| Aqp11 | 1.28 | 0.0000 |
| Pak1 | -0.54 | 0.0009 |
| Myo7a | 2.51 | 0.0000 |
| Capn5 | 1.60 | 0.0000 |
| Acer3 | 0.60 | 0.0000 |
| Lrrc32 | -0.45 | 0.0000 |
| Uvrag | 0.91 | 0.0000 |
| Gdpd5 | 1.17 | 0.0000 |
| Klhl35 | 0.67 | 0.0669 |
| Arrb1 | 1.67 | 0.0000 |
| Rnf169 | 0.41 | 0.0202 |
| Pold3 | -0.36 | 0.0463 |
| Pgm2l1 | 0.84 | 0.0001 |
| Ppme1 | 0.25 | 0.0891 |
| C2cd3 | 0.81 | 0.0044 |
| Rab6a | -0.56 | 0.0000 |
| Arhgef17 | -0.78 | 0.0162 |
| P2ry6 | -0.84 | 0.0012 |
| Fchsd2 | 0.60 | 0.0011 |
| Atg16l2 | -0.42 | 0.0756 |
| Folr2 | 1.93 | 0.0552 |
| Il18bp | -0.57 | 0.0008 |
| Pgap2 | 0.67 | 0.0009 |
| Stim1 | 1.15 | 0.0000 |
| Rrm1 | 0.74 | 0.0000 |
| Smpd1 | 0.62 | 0.0000 |
| Hpx | -1.11 | 0.0003 |
| Trim3 | -0.96 | 0.0000 |
| Arfip2 | -0.64 | 0.0082 |
| Timm10b | -0.50 | 0.0818 |
| Tub | -2.72 | 0.0000 |
| Lmo1 | 0.39 | 0.0372 |
| Rpl27a | -0.66 | 0.0078 |
| St5 | 0.27 | 0.0850 |
| Tmem41b | -1.25 | 0.0000 |
| Ipo7 | -0.68 | 0.0002 |
| Adm | -1.46 | 0.0000 |
| Galnt18 | 0.83 | 0.0000 |
| Mical2 | 0.95 | 0.0000 |
| Parva | 0.32 | 0.0302 |
| Tead1 | -0.59 | 0.0002 |
| Arntl | -0.59 | 0.0003 |
| Far1 | -0.41 | 0.0057 |
| Spon1 | -0.63 | 0.0494 |
| Rras2 | -0.48 | 0.0000 |
| Calcb | -0.82 | 0.0000 |
| Sox6 | 2.00 | 0.0000 |
| Plekha7 | 0.73 | 0.0000 |
| Rps13 | -0.97 | 0.0002 |
| Nucb2 | -0.40 | 0.0033 |
| Rps15a | -0.30 | 0.0890 |
| Arl6ip1 | 0.51 | 0.0026 |
| Syt17 | -2.03 | 0.0000 |
| Itpripl2 | -1.28 | 0.0000 |
| Tmc7 | 1.13 | 0.0000 |
| Gde1 | 0.28 | 0.0853 |
| Ccp110 | 0.81 | 0.0009 |
| Vwa3a | 0.90 | 0.0048 |
| Polr3e | -0.55 | 0.0002 |
| Cdr2 | -0.93 | 0.0000 |
| Usp31 | -0.45 | 0.0058 |
| Cog7 | 0.92 | 0.0000 |
| Gga2 | 0.79 | 0.0000 |
| Ubfd1 | 0.27 | 0.0840 |
| Dctn5 | 0.31 | 0.0153 |
| Plk1 | 0.29 | 0.0455 |
| Chp2 | 3.19 | 0.0000 |
| Rbbp6 | -0.44 | 0.0019 |
| Arhgap17 | 0.35 | 0.0450 |
| Lcmt1 | 0.53 | 0.0018 |
| Kdm8 | 0.53 | 0.0111 |
| Il21r | 2.40 | 0.0003 |
| D430042O09Rik | 0.25 | 0.0966 |
| Sbk1 | 1.07 | 0.0001 |
| Atp2a1 | -0.79 | 0.0038 |
| Sh2b1 | -0.66 | 0.0000 |
| Atxn2l | -0.83 | 0.0000 |
| Eif3c | -0.68 | 0.0000 |
| Apobr | -0.64 | 0.0006 |
| Nupr1 | -1.68 | 0.0000 |
| Sult1a1 | 1.49 | 0.0017 |
| Slx1b | 0.85 | 0.0000 |
| Bola2 | -0.63 | 0.0463 |
| Mapk3 | 0.39 | 0.0179 |
| Fam57b | 1.48 | 0.0000 |
| Taok2 | 0.38 | 0.0079 |
| Tmem219 | 0.36 | 0.0259 |
| Cdipt | 0.20 | 0.0751 |
| Mvp | -1.16 | 0.0000 |
| Maz | -0.32 | 0.0612 |
| AI467606 | 1.91 | 0.0081 |
| Tbc1d10b | -0.39 | 0.0066 |
| Itgal | 2.33 | 0.0000 |
| Zfp747 | -0.58 | 0.0001 |
| Fbrs | -0.33 | 0.0174 |
| Srcap | 0.50 | 0.0430 |
| Gm166 | 1.75 | 0.0000 |
| Orai3 | 0.72 | 0.0000 |
| Hsd3b7 | -1.72 | 0.0000 |
| Stx4a | -0.65 | 0.0112 |
| Bckdk | 0.38 | 0.0135 |
| Fus | -0.47 | 0.0002 |
| Pycard | -1.15 | 0.0481 |
| Itgax | -2.23 | 0.0000 |
| Itgad | -1.40 | 0.0941 |
| Armc5 | -0.48 | 0.0000 |
| Tgfb1i1 | -0.99 | 0.0000 |
| Tial1 | -0.47 | 0.0080 |
| Tacc2 | -0.34 | 0.0018 |
| Plekha1 | -0.56 | 0.0017 |
| 1700007K09Rik | -3.88 | 0.0115 |
| 2310057M21Rik | -0.58 | 0.0006 |
| Pstk | 0.32 | 0.0607 |
| Acadsb | 0.44 | 0.0165 |
| Oat | 0.30 | 0.0902 |
| Bccip | -0.31 | 0.0898 |
| Adam12 | 1.25 | 0.0000 |
| Mgmt | 0.42 | 0.0845 |
| 9430038I01Rik | -0.68 | 0.0016 |
| Ppp2r2d | -0.36 | 0.0044 |
| Lrrc27 | -1.11 | 0.0000 |
| Pwwp2b | 0.35 | 0.0250 |
| Adam8 | -1.63 | 0.0000 |
| Zfp511 | -0.65 | 0.0139 |
| Fuom | -0.71 | 0.0669 |
| Paox | 0.65 | 0.0018 |
| Mtg1 | 0.39 | 0.0314 |
| Urah | -4.40 | 0.0000 |
| Bet1l | -0.37 | 0.0564 |
| Ric8 | -0.74 | 0.0000 |
| Sirt3 | 0.37 | 0.0829 |
| Athl1 | 0.28 | 0.0703 |
| Ifitm5 | -0.99 | 0.0491 |
| Lrrc56 | 1.24 | 0.0000 |
| Lmntd2 | 1.42 | 0.0469 |
| Phrf1 | -0.37 | 0.0007 |
| Irf7 | -0.57 | 0.0010 |
| Eps8l2 | 1.85 | 0.0013 |
| Taldo1 | -0.97 | 0.0000 |
| Pddc1 | 0.40 | 0.0651 |
| Pnpla2 | -0.64 | 0.0000 |
| Cracr2b | 1.09 | 0.0000 |
| Chid1 | 0.35 | 0.0060 |
| Dusp8 | -0.85 | 0.0000 |
| Kcnq1 | 0.48 | 0.0005 |
| Cars | -1.51 | 0.0000 |
| Tnfrsf22 | -0.98 | 0.0000 |
| Tnfrsf23 | -0.49 | 0.0017 |
| Mrgpre | 0.96 | 0.0000 |
| Shank2 | -1.33 | 0.0589 |
| Fadd | 0.31 | 0.0950 |
| Ano1 | -1.14 | 0.0000 |
| Akap12 | -2.60 | 0.0000 |
| 1700052N19Rik | 0.55 | 0.0046 |
| Myct1 | 0.43 | 0.0078 |
| Katna1 | -0.47 | 0.0000 |
| Samd5 | 0.84 | 0.0000 |
| Stxbp5 | -0.31 | 0.0079 |
| Adgb | -2.12 | 0.0000 |
| Rab32 | -0.95 | 0.0000 |
| Grm1 | 1.36 | 0.0000 |
| Epm2a | 1.43 | 0.0000 |
| Plagl1 | 0.88 | 0.0002 |
| Ltv1 | -1.32 | 0.0000 |
| Phactr2 | 1.30 | 0.0000 |
| Pex3 | 0.33 | 0.0295 |
| Adat2 | 0.62 | 0.0325 |
| Aig1 | -0.66 | 0.0073 |
| Cited2 | -1.21 | 0.0000 |
| Txlnb | 0.85 | 0.0001 |
| Reps1 | -0.62 | 0.0000 |
| Nhsl1 | 0.62 | 0.0000 |
| Ifngr1 | -1.14 | 0.0000 |
| Mtfr2 | 0.53 | 0.0118 |
| Pde7b | 2.30 | 0.0000 |
| Ahi1 | 0.50 | 0.0395 |
| Hbs1l | -0.31 | 0.0082 |
| Sgk1 | 0.71 | 0.0000 |
| H60b | 0.70 | 0.0209 |
| Raet1d | 0.46 | 0.0060 |
| Tbpl1 | -0.22 | 0.0878 |
| Rps12 | -1.06 | 0.0000 |
| Slc18b1 | 1.69 | 0.0000 |
| Stx7 | -0.30 | 0.0035 |
| Ctgf | -1.39 | 0.0000 |
| Tmem200a | 1.06 | 0.0000 |
| Rspo3 | 0.96 | 0.0011 |
| Trmt11 | -0.46 | 0.0182 |
| Hint3 | 0.42 | 0.0121 |
| Ncoa7 | 0.78 | 0.0012 |
| Nkain2 | 0.68 | 0.0018 |
| Zufsp | -0.44 | 0.0007 |
| Fam26e | 0.41 | 0.0156 |
| Tspyl1 | 0.42 | 0.0366 |
| Tspyl4 | 2.03 | 0.0000 |
| Amd2 | -0.37 | 0.0322 |
| Tube1 | 0.77 | 0.0004 |
| Fyn | -0.31 | 0.0470 |
| Rpf2 | -1.08 | 0.0000 |
| Cdk19 | 0.90 | 0.0000 |
| Fig4 | 0.41 | 0.0017 |
| Mical1 | 0.41 | 0.0104 |
| Smpd2 | 0.35 | 0.0817 |
| Ppil6 | 2.82 | 0.0160 |
| Sesn1 | 0.81 | 0.0001 |
| Ostm1 | 0.70 | 0.0005 |
| Sobp | 0.93 | 0.0428 |
| Bend3 | -0.42 | 0.0514 |
| Qrsl1 | 0.35 | 0.0455 |
| Rtn4ip1 | 0.39 | 0.0188 |
| Prdm1 | -2.82 | 0.0004 |
| Prep | -0.59 | 0.0024 |
| Popdc3 | 0.84 | 0.0326 |
| Ascc3 | -0.62 | 0.0543 |
| Dcbld1 | 0.53 | 0.0190 |
| Gopc | 0.38 | 0.0863 |
| Nepn | 1.35 | 0.0000 |
| Mcm9 | 0.50 | 0.0216 |
| Tbc1d32 | 1.22 | 0.0000 |
| Msl3l2 | 1.11 | 0.0020 |
| Gja1 | -1.02 | 0.0000 |
| Smpdl3a | 0.79 | 0.0000 |
| Gcc2 | 0.71 | 0.0033 |
| Lims1 | 0.70 | 0.0012 |
| Oit3 | -0.94 | 0.0346 |
| Mcu | -0.43 | 0.0004 |
| Ddit4 | 0.29 | 0.0342 |
| Spock2 | 0.59 | 0.0512 |
| Chst3 | -0.86 | 0.0193 |
| 4632428N05Rik | 2.44 | 0.0000 |
| Slc29a3 | 0.83 | 0.0000 |
| Unc5b | 0.85 | 0.0040 |
| Pald1 | 1.01 | 0.0000 |
| Eif4ebp2 | 0.47 | 0.0195 |
| Ppa1 | -0.35 | 0.0058 |
| Aifm2 | 0.59 | 0.0004 |
| H2afy2 | 1.21 | 0.0000 |
| 2510003E04Rik | 0.28 | 0.0193 |
| Ddx21 | -0.72 | 0.0008 |
| Stox1 | 2.10 | 0.0000 |
| Ccar1 | -0.32 | 0.0023 |
| Slc25a16 | 0.90 | 0.0000 |
| Dna2 | 0.80 | 0.0006 |
| Rufy2 | 0.25 | 0.0722 |
| Sirt1 | -0.30 | 0.0274 |
| Dnajc12 | 1.70 | 0.0005 |
| Jmjd1c | -0.68 | 0.0028 |
| Zfp365 | 1.19 | 0.0000 |
| Rtkn2 | 2.21 | 0.0122 |
| Ank3 | 0.85 | 0.0157 |
| Slc16a9 | 2.80 | 0.0048 |
| Bicc1 | 0.68 | 0.0949 |
| Tfam | -0.41 | 0.0003 |
| Ube2d1 | -0.41 | 0.0017 |
| Ipmk | -0.85 | 0.0000 |
| Adora2a | 1.72 | 0.0000 |
| Snrpd3 | -0.30 | 0.0405 |
| Susd2 | -1.02 | 0.0001 |
| Ddt | 0.43 | 0.0183 |
| Gstt3 | 1.38 | 0.0000 |
| Gstt2 | 0.76 | 0.0000 |
| Mif | -0.52 | 0.0184 |
| Zfp280b | -0.40 | 0.0289 |
| Prmt2 | -0.26 | 0.0315 |
| S100b | 0.87 | 0.0481 |
| Dip2a | 0.45 | 0.0121 |
| Pcnt | 0.40 | 0.0110 |
| Ybey | 0.48 | 0.0331 |
| Lss | -5.87 | 0.0000 |
| Spatc1l | -6.74 | 0.0000 |
| Pcbp3 | -0.40 | 0.0008 |
| Col18a1 | -0.26 | 0.0715 |
| Sumo3 | 0.63 | 0.0000 |
| 1810043G02Rik | 0.50 | 0.0027 |
| Icosl | -1.88 | 0.0000 |
| D10Jhu81e | 0.45 | 0.0100 |
| Pwp2 | -1.16 | 0.0000 |
| Cstb | -0.35 | 0.0161 |
| Pdxk | 1.29 | 0.0552 |
| Syde1 | -0.68 | 0.0000 |
| 2610008E11Rik | 0.57 | 0.0000 |
| Madcam1 | -3.87 | 0.0000 |
| Gzmm | -0.96 | 0.0046 |
| Polrmt | -0.37 | 0.0117 |
| Fstl3 | 1.87 | 0.0640 |
| Palm | 1.55 | 0.0000 |
| Med16 | 0.24 | 0.0552 |
| R3hdm4 | 0.36 | 0.0143 |
| Kiss1r | 1.63 | 0.0000 |
| Arid3a | 0.46 | 0.0020 |
| Wdr18 | -0.44 | 0.0002 |
| Grin3b | -0.69 | 0.0074 |
| Tmem259 | -0.45 | 0.0241 |
| Sbno2 | -1.66 | 0.0000 |
| Stk11 | -0.48 | 0.0227 |
| Midn | -0.33 | 0.0505 |
| 1600002K03Rik | -0.69 | 0.0614 |
| Mum1 | -0.38 | 0.0990 |
| Gamt | 1.21 | 0.0000 |
| Adamtsl5 | -1.08 | 0.0000 |
| Mex3d | -1.08 | 0.0000 |
| Atp8b3 | -0.93 | 0.0117 |
| Klf16 | -0.51 | 0.0000 |
| Abhd17a | 0.96 | 0.0000 |
| Adat3 | -0.75 | 0.0056 |
| Btbd2 | 0.57 | 0.0001 |
| Mknk2 | 0.83 | 0.0000 |
| Mob3a | -0.66 | 0.0338 |
| Ap3d1 | -0.30 | 0.0037 |
| Plekhj1 | -0.58 | 0.0160 |
| Sf3a2 | -0.66 | 0.0007 |
| Amh | -0.89 | 0.0044 |
| Jsrp1 | -1.20 | 0.0074 |
| Lsm7 | -0.78 | 0.0231 |
| Gadd45b | -2.60 | 0.0000 |
| Creb3l3 | 1.35 | 0.0000 |
| Zbtb7a | -0.25 | 0.0344 |
| Pias4 | -0.40 | 0.0016 |
| Mrpl54 | -0.58 | 0.0449 |
| Apba3 | -0.68 | 0.0008 |
| Gipc3 | 2.45 | 0.0009 |
| Fzr1 | 0.39 | 0.0009 |
| Dohh | -0.62 | 0.0124 |
| Smim24 | -1.27 | 0.0114 |
| Nfic | 0.44 | 0.0040 |
| Celf5 | 2.47 | 0.0000 |
| Ncln | -0.52 | 0.0000 |
| S1pr4 | 1.10 | 0.0001 |
| Gna11 | 0.70 | 0.0000 |
| Tle2 | 1.78 | 0.0000 |
| Tle6 | 1.96 | 0.0314 |
| Zfp873 | 0.63 | 0.0013 |
| Tdg | -0.53 | 0.0000 |
| Nfyb | -0.76 | 0.0000 |
| Txnrd1 | -0.60 | 0.0035 |
| Slc41a2 | -0.79 | 0.0003 |
| A230046K03Rik | -0.29 | 0.0825 |
| Appl2 | 0.69 | 0.0000 |
| Nuak1 | 2.12 | 0.0000 |
| Tcp11l2 | 0.91 | 0.0000 |
| Polr3b | 0.45 | 0.0439 |
| Rfx4 | 3.29 | 0.0000 |
| Ric8b | 0.26 | 0.0628 |
| Fhl4 | 0.83 | 0.0719 |
| Tmem263 | 0.49 | 0.0003 |
| Mterf2 | 0.66 | 0.0127 |
| Cry1 | -0.39 | 0.0157 |
| Pwp1 | -0.71 | 0.0006 |
| Prdm4 | -0.37 | 0.0449 |
| Fbxo7 | 0.50 | 0.0000 |
| Syn3 | 1.14 | 0.0037 |
| Timp3 | 2.10 | 0.0000 |
| Nt5dc3 | -0.48 | 0.0237 |
| Stab2 | 1.15 | 0.0003 |
| Igf1 | 2.63 | 0.0000 |
| Parpbp | 0.84 | 0.0000 |
| Dram1 | -0.41 | 0.0938 |
| Gnptab | 0.48 | 0.0059 |
| Chpt1 | 0.99 | 0.0000 |
| Utp20 | -0.82 | 0.0000 |
| Uhrf1bp1l | -0.38 | 0.0181 |
| Ikbip | 0.57 | 0.0002 |
| Slc25a3 | -0.34 | 0.0022 |
| Nedd1 | -0.40 | 0.0127 |
| Cdk17 | -0.47 | 0.0010 |
| Elk3 | 0.70 | 0.0000 |
| Lta4h | 0.53 | 0.0000 |
| Ccdc38 | -1.51 | 0.0181 |
| Ntn4 | 0.81 | 0.0003 |
| Usp44 | -1.59 | 0.0053 |
| Fgd6 | 0.94 | 0.0000 |
| Cep83 | -0.50 | 0.0001 |
| Cradd | 0.70 | 0.0207 |
| Socs2 | -0.50 | 0.0002 |
| Ube2n | -0.43 | 0.0167 |
| Nudt4 | 0.41 | 0.0023 |
| Btg1 | 0.42 | 0.0000 |
| Poc1b | 0.70 | 0.0000 |
| Galnt4 | 0.84 | 0.0007 |
| Kitl | -0.48 | 0.0062 |
| Cep290 | 0.64 | 0.0121 |
| Nts | -0.56 | 0.0452 |
| Rassf9 | 1.38 | 0.0000 |
| Tmtc2 | 0.84 | 0.0001 |
| Ccdc59 | -0.49 | 0.0013 |
| E2f7 | 0.43 | 0.0306 |
| Bbs10 | 0.31 | 0.0955 |
| Phlda1 | -2.27 | 0.0000 |
| Krr1 | -0.43 | 0.0002 |
| Tmem19 | 0.83 | 0.0000 |
| Ptprb | 0.87 | 0.0030 |
| Best3 | 1.40 | 0.0153 |
| Cct2 | -0.61 | 0.0000 |
| Yeats4 | -0.50 | 0.0171 |
| Mdm2 | -0.59 | 0.0000 |
| Slc35e3 | -0.41 | 0.0034 |
| Nup107 | -0.28 | 0.0899 |
| Rap1b | -1.22 | 0.0000 |
| Mdm1 | 0.60 | 0.0015 |
| Dyrk2 | 0.34 | 0.0583 |
| Helb | 0.95 | 0.0000 |
| Irak3 | 1.52 | 0.0937 |
| Llph | -0.89 | 0.0003 |
| Hmga2 | -1.17 | 0.0000 |
| Msrb3 | 0.59 | 0.0027 |
| Lemd3 | -0.43 | 0.0207 |
| Tbc1d30 | 1.18 | 0.0008 |
| Rassf3 | 0.27 | 0.0239 |
| Tbk1 | -0.26 | 0.0465 |
| Xpot | -0.87 | 0.0000 |
| BC048403 | -0.60 | 0.0000 |
| Srgap1 | -1.15 | 0.0000 |
| Tmem5 | -0.33 | 0.0183 |
| Ctdsp2 | 0.70 | 0.0000 |
| Mettl1 | -1.22 | 0.0000 |
| Cyp27b1 | -2.09 | 0.0000 |
| Cdk4 | -0.29 | 0.0849 |
| Tspan31 | 0.25 | 0.0836 |
| B4galnt1 | 1.28 | 0.0012 |
| Pip4k2c | 0.49 | 0.0013 |
| Ddit3 | -1.11 | 0.0000 |
| Mars | -1.01 | 0.0000 |
| Shmt2 | -1.35 | 0.0000 |
| Ptges3 | -0.91 | 0.0000 |
| Baz2a | 0.42 | 0.0077 |
| Rbms2 | 0.80 | 0.0000 |
| Stat2 | -1.00 | 0.0000 |
| Il23a | -2.15 | 0.0182 |
| Pan2 | -0.33 | 0.0650 |
| Myl6 | -0.60 | 0.0001 |
| Myl6b | 0.46 | 0.0693 |
| Zc3h10 | -0.42 | 0.0005 |
| Pa2g4 | -0.55 | 0.0000 |
| Suox | 1.25 | 0.0000 |
| Pmel | 0.76 | 0.0900 |
| Dgka | 0.85 | 0.0000 |
| Gdf11 | 0.61 | 0.0000 |
| Rdh5 | -0.82 | 0.0003 |
| Itga7 | -1.60 | 0.0000 |
| Pex11g | 0.87 | 0.0010 |
| Zfp358 | 0.48 | 0.0084 |
| Camsap3 | 1.18 | 0.0819 |
| Xab2 | -0.29 | 0.0777 |
| Evi5l | 0.64 | 0.0000 |
| Map2k7 | -0.39 | 0.0305 |
| Snapc2 | -0.39 | 0.0668 |
| Timm44 | -0.36 | 0.0390 |
| Elavl1 | -0.41 | 0.0026 |
| Zfp958 | 0.45 | 0.0814 |
| Col4a1 | -0.39 | 0.0990 |
| Col4a2 | -0.40 | 0.0538 |
| Rab20 | -0.63 | 0.0706 |
| Cars2 | 0.31 | 0.0673 |
| Mcf2l | 3.28 | 0.0000 |
| Tmco3 | 0.48 | 0.0140 |
| Tfdp1 | -0.31 | 0.0215 |
| Gas6 | 0.78 | 0.0000 |
| 1700029H14Rik | -3.65 | 0.0205 |
| Fbxo25 | 0.50 | 0.0101 |
| Cln8 | -0.47 | 0.0040 |
| Arhgef10 | 0.59 | 0.0107 |
| Angpt2 | -1.52 | 0.0000 |
| Agpat5 | -0.41 | 0.0092 |
| Xkr5 | 1.04 | 0.0000 |
| Alg11 | -0.41 | 0.0113 |
| Ckap2 | 0.87 | 0.0000 |
| Vps36 | 0.27 | 0.0281 |
| Thsd1 | 0.78 | 0.0000 |
| Slc25a15 | 0.44 | 0.0802 |
| Vdac3 | -0.33 | 0.0962 |
| Ikbkb | -0.25 | 0.0595 |
| Ap3m2 | 0.74 | 0.0000 |
| 1810011O10Rik | -1.05 | 0.0000 |
| Tm2d2 | 0.30 | 0.0976 |
| Fgfr1 | 0.38 | 0.0007 |
| Whsc1l1 | 0.32 | 0.0824 |
| Bag4 | 0.69 | 0.0033 |
| Hgsnat | -0.27 | 0.0772 |
| Pomk | 0.85 | 0.0001 |
| Fnta | 0.22 | 0.0774 |
| Hook3 | 0.39 | 0.0179 |
| Thap1 | -0.40 | 0.0797 |
| Zfp703 | 0.39 | 0.0002 |
| Gpr124 | 0.43 | 0.0283 |
| Eif4ebp1 | -1.19 | 0.0000 |
| Tti2 | -0.51 | 0.0013 |
| Mak16 | -0.58 | 0.0004 |
| Fut10 | 1.54 | 0.0000 |
| Nrg1 | -1.54 | 0.0000 |
| Purg | 0.55 | 0.0538 |
| Ppp2cb | -0.26 | 0.0828 |
| Rbpms | -0.44 | 0.0052 |
| Leprotl1 | -0.39 | 0.0146 |
| Tnks | 0.65 | 0.0429 |
| Ppp1r3b | 2.34 | 0.0000 |
| Mfhas1 | -0.95 | 0.0000 |
| Cldn23 | -2.24 | 0.0010 |
| D8Ertd82e | -1.43 | 0.0000 |
| Lonrf1 | -1.41 | 0.0000 |
| AI429214 | 0.89 | 0.0033 |
| Tusc3 | 0.39 | 0.0355 |
| Cnot7 | -0.51 | 0.0018 |
| Asah1 | -0.43 | 0.0005 |
| Fam149a | 0.60 | 0.0039 |
| Tlr3 | 0.52 | 0.0021 |
| Sorbs2 | 0.26 | 0.0602 |
| Lrp2bp | 2.06 | 0.0005 |
| Acsl1 | 1.07 | 0.0000 |
| Casp3 | -0.78 | 0.0000 |
| Irf2 | -0.72 | 0.0000 |
| Enpp6 | 2.07 | 0.0000 |
| Stox2 | 0.81 | 0.0001 |
| Trappc11 | 0.46 | 0.0096 |
| Dctd | -1.06 | 0.0000 |
| Aga | 0.68 | 0.0000 |
| Cep44 | 0.36 | 0.0339 |
| Fbxo8 | 0.80 | 0.0000 |
| Galnt7 | 0.73 | 0.0045 |
| Tll1 | 2.01 | 0.0000 |
| Msmo1 | -4.31 | 0.0000 |
| Tma16 | -1.09 | 0.0000 |
| Ints10 | 0.66 | 0.0028 |
| D130040H23Rik | 1.01 | 0.0289 |
| Zfp868 | -0.70 | 0.0031 |
| Zfp964 | 0.90 | 0.0209 |
| Zfp869 | -0.31 | 0.0188 |
| Atp13a1 | -0.51 | 0.0003 |
| Gmip | -0.42 | 0.0338 |
| Lpar2 | -1.12 | 0.0017 |
| Cilp2 | -3.52 | 0.0000 |
| Sugp1 | -0.28 | 0.0964 |
| Nr2c2ap | -1.14 | 0.0006 |
| 2310045N01Rik | 0.33 | 0.0446 |
| Slc25a42 | 1.19 | 0.0000 |
| Armc6 | -0.41 | 0.0142 |
| Ddx49 | -0.35 | 0.0273 |
| Crtc1 | 0.56 | 0.0001 |
| Crlf1 | 1.23 | 0.0497 |
| Uba52 | -0.58 | 0.0229 |
| Isyna1 | -0.74 | 0.0562 |
| Lrrc25 | 0.80 | 0.0088 |
| Gdf15 | -3.87 | 0.0000 |
| Pgpep1 | 0.23 | 0.0610 |
| Jund | -0.84 | 0.0000 |
| Pik3r2 | 0.73 | 0.0000 |
| Mast3 | 0.46 | 0.0051 |
| Il12rb1 | -2.42 | 0.0057 |
| Arrdc2 | -1.63 | 0.0000 |
| Ccdc124 | -0.41 | 0.0764 |
| Slc5a5 | -1.29 | 0.0001 |
| Map1s | -0.42 | 0.0003 |
| Myo9b | -0.39 | 0.0209 |
| Use1 | -0.50 | 0.0445 |
| Abhd8 | 1.00 | 0.0000 |
| Dda1 | -0.21 | 0.0630 |
| Tmem221 | 1.58 | 0.0000 |
| Nxnl1 | 1.88 | 0.0101 |
| Slc27a1 | 0.35 | 0.0858 |
| Fam129c | -0.89 | 0.0004 |
| Unc13a | -0.60 | 0.0017 |
| B3gnt3 | 0.40 | 0.0757 |
| Zfp882 | 0.77 | 0.0111 |
| Zfp961 | 0.58 | 0.0118 |
| Ap1m1 | -0.33 | 0.0819 |
| Gm10282 | 0.52 | 0.0011 |
| Klf2 | -1.07 | 0.0000 |
| Slc35e1 | 0.60 | 0.0038 |
| Tmem38a | 0.56 | 0.0055 |
| Nwd1 | 2.66 | 0.0000 |
| Sin3b | -0.30 | 0.0700 |
| F2rl3 | 0.75 | 0.0000 |
| Tom1 | -0.72 | 0.0049 |
| Hmox1 | -1.36 | 0.0000 |
| Mcm5 | 0.41 | 0.0313 |
| Rbmxl1 | -0.46 | 0.0003 |
| Slc10a7 | 0.44 | 0.0356 |
| Abce1 | -0.42 | 0.0004 |
| Gab1 | 1.20 | 0.0000 |
| Inpp4b | 0.72 | 0.0000 |
| Zfp330 | -0.51 | 0.0003 |
| Rnf150 | 1.22 | 0.0013 |
| Tbc1d9 | -0.61 | 0.0052 |
| Ucp1 | -1.65 | 0.0110 |
| Tecr | -1.28 | 0.0000 |
| Pkn1 | 0.66 | 0.0023 |
| Cd97 | 0.97 | 0.0000 |
| Lphn1 | 1.13 | 0.0000 |
| Asf1b | 0.45 | 0.0367 |
| Prkaca | 0.79 | 0.0000 |
| Zswim4 | -0.85 | 0.0000 |
| Ccdc130 | -0.61 | 0.0105 |
| Ier2 | -1.11 | 0.0000 |
| Nfix | 0.43 | 0.0582 |
| Lyl1 | 1.60 | 0.0000 |
| Calr | -0.33 | 0.0112 |
| Gcdh | 0.81 | 0.0001 |
| Dnase2a | 0.58 | 0.0259 |
| Junb | -2.00 | 0.0000 |
| 2310036O22Rik | -0.45 | 0.0664 |
| Zfp791 | 1.65 | 0.0087 |
| Gpt2 | -0.34 | 0.0512 |
| Dnaja2 | -0.47 | 0.0006 |
| Phkb | 0.39 | 0.0074 |
| Siah1a | -0.48 | 0.0100 |
| N4bp1 | -0.51 | 0.0074 |
| Nod2 | -1.14 | 0.0000 |
| Chd9 | 0.73 | 0.0338 |
| Rbl2 | 1.46 | 0.0000 |
| Rpgrip1l | 0.65 | 0.0016 |
| Fto | 0.57 | 0.0003 |
| Irx3 | 0.85 | 0.0000 |
| Irx5 | 0.73 | 0.0014 |
| Mmp2 | 0.76 | 0.0650 |
| Lpcat2 | 2.27 | 0.0795 |
| Amfr | -0.24 | 0.0674 |
| Bbs2 | 0.32 | 0.0877 |
| Herpud1 | -1.23 | 0.0000 |
| Nlrc5 | -1.43 | 0.0001 |
| Cpne2 | 0.40 | 0.0130 |
| Rspry1 | 0.47 | 0.0101 |
| Pllp | 1.95 | 0.0307 |
| Cx3cl1 | -1.81 | 0.0000 |
| Ccl17 | -2.29 | 0.0953 |
| Ciapin1 | -0.45 | 0.0071 |
| Coq9 | 0.40 | 0.0052 |
| Polr2c | -0.44 | 0.0784 |
| Dok4 | 0.59 | 0.0000 |
| Gpr97 | -1.24 | 0.0000 |
| Katnb1 | 0.55 | 0.0052 |
| Kifc3 | 0.45 | 0.0007 |
| Cngb1 | 0.79 | 0.0129 |
| Mmp15 | 1.45 | 0.0000 |
| Cfap20 | 0.32 | 0.0276 |
| Csnk2a2 | -0.40 | 0.0051 |
| Gins3 | 0.55 | 0.0304 |
| Ndrg4 | 1.21 | 0.0000 |
| Slc38a7 | -0.30 | 0.0311 |
| Cdh5 | -0.34 | 0.0357 |
| Bean1 | -0.92 | 0.0000 |
| Cklf | -0.83 | 0.0000 |
| Cmtm3 | -0.54 | 0.0022 |
| Pdp2 | -1.07 | 0.0000 |
| Fam96b | -0.59 | 0.0029 |
| Ces2g | 0.58 | 0.0052 |
| Hsf4 | 0.60 | 0.0827 |
| E2f4 | -0.29 | 0.0271 |
| Elmo3 | -0.60 | 0.0373 |
| Hsd11b2 | 1.57 | 0.0321 |
| Fam65a | -0.36 | 0.0005 |
| Rltpr | -0.78 | 0.0116 |
| Nutf2 | -0.64 | 0.0136 |
| Pskh1 | 0.38 | 0.0100 |
| Ctrl | -1.04 | 0.0117 |
| Psmb10 | -0.85 | 0.0000 |
| Lcat | -1.29 | 0.0000 |
| Slc12a4 | -0.69 | 0.0000 |
| Dpep3 | -2.23 | 0.0207 |
| Ddx28 | -0.65 | 0.0000 |
| Nfatc3 | 0.49 | 0.0215 |
| Pla2g15 | 0.72 | 0.0000 |
| Slc7a6 | -0.54 | 0.0081 |
| Slc7a6os | -0.80 | 0.0002 |
| Prmt7 | -0.60 | 0.0000 |
| Tango6 | 0.90 | 0.0005 |
| Has3 | 2.77 | 0.0000 |
| Cirh1a | -0.64 | 0.0000 |
| Sntb2 | 0.49 | 0.0579 |
| Vps4a | 0.25 | 0.0905 |
| Nip7 | -0.88 | 0.0000 |
| Cyb5b | -0.36 | 0.0745 |
| Nob1 | -0.72 | 0.0000 |
| Ist1 | -0.46 | 0.0002 |
| Phlpp2 | -0.32 | 0.0717 |
| Cmtr2 | -0.58 | 0.0055 |
| Vac14 | -0.28 | 0.0560 |
| Mtss1l | 1.18 | 0.0000 |
| Il34 | -1.21 | 0.0578 |
| Fuk | 0.85 | 0.0000 |
| Aars | -0.98 | 0.0000 |
| Pdpr | 1.24 | 0.0019 |
| Rfwd3 | 0.32 | 0.0815 |
| Mlkl | -0.67 | 0.0000 |
| Wdr59 | -0.71 | 0.0002 |
| Znrf1 | -0.93 | 0.0000 |
| Bcar1 | -0.33 | 0.0046 |
| Tmem231 | 0.83 | 0.0000 |
| Kars | -0.31 | 0.0022 |
| Terf2ip | 0.39 | 0.0630 |
| Nudt7 | 0.95 | 0.0000 |
| Wwox | 0.54 | 0.0154 |
| Cmc2 | 0.52 | 0.0573 |
| Gcsh | -0.33 | 0.0390 |
| Pkd1l2 | 0.65 | 0.0782 |
| Plcg2 | 0.71 | 0.0002 |
| Mphosph6 | -0.46 | 0.0932 |
| Hsdl1 | -0.27 | 0.0593 |
| Kcng4 | 4.31 | 0.0090 |
| Tldc1 | 0.65 | 0.0007 |
| Klhl36 | 1.50 | 0.0000 |
| Usp10 | -0.55 | 0.0000 |
| Zdhhc7 | 0.43 | 0.0004 |
| 6430548M08Rik | 0.96 | 0.0000 |
| Gins2 | 0.37 | 0.0584 |
| Fbxo31 | 0.71 | 0.0000 |
| Map1lc3b | 0.30 | 0.0236 |
| Slc7a5 | -1.42 | 0.0000 |
| Zfpm1 | 1.01 | 0.0000 |
| Mvd | -4.30 | 0.0000 |
| Ctu2 | -1.29 | 0.0000 |
| Cbfa2t3 | 0.75 | 0.0000 |
| Acsf3 | 0.43 | 0.0049 |
| Ankrd11 | -0.67 | 0.0010 |
| Rpl13 | -0.55 | 0.0374 |
| Spata33 | 0.77 | 0.0855 |
| Fanca | 0.66 | 0.0011 |
| Tcf25 | -0.24 | 0.0913 |
| Tubb3 | -1.07 | 0.0000 |
| Gas8 | -0.58 | 0.0111 |
| Rhou | -0.52 | 0.0079 |
| Rab4a | 0.56 | 0.0305 |
| Acta1 | 0.64 | 0.0125 |
| Abcb10 | -0.40 | 0.0450 |
| Cog2 | -0.24 | 0.0757 |
| 2310022B05Rik | 0.64 | 0.0000 |
| Arv1 | -0.75 | 0.0000 |
| 2810004N23Rik | -0.53 | 0.0001 |
| Tsnax | 0.36 | 0.0038 |
| Disc1 | 0.92 | 0.0000 |
| Sipa1l2 | -0.84 | 0.0000 |
| Ntpcr | 0.43 | 0.0490 |
| Pcnxl2 | -1.68 | 0.0864 |
| Slc35f3 | 1.76 | 0.0229 |
| Coa6 | 0.49 | 0.0995 |
| Gm17296 | 0.65 | 0.0205 |
| Nrp1 | 0.88 | 0.0003 |
| 2610044O15Rik8 | 0.44 | 0.0500 |
| Flnb | -0.99 | 0.0003 |
| Abhd6 | 0.52 | 0.0009 |
| Pdhb | -0.33 | 0.0219 |
| 4930452B06Rik | 1.22 | 0.0000 |
| Ptprg | 0.56 | 0.0774 |
| Il3ra | -0.57 | 0.0451 |
| Oxsm | 0.35 | 0.0648 |
| Thrb | 1.01 | 0.0000 |
| Rpl15 | -0.46 | 0.0986 |
| Ube2e1 | -0.34 | 0.0780 |
| Ube2e2 | -0.77 | 0.0001 |
| Nid2 | 0.40 | 0.0820 |
| Dnajc9 | 0.35 | 0.0189 |
| Cfap70 | -1.78 | 0.0535 |
| Mss51 | -0.91 | 0.0235 |
| Fut11 | 0.37 | 0.0118 |
| Zswim8 | -0.53 | 0.0000 |
| Camk2g | 0.27 | 0.0125 |
| Plau | -0.42 | 0.0007 |
| Kat6b | 0.30 | 0.0920 |
| Comtd1 | -0.93 | 0.0029 |
| Zfp503 | 2.22 | 0.0000 |
| Rps24 | -0.50 | 0.0305 |
| Zmiz1 | 0.34 | 0.0398 |
| Zcchc24 | 2.03 | 0.0000 |
| Anxa11 | 0.29 | 0.0118 |
| Dnah12 | 1.71 | 0.0131 |
| Asb14 | -0.97 | 0.0883 |
| Il17rd | 1.51 | 0.0000 |
| Arhgef3 | 0.42 | 0.0215 |
| Wnt5a | 1.65 | 0.0000 |
| Selk | -0.86 | 0.0007 |
| Tkt | -0.91 | 0.0000 |
| Prkcd | -0.77 | 0.0000 |
| Rft1 | 0.34 | 0.0738 |
| Tmem110 | 0.91 | 0.0000 |
| Itih3 | -2.40 | 0.0000 |
| Nek4 | 0.59 | 0.0001 |
| Gnl3 | -1.60 | 0.0000 |
| Nt5dc2 | 1.14 | 0.0000 |
| Stab1 | 0.66 | 0.0005 |
| Nisch | 0.27 | 0.0526 |
| Sema3g | 0.61 | 0.0006 |
| Phf7 | 0.46 | 0.0520 |
| Dnah1 | 0.64 | 0.0113 |
| Mettl6 | -0.42 | 0.0512 |
| Eaf1 | -0.60 | 0.0020 |
| Btd | -0.52 | 0.0127 |
| Ankrd28 | -0.48 | 0.0366 |
| Timm23 | -0.56 | 0.0039 |
| Ercc6 | -0.87 | 0.0000 |
| Gprin2 | 2.24 | 0.0099 |
| Fam35a | 0.52 | 0.0167 |
| Ccser2 | 0.83 | 0.0000 |
| Sh2d4b | 0.88 | 0.0002 |
| Tspan14 | 0.88 | 0.0000 |
| Txndc16 | 0.67 | 0.0002 |
| Gpr137c | 0.94 | 0.0356 |
| Ero1l | -0.56 | 0.0282 |
| Psmc6 | -0.41 | 0.0012 |
| Fermt2 | -0.50 | 0.0047 |
| Samd4 | -0.59 | 0.0024 |
| Gch1 | -0.76 | 0.0000 |
| Wdhd1 | 0.56 | 0.0319 |
| Dlgap5 | 0.40 | 0.0466 |
| Ktn1 | -0.40 | 0.0037 |
| Peli2 | 1.84 | 0.0715 |
| Tmem260 | 0.62 | 0.0018 |
| Ap5m1 | 0.34 | 0.0949 |
| Ttc5 | 0.54 | 0.0000 |
| Apex1 | -0.83 | 0.0000 |
| Tmem55b | -0.93 | 0.0000 |
| Arhgef40 | -0.56 | 0.0000 |
| Rab2b | 0.59 | 0.0360 |
| Mettl3 | -0.59 | 0.0000 |
| Sall2 | 1.41 | 0.0000 |
| Abhd4 | 0.96 | 0.0000 |
| Oxa1l | 0.37 | 0.0047 |
| Slc7a7 | 0.44 | 0.0002 |
| Mrpl52 | -0.64 | 0.0029 |
| Haus4 | 0.44 | 0.0542 |
| 4931414P19Rik | 1.17 | 0.0000 |
| Ppp1r3e | 0.92 | 0.0546 |
| Pabpn1 | -0.46 | 0.0069 |
| Slc22a17 | 1.75 | 0.0000 |
| Myh7 | 0.48 | 0.0118 |
| Ngdn | -0.32 | 0.0803 |
| Zfhx2 | 0.33 | 0.0715 |
| Thtpa | 0.85 | 0.0004 |
| Dhrs4 | 0.30 | 0.0370 |
| Pck2 | -0.72 | 0.0000 |
| Psme1 | -0.77 | 0.0001 |
| Emc9 | 0.75 | 0.0005 |
| Psme2 | -0.80 | 0.0052 |
| Rnf31 | -0.44 | 0.0021 |
| Ipo4 | -1.05 | 0.0000 |
| Mdp1 | 0.30 | 0.0491 |
| Tinf2 | -0.29 | 0.0715 |
| Nop9 | -0.46 | 0.0009 |
| Ltb4r1 | -1.01 | 0.0031 |
| Nfatc4 | 0.39 | 0.0994 |
| Nynrin | 1.81 | 0.0062 |
| Khnyn | 0.71 | 0.0047 |
| Atp12a | 2.90 | 0.0000 |
| Cenpj | 0.37 | 0.0263 |
| Cryl1 | 1.46 | 0.0000 |
| Lats2 | -0.23 | 0.0720 |
| Ska3 | 0.87 | 0.0002 |
| Phf11d | -0.70 | 0.0374 |
| Cab39l | 0.72 | 0.0002 |
| Cdadc1 | 0.50 | 0.0015 |
| Spata13 | -0.47 | 0.0231 |
| C1qtnf9 | -0.68 | 0.0613 |
| Mipep | 0.34 | 0.0546 |
| Ebpl | 1.03 | 0.0000 |
| Spryd7 | -0.87 | 0.0000 |
| Ints6 | -0.26 | 0.0900 |
| Fdft1 | -4.13 | 0.0000 |
| Fam167a | -1.30 | 0.0000 |
| Xkr6 | -0.93 | 0.0000 |
| Pinx1 | -1.02 | 0.0000 |
| Sox7 | -1.50 | 0.0000 |
| Msra | -0.59 | 0.0138 |
| Kif13b | 0.57 | 0.0126 |
| Hmbox1 | 0.60 | 0.0000 |
| Ints9 | 0.59 | 0.0001 |
| Zfp395 | 2.28 | 0.0000 |
| Elp3 | 0.28 | 0.0243 |
| Esco2 | 0.46 | 0.0461 |
| Ptk2b | 0.91 | 0.0000 |
| Stmn4 | -2.33 | 0.0609 |
| Dpysl2 | -0.66 | 0.0972 |
| Bnip3l | 0.39 | 0.0023 |
| Ppp2r2a | -0.50 | 0.0000 |
| Cdca2 | 0.34 | 0.0508 |
| Kctd9 | -0.36 | 0.0520 |
| Gnrh1 | -1.13 | 0.0094 |
| Dock5 | -0.50 | 0.0143 |
| Slc25a37 | -0.89 | 0.0000 |
| Entpd4 | 1.10 | 0.0000 |
| Gm21685 | 0.80 | 0.0043 |
| Chmp7 | -0.20 | 0.0732 |
| Tnfrsf10b | -0.94 | 0.0000 |
| Rhobtb2 | -0.55 | 0.0000 |
| Egr3 | -1.74 | 0.0000 |
| 9930012K11Rik | 1.33 | 0.0000 |
| Pdlim2 | 0.51 | 0.0059 |
| Sorbs3 | 0.37 | 0.0051 |
| Piwil2 | -2.62 | 0.0385 |
| Phyhip | -0.61 | 0.0037 |
| Reep4 | -0.48 | 0.0159 |
| Hr | -0.45 | 0.0019 |
| Nudt18 | -0.50 | 0.0014 |
| Fgf17 | -0.86 | 0.0827 |
| Npm2 | -0.92 | 0.0808 |
| Xpo7 | 0.38 | 0.0433 |
| Rcbtb2 | 0.71 | 0.0000 |
| Rb1 | 0.36 | 0.0361 |
| Lpar6 | 0.87 | 0.0000 |
| Itm2b | 0.31 | 0.0120 |
| Med4 | -0.34 | 0.0713 |
| Nudt15 | 1.20 | 0.0028 |
| Esd | -1.16 | 0.0000 |
| Lrch1 | 0.76 | 0.0000 |
| Cog3 | -0.22 | 0.0963 |
| Gtf2f2 | -0.43 | 0.0047 |
| Kctd4 | -1.34 | 0.0000 |
| Gpalpp1 | 0.46 | 0.0429 |
| Nufip1 | -0.77 | 0.0000 |
| Lacc1 | -0.98 | 0.0000 |
| Ccdc122 | -1.47 | 0.0001 |
| Akap11 | 0.48 | 0.0888 |
| Vwa8 | 0.57 | 0.0004 |
| Rgcc | 2.10 | 0.0076 |
| Naa16 | -0.33 | 0.0580 |
| Kbtbd7 | 1.10 | 0.0000 |
| Zbtbd6 | 0.86 | 0.0979 |
| Pcdh17 | 1.89 | 0.0000 |
| Pcdh9 | -1.45 | 0.0009 |
| Dach1 | -0.96 | 0.0001 |
| Dis3 | -0.48 | 0.0037 |
| Klf5 | -1.84 | 0.0000 |
| Tbc1d4 | 0.97 | 0.0000 |
| Irg1 | -3.66 | 0.0000 |
| Rbm26 | -0.32 | 0.0408 |
| Spry2 | -1.02 | 0.0000 |
| Gpr180 | 0.44 | 0.0008 |
| Mbnl2 | 0.51 | 0.0005 |
| Ipo5 | -0.57 | 0.0004 |
| Farp1 | -0.35 | 0.0247 |
| Dock9 | 0.57 | 0.0003 |
| Ubac2 | 0.38 | 0.0054 |
| Pcca | 0.48 | 0.0023 |
| Ggact | 0.32 | 0.0520 |
| Tmtc4 | 0.73 | 0.0008 |
| Casp4 | -3.04 | 0.0000 |
| Dync2h1 | 0.61 | 0.0226 |
| Dcun1d5 | -0.52 | 0.0003 |
| Birc3 | -1.94 | 0.0000 |
| 9230110C19Rik | 0.71 | 0.0469 |
| Jrkl | -0.36 | 0.0350 |
| Maml2 | 0.38 | 0.0137 |
| Sesn3 | 0.69 | 0.0011 |
| Fut4 | 1.02 | 0.0000 |
| Ankrd49 | -0.77 | 0.0000 |
| Mre11a | 0.37 | 0.0588 |
| Panx1 | 0.57 | 0.0014 |
| Hephl1 | 2.01 | 0.0000 |
| Vstm5 | -0.57 | 0.0151 |
| Med17 | -0.25 | 0.0576 |
| Taf1d | -1.07 | 0.0000 |
| Slc36a4 | 0.44 | 0.0363 |
| Naalad2 | -1.33 | 0.0824 |
| Zfp266 | 0.26 | 0.0511 |
| Olfm2 | 0.38 | 0.0164 |
| Ppan | -1.61 | 0.0000 |
| Eif3g | -0.52 | 0.0093 |
| Dnmt1 | 0.44 | 0.0138 |
| Icam1 | -3.85 | 0.0000 |
| Icam5 | -1.04 | 0.0067 |
| Fdx1l | -1.31 | 0.0200 |
| Tyk2 | -0.24 | 0.0920 |
| Cdc37 | -0.22 | 0.0387 |
| Kri1 | -0.74 | 0.0000 |
| Cdkn2d | 1.01 | 0.0000 |
| Slc44a2 | 0.50 | 0.0000 |
| Qtrt1 | -0.93 | 0.0000 |
| Tmed1 | 0.33 | 0.0921 |
| AB124611 | -2.52 | 0.0198 |
| 1810026J23Rik | 0.53 | 0.0119 |
| Ldlr | -4.67 | 0.0000 |
| Spc24 | 0.55 | 0.0023 |
| Kank2 | 0.89 | 0.0000 |
| Dock6 | 0.45 | 0.0051 |
| Gm6484 | 1.07 | 0.0000 |
| Swsap1 | 0.66 | 0.0032 |
| Epor | 2.54 | 0.0000 |
| Rgl3 | 0.61 | 0.0001 |
| Prkcsh | -0.22 | 0.0428 |
| Zfp653 | -0.84 | 0.0008 |
| Pigyl | 0.95 | 0.0000 |
| Zfp810 | 0.50 | 0.0088 |
| Vps26b | 0.63 | 0.0039 |
| Ncapd3 | 0.35 | 0.0956 |
| Jam3 | 0.99 | 0.0000 |
| Igsf9b | 1.12 | 0.0000 |
| St14 | 0.77 | 0.0005 |
| Prdm10 | -0.28 | 0.0824 |
| Fli1 | 0.95 | 0.0000 |
| St3gal4 | 0.66 | 0.0001 |
| Dcps | 0.31 | 0.0409 |
| Rpusd4 | -0.70 | 0.0008 |
| Chek1 | -0.59 | 0.0048 |
| Tmem218 | 2.27 | 0.0000 |
| Robo4 | -0.57 | 0.0000 |
| Robo3 | 0.60 | 0.0208 |
| Msantd2 | -0.59 | 0.0009 |
| Esam | 0.55 | 0.0000 |
| Vsig2 | 0.50 | 0.0002 |
| Siae | 1.41 | 0.0000 |
| Tbrg1 | -0.48 | 0.0021 |
| Olfr920 | -0.93 | 0.0026 |
| Scn3b | 1.88 | 0.0000 |
| Gramd1b | 2.92 | 0.0000 |
| Hspa8 | -0.31 | 0.0491 |
| Sc5d | -2.75 | 0.0000 |
| Oaf | -0.37 | 0.0033 |
| Usp2 | 1.38 | 0.0000 |
| Mfrp | 1.73 | 0.0709 |
| C2cd2l | 0.79 | 0.0000 |
| Dpagt1 | 0.55 | 0.0000 |
| Trappc4 | -0.34 | 0.0168 |
| Bcl9l | 0.93 | 0.0000 |
| Ddx6 | 0.40 | 0.0012 |
| Phldb1 | -0.91 | 0.0000 |
| Ift46 | 0.50 | 0.0028 |
| Fxyd2 | -3.20 | 0.0386 |
| Cep164 | 0.79 | 0.0000 |
| Bace1 | 1.11 | 0.0001 |
| Rnf214 | 0.49 | 0.0056 |
| Sidt2 | 0.50 | 0.0000 |
| Zpr1 | -0.75 | 0.0000 |
| Bud13 | -0.28 | 0.0608 |
| Nxpe2 | 1.50 | 0.0011 |
| Rexo2 | -0.37 | 0.0129 |
| Gm5617 | 0.42 | 0.0734 |
| Nnmt | -0.43 | 0.0340 |
| Usp28 | -0.58 | 0.0002 |
| Zw10 | 0.42 | 0.0282 |
| Ncam1 | 0.81 | 0.0076 |
| Rpl10-ps3 | -0.33 | 0.0669 |
| Plet1 | -0.96 | 0.0017 |
| Sdhd | 0.26 | 0.0751 |
| Dixdc1 | 2.16 | 0.0000 |
| 1110032A03Rik | 0.75 | 0.0002 |
| Fdxacb1 | -0.49 | 0.0388 |
| Sik2 | 0.67 | 0.0799 |
| Layn | 1.03 | 0.0000 |
| Btg4 | 1.18 | 0.0184 |
| Gm684 | 1.70 | 0.0000 |
| Arhgap20 | 2.03 | 0.0000 |
| Rdx | 0.45 | 0.0213 |
| Zc3h12c | -0.59 | 0.0341 |
| Ddx10 | -0.77 | 0.0000 |
| Exph5 | 3.22 | 0.0000 |
| Kdelc2 | 0.66 | 0.0000 |
| 4930550C14Rik | 0.54 | 0.0734 |
| Atm | 1.31 | 0.0007 |
| Acat1 | 0.36 | 0.0035 |
| Dmxl2 | 0.49 | 0.0238 |
| Idh3a | -0.30 | 0.0195 |
| Wdr61 | -0.38 | 0.0017 |
| Hykk | 1.84 | 0.0000 |
| Psma4 | -0.44 | 0.0693 |
| Fbxo22 | -0.30 | 0.0304 |
| Nrg4 | -0.87 | 0.0020 |
| Scaper | 0.43 | 0.0760 |
| Tspan3 | 0.46 | 0.0005 |
| Hmg20a | 0.36 | 0.0025 |
| Snx33 | 0.60 | 0.0000 |
| Man2c1 | -0.51 | 0.0375 |
| 1700017B05Rik | 0.40 | 0.0285 |
| Scamp5 | 0.72 | 0.0000 |
| Cox5a | 0.27 | 0.0771 |
| Fam219b | 0.31 | 0.0138 |
| Mpi | 0.33 | 0.0236 |
| Cplx3 | -1.37 | 0.0908 |
| Lman1l | -1.49 | 0.0007 |
| Cyp1a1 | 1.22 | 0.0000 |
| Sema7a | 1.95 | 0.0000 |
| Pml | 0.81 | 0.0000 |
| Cd276 | 1.31 | 0.0000 |
| Adpgk | -0.79 | 0.0000 |
| Bbs4 | 0.38 | 0.0499 |
| Parp6 | -0.63 | 0.0128 |
| Gramd2 | -4.38 | 0.0000 |
| Myo9a | -0.41 | 0.0431 |
| Lrrc49 | 0.53 | 0.0001 |
| Tle3 | 0.51 | 0.0012 |
| Anp32a | 0.74 | 0.0000 |
| Coro2b | 0.96 | 0.0010 |
| Cln6 | 1.08 | 0.0000 |
| Calml4 | 1.08 | 0.0000 |
| Pias1 | 0.40 | 0.0068 |
| Aagab | 0.30 | 0.0284 |
| Smad3 | -0.38 | 0.0393 |
| Smad6 | 1.09 | 0.0000 |
| Slc24a1 | -0.84 | 0.0782 |
| Ptplad1 | 0.47 | 0.0003 |
| Igdcc3 | 2.55 | 0.0715 |
| Ankdd1a | -2.33 | 0.0522 |
| Plekho2 | -0.67 | 0.0002 |
| Pif1 | 0.77 | 0.0003 |
| Rbpms2 | -1.09 | 0.0000 |
| Oaz2 | 0.25 | 0.0696 |
| 2810417H13Rik | 0.40 | 0.0121 |
| Snx1 | -0.27 | 0.0322 |
| Fbxl22 | -0.49 | 0.0843 |
| Aph1b | 1.04 | 0.0000 |
| Rab8b | 0.68 | 0.0000 |
| Tpm1 | -0.44 | 0.0004 |
| C2cd4a | 2.83 | 0.0990 |
| Vps13c | 1.36 | 0.0206 |
| Anxa2 | -0.68 | 0.0000 |
| Myo1e | -0.51 | 0.0000 |
| Ccnb2 | 0.63 | 0.0000 |
| Sltm | -0.93 | 0.0000 |
| Tcf12 | 0.38 | 0.0588 |
| Mns1 | 1.50 | 0.0006 |
| Tex9 | 1.42 | 0.0015 |
| Pygo1 | 1.15 | 0.0441 |
| Ccpg1os | 1.19 | 0.0005 |
| Ccpg1 | 1.40 | 0.0150 |
| Pigb | 0.96 | 0.0000 |
| Rsl24d1 | -0.44 | 0.0054 |
| Myo5c | 1.74 | 0.0236 |
| Gnb5 | 0.76 | 0.0006 |
| Leo1 | -1.11 | 0.0000 |
| Gclc | -0.33 | 0.0164 |
| Fbxo9 | 0.41 | 0.0012 |
| Gsta1 | -5.11 | 0.0014 |
| Slc17a5 | 0.60 | 0.0000 |
| Cd109 | 0.78 | 0.0025 |
| Filip1 | 1.34 | 0.0000 |
| Senp6 | -0.48 | 0.0000 |
| Myo6 | 0.63 | 0.0067 |
| Phip | 0.30 | 0.0414 |
| Lca5 | -0.46 | 0.0110 |
| Bckdhb | 0.69 | 0.0010 |
| Me1 | -0.77 | 0.0000 |
| Cyb5r4 | 0.45 | 0.0255 |
| Nt5e | -1.21 | 0.0003 |
| Syncrip | -0.61 | 0.0000 |
| Tmed3 | 0.45 | 0.0002 |
| Ctsh | 0.70 | 0.0000 |
| Adamts7 | -0.82 | 0.0002 |
| Tbc1d2b | 1.60 | 0.0000 |
| Plscr2 | 0.79 | 0.0018 |
| Plscr4 | 1.64 | 0.0000 |
| Plod2 | 0.33 | 0.0542 |
| 1190002N15Rik | 0.87 | 0.0035 |
| Chst2 | 1.29 | 0.0000 |
| Pcolce2 | 0.80 | 0.0000 |
| Trpc1 | -0.96 | 0.0023 |
| Atr | -0.46 | 0.0073 |
| Tfdp2 | 1.53 | 0.0000 |
| Rasa2 | -1.09 | 0.0000 |
| Zbtb38 | -0.36 | 0.0205 |
| Spsb4 | 1.40 | 0.0389 |
| Mrps22 | -0.58 | 0.0040 |
| Faim | -0.52 | 0.0178 |
| Mras | 1.27 | 0.0000 |
| Dzip1l | 0.64 | 0.0900 |
| Ppp2r3a | -0.70 | 0.0000 |
| Ephb1 | 0.60 | 0.0001 |
| Cep63 | -0.33 | 0.0969 |
| Slco2a1 | 1.57 | 0.0050 |
| Rab6b | 1.10 | 0.0002 |
| Cdv3 | -0.63 | 0.0000 |
| Acad11 | 1.25 | 0.0000 |
| Atp2c1 | 0.41 | 0.0091 |
| Pik3r4 | 0.39 | 0.0415 |
| Ppm1m | 0.44 | 0.0671 |
| Twf2 | 0.38 | 0.0662 |
| Rpl29 | -0.92 | 0.0024 |
| Abhd14b | 0.44 | 0.0165 |
| Pcbp4 | -0.34 | 0.0909 |
| Rrp9 | -1.42 | 0.0000 |
| Iqcf1 | -2.71 | 0.0184 |
| Vprbp | -0.28 | 0.0497 |
| Manf | -0.37 | 0.0434 |
| Mapkapk3 | 0.71 | 0.0043 |
| Cish | 0.76 | 0.0374 |
| Tmem115 | -0.27 | 0.0248 |
| Ifrd2 | -0.86 | 0.0000 |
| Gnat1 | -0.80 | 0.0077 |
| Rbm6 | -0.32 | 0.0089 |
| Fam212a | 0.82 | 0.0000 |
| Nicn1 | 1.42 | 0.0000 |
| Tcta | 0.46 | 0.0085 |
| 1700102P08Rik | -1.27 | 0.0019 |
| Klhdc8b | -0.46 | 0.0445 |
| Impdh2 | -0.48 | 0.0258 |
| Ndufaf3 | 0.39 | 0.0704 |
| Wdr6 | 1.03 | 0.0001 |
| P4htm | 1.07 | 0.0020 |
| Arih2 | -0.53 | 0.0000 |
| Slc25a20 | 0.33 | 0.0476 |
| Nckipsd | 0.47 | 0.0009 |
| Pfkfb4 | -1.55 | 0.0000 |
| Plxnb1 | 1.28 | 0.0000 |
| Cspg5 | -1.64 | 0.0033 |
| Elp6 | 0.53 | 0.0363 |
| Ptpn23 | -1.04 | 0.0000 |
| Kif9 | 0.98 | 0.0025 |
| Tmie | 1.61 | 0.0000 |
| Rtp3 | 1.52 | 0.0000 |
| Lrrfip2 | -0.84 | 0.0000 |
| Mlh1 | 0.38 | 0.0535 |
| Ubp1 | -0.47 | 0.0114 |
| Cmtm8 | 1.26 | 0.0011 |
| Gpd1l | 0.92 | 0.0000 |
| Osbpl10 | 0.34 | 0.0980 |
| Tgfbr2 | 0.88 | 0.0000 |
| Rbms3 | 0.45 | 0.0216 |
| Itga9 | 0.44 | 0.0377 |
| Ctdspl | 1.29 | 0.0222 |
| Plcd1 | -0.40 | 0.0110 |
| Dlec1 | 1.24 | 0.0154 |
| Acvr2b | 0.69 | 0.0157 |
| Exog | 0.65 | 0.0004 |
| Gorasp1 | 0.60 | 0.0004 |
| Ttc21a | 2.27 | 0.0056 |
| Csrnp1 | -1.04 | 0.0000 |
| Myrip | 2.48 | 0.0000 |
| Rpl14 | -0.57 | 0.0805 |
| Sec22c | 1.12 | 0.0000 |
| Zfp651 | 0.59 | 0.0000 |
| Pomgnt2 | 1.21 | 0.0000 |
| Clec3b | 1.82 | 0.0024 |
| Tmem158 | 1.83 | 0.0000 |
| Ppp2r3d | -1.17 | 0.0000 |
| Patz1 | 0.91 | 0.0000 |
| Limk2 | -0.28 | 0.0795 |
| Selm | 0.54 | 0.0179 |
| Osbp2 | -1.41 | 0.0000 |
| Slc35e4 | -0.20 | 0.0746 |
| Pes1 | -0.46 | 0.0000 |
| Sec14l2 | 0.98 | 0.0000 |
| Ccdc157 | 0.94 | 0.0000 |
| Tbc1d10a | -0.29 | 0.0507 |
| Gatsl3 | 1.59 | 0.0000 |
| Lif | -2.17 | 0.0261 |
| Ascc2 | -0.36 | 0.0091 |
| Zmat5 | 0.46 | 0.0497 |
| Nf2 | 0.53 | 0.0018 |
| Nipsnap1 | 0.84 | 0.0000 |
| Thoc5 | -0.33 | 0.0028 |
| Gas2l1 | -0.28 | 0.0077 |
| Ewsr1 | -0.50 | 0.0106 |
| Kremen1 | 0.72 | 0.0001 |
| Xbp1 | -0.71 | 0.0000 |
| Ccdc117 | 0.37 | 0.1000 |
| Polm | -0.64 | 0.0178 |
| Pold2 | -0.55 | 0.0000 |
| Myl7 | -3.16 | 0.0106 |
| Ykt6 | 0.37 | 0.0254 |
| Ddx56 | -0.45 | 0.0006 |
| Ppia | -0.64 | 0.0251 |
| H2afv | 0.55 | 0.0086 |
| Ccm2 | -0.28 | 0.0486 |
| Ramp3 | -0.96 | 0.0000 |
| Tns3 | -0.75 | 0.0000 |
| Cobl | -1.09 | 0.0000 |
| Egfr | -1.75 | 0.0000 |
| Plek | 0.83 | 0.0011 |
| Wdr92 | 0.39 | 0.0321 |
| Pno1 | -0.66 | 0.0001 |
| Etaa1 | 0.65 | 0.0045 |
| Cep68 | 0.98 | 0.0000 |
| Slc1a4 | -1.11 | 0.0000 |
| Lgalsl | 1.19 | 0.0000 |
| Vps54 | -0.24 | 0.0989 |
| Mdh1 | 0.34 | 0.0201 |
| Wdpcp | 0.78 | 0.0063 |
| Ehbp1 | 0.74 | 0.0000 |
| Tmem17 | 0.62 | 0.0953 |
| Zrsr1 | 0.53 | 0.0000 |
| Cct4 | -0.37 | 0.0038 |
| 0610010F05Rik | -0.50 | 0.0148 |
| Rel | -0.75 | 0.0002 |
| Pnpt1 | -0.40 | 0.0079 |
| Cfap36 | 0.52 | 0.0055 |
| Rps27a | -0.75 | 0.0077 |
| Rtn4 | -0.79 | 0.0000 |
| Eml6 | 1.01 | 0.0000 |
| Acyp2 | 0.32 | 0.0838 |
| Erlec1 | 0.37 | 0.0212 |
| Snrnp25 | 0.71 | 0.0011 |
| Mpg | 0.97 | 0.0000 |
| Stk10 | 0.52 | 0.0029 |
| Npm1 | -0.97 | 0.0000 |
| Lcp2 | -5.19 | 0.0000 |
| Slit3 | 0.68 | 0.0693 |
| Pank3 | -1.44 | 0.0000 |
| Rars | -1.35 | 0.0000 |
| Wwc1 | -1.67 | 0.0382 |
| Hmmr | 0.66 | 0.0053 |
| Ccng1 | 0.31 | 0.0149 |
| C1qtnf2 | 1.68 | 0.0001 |
| Rnf145 | 0.59 | 0.0051 |
| Ebf1 | 0.95 | 0.0000 |
| Thg1l | -0.66 | 0.0003 |
| Adam19 | 0.43 | 0.0889 |
| Gnb2l1 | -0.27 | 0.0966 |
| Psme2b | -0.72 | 0.0000 |
| Olfr56 | -1.13 | 0.0845 |
| Tgtp1 | -0.96 | 0.0024 |
| 9930111J21Rik2 | -0.64 | 0.0122 |
| Tgtp2 | -1.25 | 0.0025 |
| Btnl9 | 1.81 | 0.0000 |
| Flt4 | -0.38 | 0.0757 |
| Cnot6 | 0.24 | 0.0366 |
| Gfpt2 | -0.89 | 0.0005 |
| Mapk9 | 0.37 | 0.0020 |
| Rnf130 | 0.70 | 0.0000 |
| Tbc1d9b | 0.53 | 0.0010 |
| 3010026O09Rik | 0.73 | 0.0109 |
| Sqstm1 | -1.31 | 0.0000 |
| Maml1 | 0.40 | 0.0350 |
| Hnrnph1 | -0.65 | 0.0001 |
| Zfp354c | 2.96 | 0.0001 |
| Col23a1 | 0.35 | 0.0299 |
| Hnrnpab | -0.37 | 0.0002 |
| Nhp2 | -0.43 | 0.0164 |
| N4bp3 | -0.44 | 0.0000 |
| Cdkl3 | 0.61 | 0.0329 |
| Hspa4 | -0.29 | 0.0168 |
| Gdf9 | -1.93 | 0.0083 |
| Sept8 | -0.66 | 0.0001 |
| Kif3a | -0.94 | 0.0000 |
| Il4 | -1.30 | 0.0000 |
| Irf1 | -0.97 | 0.0000 |
| Slc22a21 | -1.70 | 0.0000 |
| Slc22a4 | -1.66 | 0.0000 |
| P4ha2 | -0.45 | 0.0006 |
| Csf2 | -5.04 | 0.0001 |
| Fnip1 | -0.52 | 0.0000 |
| Gpx3 | -0.73 | 0.0175 |
| Tnip1 | -1.52 | 0.0000 |
| Ccdc69 | 1.62 | 0.0000 |
| Gm2a | 1.27 | 0.0000 |
| Slc36a1 | 0.61 | 0.0009 |
| G3bp1 | -0.52 | 0.0000 |
| Nmur2 | -1.85 | 0.0036 |
| Fam114a2 | 0.29 | 0.0258 |
| Galnt10 | 0.82 | 0.0041 |
| Larp1 | -0.48 | 0.0024 |
| Gm12258 | 0.73 | 0.0083 |
| Zfp39 | 0.96 | 0.0191 |
| Rnf187 | 0.27 | 0.0248 |
| Hist3h2a | 0.48 | 0.0500 |
| Wnt9a | -1.59 | 0.0000 |
| Jmjd4 | -0.57 | 0.0001 |
| Zfp867 | -0.73 | 0.0000 |
| Mprip | -0.46 | 0.0229 |
| Pemt | -0.65 | 0.0300 |
| Srebf1 | -0.39 | 0.0013 |
| Gid4 | 0.47 | 0.0032 |
| Drg2 | -0.43 | 0.0180 |
| Flii | -0.34 | 0.0128 |
| Shmt1 | -0.79 | 0.0035 |
| Tmem11 | -0.57 | 0.0019 |
| Natd1 | 0.59 | 0.0012 |
| Map2k3 | -0.48 | 0.0000 |
| Mapk7 | -0.73 | 0.0000 |
| Epn2 | 0.83 | 0.0000 |
| Grap | 0.47 | 0.0123 |
| Fam83g | -1.35 | 0.0000 |
| Ulk2 | 0.42 | 0.0949 |
| Specc1 | -0.47 | 0.0138 |
| Zswim7 | 0.46 | 0.0375 |
| Trim16 | -0.85 | 0.0000 |
| Elac2 | -0.37 | 0.0043 |
| Tmem220 | 1.10 | 0.0000 |
| Adprm | 0.30 | 0.0942 |
| Ntn1 | 1.20 | 0.0000 |
| Pik3r5 | -1.74 | 0.0000 |
| Pik3r6 | 1.28 | 0.0000 |
| Ndel1 | -0.30 | 0.0621 |
| Slc25a35 | 2.09 | 0.0000 |
| Ctc1 | 0.49 | 0.0004 |
| Aurkb | 0.30 | 0.0397 |
| 2310047M10Rik | -0.45 | 0.0074 |
| Aloxe3 | -2.34 | 0.0000 |
| Cntrob | 0.37 | 0.0353 |
| Trappc1 | 0.47 | 0.0145 |
| Kcnab3 | 1.03 | 0.0245 |
| Chd3 | 0.79 | 0.0000 |
| Cyb5d1 | 1.00 | 0.0049 |
| Tmem88 | 1.92 | 0.0000 |
| Kdm6b | -0.74 | 0.0000 |
| Dnah2 | 1.48 | 0.0293 |
| Wrap53 | 0.46 | 0.0071 |
| Trp53 | -1.01 | 0.0000 |
| Fxr2 | -0.28 | 0.0196 |
| Mpdu1 | 0.30 | 0.0267 |
| Cd68 | -1.17 | 0.0000 |
| Eif4a1 | -1.16 | 0.0000 |
| Tnfsf12Tnfsf13 | 0.86 | 0.0005 |
| Tnfsf13 | 0.82 | 0.0241 |
| Slc35g3 | 1.87 | 0.0089 |
| Zbtb4 | 0.52 | 0.0143 |
| Chrnb1 | -1.07 | 0.0000 |
| Fgf11 | 0.85 | 0.0082 |
| Tmem102 | 2.19 | 0.0110 |
| Plscr3 | -0.43 | 0.0007 |
| Tnk1 | -1.39 | 0.0000 |
| Kctd11 | -0.37 | 0.0152 |
| Acap1 | 0.70 | 0.0000 |
| 2810408A11Rik | 0.60 | 0.0616 |
| Slc2a4 | -0.62 | 0.0395 |
| Dvl2 | -0.43 | 0.0007 |
| Acadvl | 0.35 | 0.0231 |
| Dlg4 | 0.67 | 0.0000 |
| Asgr2 | 1.33 | 0.0005 |
| Slc16a13 | -1.40 | 0.0000 |
| Bcl6b | -0.28 | 0.0130 |
| 0610010K14Rik | -0.65 | 0.0365 |
| Alox12 | 0.87 | 0.0121 |
| Med11 | -1.30 | 0.0000 |
| Cxcl16 | -4.10 | 0.0000 |
| Zmynd15 | -4.35 | 0.0000 |
| Psmb6 | -0.35 | 0.0924 |
| Mink1 | 0.51 | 0.0018 |
| Slc25a11 | 0.58 | 0.0000 |
| Pfn1 | -0.61 | 0.0106 |
| Spag7 | -0.51 | 0.0095 |
| Zfp3 | 1.14 | 0.0481 |
| C1qbp | -0.67 | 0.0000 |
| Nlrp1b | 0.90 | 0.0000 |
| Aipl1 | 0.94 | 0.0654 |
| Pitpnm3 | 1.50 | 0.0000 |
| Txndc17 | -0.33 | 0.0207 |
| Fbxo39 | -1.20 | 0.0353 |
| Mybbp1a | -1.18 | 0.0000 |
| Spns2 | 0.98 | 0.0000 |
| Ube2g1 | 0.24 | 0.0596 |
| P2rx1 | 1.65 | 0.0340 |
| Camkk1 | 1.46 | 0.0004 |
| 1200014J11Rik | 0.53 | 0.0035 |
| Gsg2 | 0.38 | 0.0204 |
| P2rx5 | -0.93 | 0.0033 |
| Shpk | 1.17 | 0.0000 |
| Rap1gap2 | -0.47 | 0.0267 |
| E130309D14Rik | 1.08 | 0.0215 |
| Cluh | -0.58 | 0.0005 |
| Mnt | -0.81 | 0.0000 |
| Sgsm2 | 0.53 | 0.0016 |
| Tsr1 | -0.77 | 0.0049 |
| Smg6 | 0.42 | 0.0024 |
| Hic1 | 0.73 | 0.0000 |
| Ovca2 | -0.91 | 0.0009 |
| Dph1 | -0.47 | 0.0176 |
| Rpa1 | 0.86 | 0.0001 |
| Serpinf2 | -2.10 | 0.0001 |
| Tlcd2 | -0.83 | 0.0099 |
| Inpp5k | 0.49 | 0.0000 |
| Doc2b | 2.72 | 0.0101 |
| Rph3al | 1.41 | 0.0000 |
| Fam101b | 1.40 | 0.0000 |
| Vps53 | 0.63 | 0.0000 |
| Glod4 | 1.20 | 0.0000 |
| Fam57a | 1.20 | 0.0000 |
| Gemin4 | 0.90 | 0.0201 |
| Rnmtl1 | 0.64 | 0.0000 |
| Nxn | 0.82 | 0.0000 |
| Timm22 | 0.47 | 0.0006 |
| Cpd | 0.63 | 0.0072 |
| Ssh2 | 0.35 | 0.0129 |
| Ankrd13b | -0.59 | 0.0068 |
| Git1 | -0.29 | 0.0040 |
| Trp53i13 | -0.33 | 0.0693 |
| Pipox | 2.15 | 0.0000 |
| Sez6 | 3.19 | 0.0103 |
| Phf12 | 0.26 | 0.0650 |
| Dhrs13 | 0.33 | 0.0981 |
| Traf4 | 0.93 | 0.0000 |
| Tlcd1 | -1.73 | 0.0000 |
| BC030499 | -0.96 | 0.0097 |
| Pigs | 0.48 | 0.0027 |
| Tmem199 | -0.36 | 0.0222 |
| Ift20 | -0.38 | 0.0480 |
| Tmem97 | -2.19 | 0.0000 |
| Nlk | -0.36 | 0.0213 |
| Lyrm9 | 0.81 | 0.0001 |
| Ksr1 | 0.84 | 0.0000 |
| Wsb1 | -0.61 | 0.0000 |
| Atad5 | 0.64 | 0.0130 |
| 5730455P16Rik | 0.60 | 0.0001 |
| Psmd11 | -0.48 | 0.0000 |
| Cdk5r1 | -0.61 | 0.0385 |
| Tmem98 | 0.81 | 0.0000 |
| Ccl2 | -5.01 | 0.0000 |
| Ccl7 | -4.81 | 0.0000 |
| Zfp830 | -0.38 | 0.0250 |
| Rffl | 0.58 | 0.0601 |
| Rad51d | 0.51 | 0.0086 |
| Nle1 | -0.90 | 0.0000 |
| Slfn9 | 0.55 | 0.0040 |
| Slfn8 | 0.90 | 0.0490 |
| Slfn2 | -0.47 | 0.0434 |
| Mmp28 | 0.86 | 0.0054 |
| Taf15 | -0.99 | 0.0000 |
| Ccl5 | -1.05 | 0.0036 |
| Synrg | 0.50 | 0.0142 |
| Tada2a | -0.28 | 0.0838 |
| Acaca | -0.34 | 0.0126 |
| Aatf | -0.84 | 0.0000 |
| Dhrs11 | 0.80 | 0.0000 |
| Ggnbp2 | -0.54 | 0.0000 |
| Appbp2 | -0.36 | 0.0068 |
| Ppm1d | 0.30 | 0.0964 |
| Bcas3 | 0.31 | 0.0161 |
| Tbx2 | 1.07 | 0.0000 |
| Brip1 | 0.62 | 0.0221 |
| Rps6kb1 | -0.26 | 0.0235 |
| Vmp1 | -1.67 | 0.0000 |
| Dhx40 | 0.24 | 0.0524 |
| Ypel2 | -0.45 | 0.0000 |
| Prr11 | 0.69 | 0.0000 |
| Ska2 | 0.54 | 0.0086 |
| Trim37 | 0.47 | 0.0806 |
| Ppm1e | 0.90 | 0.0016 |
| Rad51c | 0.86 | 0.0020 |
| Sept4 | 1.16 | 0.0000 |
| Mtmr4 | 0.50 | 0.0109 |
| Scpep1 | -0.46 | 0.0106 |
| Nog | -1.56 | 0.0424 |
| Mmd | 1.03 | 0.0001 |
| Stxbp4 | 1.66 | 0.0000 |
| Utp18 | -0.57 | 0.0000 |
| Mbtd1 | -0.31 | 0.0486 |
| Nme1 | -0.49 | 0.0246 |
| Spag9 | -0.49 | 0.0161 |
| Luc7l3 | -0.47 | 0.0022 |
| Abcc3 | 1.05 | 0.0000 |
| Acsf2 | 1.03 | 0.0000 |
| Chad | 1.32 | 0.0004 |
| Lrrc59 | -0.32 | 0.0840 |
| Xylt2 | 0.84 | 0.0000 |
| Samd14 | 0.39 | 0.0229 |
| Pdk2 | 1.69 | 0.0000 |
| Itga3 | -0.73 | 0.0000 |
| Fam117a | -0.25 | 0.0850 |
| Nxph3 | -1.08 | 0.0001 |
| Phb | -0.84 | 0.0003 |
| Zfp652 | 0.50 | 0.0039 |
| Abi3 | 0.81 | 0.0000 |
| Hoxb9 | -1.85 | 0.0000 |
| Hoxb8 | 0.79 | 0.0000 |
| Hoxb7 | 0.67 | 0.0000 |
| Hoxb6 | 0.45 | 0.0059 |
| Hoxb4 | -0.49 | 0.0442 |
| Nfe2l1 | -0.37 | 0.0822 |
| Copz2 | 0.53 | 0.0138 |
| Scrn2 | 1.02 | 0.0000 |
| Tbkbp1 | -0.25 | 0.0278 |
| Kpnb1 | -0.32 | 0.0132 |
| Npepps | -0.26 | 0.0300 |
| Srcin1 | 1.25 | 0.0828 |
| Mllt6 | 0.53 | 0.0000 |
| Cacnb1 | 0.95 | 0.0919 |
| Rpl19 | -0.67 | 0.0799 |
| Fbxl20 | 0.59 | 0.0015 |
| Stard3 | 0.43 | 0.0023 |
| Pgap3 | 0.51 | 0.0147 |
| Ormdl3 | 0.36 | 0.0020 |
| Csf3 | -3.17 | 0.0000 |
| Thra | 1.36 | 0.0000 |
| Nr1d1 | 0.89 | 0.0000 |
| Casc3 | -0.33 | 0.0203 |
| Rapgefl1 | 0.96 | 0.0163 |
| Igfbp4 | 0.91 | 0.0000 |
| Krt24 | -3.39 | 0.0110 |
| Krt10 | 0.52 | 0.0275 |
| Eif1 | -0.90 | 0.0000 |
| Jup | 0.87 | 0.0000 |
| Leprel4 | 0.83 | 0.0001 |
| Klhl11 | 1.29 | 0.0022 |
| Acly | -1.39 | 0.0000 |
| Ttc25 | -3.61 | 0.0651 |
| Cnp | 0.47 | 0.0004 |
| Dnajc7 | -0.32 | 0.0870 |
| Nkiras2 | 0.32 | 0.0650 |
| Ghdc | -0.26 | 0.0775 |
| Stat5a | 1.63 | 0.0000 |
| Ptrf | 0.42 | 0.0000 |
| Atp6v0a1 | -0.31 | 0.0017 |
| Naglu | 0.67 | 0.0000 |
| Mlx | 0.86 | 0.0000 |
| Tubg1 | 0.61 | 0.0004 |
| Tubg2 | 1.01 | 0.0054 |
| Cntnap1 | 0.34 | 0.0671 |
| Ccr10 | 1.28 | 0.0000 |
| Ezh1 | 0.50 | 0.0002 |
| Ramp2 | 0.44 | 0.0182 |
| Aoc2 | -0.92 | 0.0000 |
| Rpl27 | -0.65 | 0.0025 |
| Ifi35 | -0.32 | 0.0573 |
| Rnd2 | -0.49 | 0.0049 |
| Brca1 | 0.43 | 0.0516 |
| Rdm1 | 1.01 | 0.0002 |
| Etv4 | -1.47 | 0.0013 |
| Meox1 | -0.67 | 0.0810 |
| Mpp3 | 1.37 | 0.0046 |
| Mpp2 | 0.92 | 0.0000 |
| Hdac5 | 0.42 | 0.0007 |
| Ubtf | -0.30 | 0.0070 |
| Fam171a2 | 0.73 | 0.0000 |
| Fzd2 | 1.09 | 0.0006 |
| Adam11 | 1.66 | 0.0000 |
| Kif18b | 0.51 | 0.0489 |
| Dcakd | 0.36 | 0.0228 |
| Hexim1 | 0.37 | 0.0006 |
| Map3k14 | -0.32 | 0.0747 |
| Arhgap27 | 0.68 | 0.0000 |
| Plekhm1 | 0.75 | 0.0000 |
| Gosr2 | 0.33 | 0.0575 |
| Nsf | 0.58 | 0.0294 |
| Itgb3 | -0.85 | 0.0013 |
| Mettl2 | -0.47 | 0.0005 |
| Tlk2 | -0.45 | 0.0007 |
| Tanc2 | -0.67 | 0.0041 |
| Taco1 | 0.63 | 0.0019 |
| Ddx42 | -0.27 | 0.0117 |
| Ftsj3 | -0.80 | 0.0000 |
| Psmc5 | -0.50 | 0.0126 |
| Smarcd2 | 0.58 | 0.0000 |
| Scn4a | -1.34 | 0.0003 |
| Ern1 | 0.58 | 0.0821 |
| Tex2 | 0.75 | 0.0001 |
| Polg2 | -0.57 | 0.0539 |
| Kpna2 | -0.43 | 0.0076 |
| Pitpnc1 | 0.51 | 0.0044 |
| Psmd12 | -0.33 | 0.0051 |
| Helz | 0.80 | 0.0000 |
| Cacng1 | 1.86 | 0.0003 |
| Cacng4 | 1.65 | 0.0000 |
| Prkca | -0.65 | 0.0034 |
| Axin2 | 4.71 | 0.0052 |
| Rgs9 | -1.29 | 0.0131 |
| Arsg | 0.49 | 0.0564 |
| Wipi1 | 0.28 | 0.0709 |
| Abca5 | -0.57 | 0.0542 |
| Kcnj2 | 0.37 | 0.0241 |
| Slc39a11 | 0.40 | 0.0114 |
| Sstr2 | 0.27 | 0.0620 |
| Rab37 | 2.46 | 0.0142 |
| Slc9a3r1 | 0.29 | 0.0583 |
| Tmem104 | 0.40 | 0.0057 |
| Fads6 | 4.54 | 0.0045 |
| Ush1g | 2.12 | 0.0016 |
| Kctd2 | 0.44 | 0.0009 |
| Slc16a5 | 1.36 | 0.0048 |
| Armc7 | -0.43 | 0.0014 |
| Hn1 | -0.26 | 0.0668 |
| Nup85 | -0.40 | 0.0282 |
| 2310067B10Rik | 0.65 | 0.0000 |
| Caskin2 | 0.40 | 0.0045 |
| Itgb4 | 0.75 | 0.0000 |
| Galk1 | 0.37 | 0.0440 |
| Unc13d | -1.15 | 0.0001 |
| Wbp2 | -0.25 | 0.0234 |
| Trim47 | -1.72 | 0.0000 |
| Trim65 | 0.78 | 0.0000 |
| Mrpl38 | -0.73 | 0.0006 |
| Exoc7 | 0.41 | 0.0043 |
| Sphk1 | -0.95 | 0.0212 |
| Ube2o | -0.43 | 0.0029 |
| Aanat | -1.65 | 0.0626 |
| Jmjd6 | -0.79 | 0.0000 |
| Srsf2 | -1.10 | 0.0000 |
| Syngr2 | -0.33 | 0.0188 |
| Tk1 | 0.48 | 0.0060 |
| Afmid | 0.72 | 0.0097 |
| Socs3 | -1.59 | 0.0000 |
| Pgs1 | -0.66 | 0.0000 |
| Dnah17 | -0.60 | 0.0100 |
| Cyth1 | -0.76 | 0.0000 |
| Timp2 | 0.88 | 0.0000 |
| Cant1 | 0.35 | 0.0020 |
| Engase | 1.61 | 0.0000 |
| Cbx2 | 0.90 | 0.0000 |
| Cbx8 | 1.02 | 0.0000 |
| Cbx4 | 0.70 | 0.0000 |
| Tbc1d16 | 2.06 | 0.0000 |
| Ccdc40 | 2.98 | 0.0000 |
| Eif4a3 | -0.26 | 0.0974 |
| Sgsh | 1.17 | 0.0000 |
| Slc26a11 | -0.44 | 0.0072 |
| Nptx1 | 0.84 | 0.0035 |
| Cep131 | 0.84 | 0.0001 |
| Enthd2 | -0.46 | 0.0431 |
| Bahcc1 | 1.15 | 0.0000 |
| Ccdc137 | -0.43 | 0.0008 |
| Hgs | -0.51 | 0.0000 |
| Slc25a10 | 0.37 | 0.0355 |
| Npb | -0.86 | 0.0956 |
| Pcyt2 | -2.89 | 0.0000 |
| Mafg | -1.04 | 0.0000 |
| Aspscr1 | -0.48 | 0.0113 |
| Dcxr | 1.05 | 0.0000 |
| Hmga1-rs1 | -0.63 | 0.0000 |
| Rfng | 0.99 | 0.0000 |
| Gps1 | -0.55 | 0.0395 |
| Fasn | -1.56 | 0.0000 |
| Hexdc | -0.51 | 0.0101 |
| Narf | 0.80 | 0.0007 |
| Foxk2 | -0.73 | 0.0000 |
| Rab40b | 2.18 | 0.0035 |
| Fn3krp | 0.74 | 0.0005 |
| Fn3k | 1.76 | 0.0703 |
| Metrnl | -1.15 | 0.0000 |
| Gdi2 | 0.20 | 0.0991 |
| Asb13 | 0.90 | 0.0001 |
| Net1 | 0.78 | 0.0000 |
| Akr1c13 | 0.58 | 0.0098 |
| Akr1e1 | 0.36 | 0.0754 |
| Klf6 | -1.71 | 0.0000 |
| Pitrm1 | -0.55 | 0.0068 |
| Pfkp | -0.44 | 0.0083 |
| Idi1 | -5.31 | 0.0000 |
| Gtpbp4 | -0.74 | 0.0000 |
| Larp4b | -0.35 | 0.0111 |
| Mtr | 0.54 | 0.0046 |
| Heatr1 | -0.90 | 0.0000 |
| Gpr137b | -0.39 | 0.0002 |
| Nid1 | -0.60 | 0.0319 |
| Lyst | 0.47 | 0.0983 |
| Tbce | -0.56 | 0.0000 |
| Mrpl32 | -0.45 | 0.0262 |
| Yae1d1 | -0.32 | 0.0296 |
| Vps41 | 0.29 | 0.0506 |
| Epdr1 | 0.92 | 0.0677 |
| Elmo1 | 0.29 | 0.0408 |
| Trim27 | -0.42 | 0.0003 |
| Zfp322a | 0.35 | 0.0237 |
| Hfe | 0.83 | 0.0000 |
| Lrrc16a | 0.42 | 0.0056 |
| BC005537 | -0.60 | 0.0000 |
| Aldh5a1 | 2.28 | 0.0000 |
| Mrs2 | 0.38 | 0.0061 |
| Cdkal1 | 0.36 | 0.0613 |
| E2f3 | -0.39 | 0.0021 |
| Dusp22 | 0.68 | 0.0008 |
| Foxc1 | -0.54 | 0.0136 |
| Serpinb1c | -1.64 | 0.0001 |
| Serpinb9b | -1.89 | 0.0000 |
| Bphl | 0.93 | 0.0000 |
| Tubb2a | -1.04 | 0.0000 |
| Tubb2b | -1.78 | 0.0000 |
| Psmg4 | -0.88 | 0.0035 |
| Pxdc1 | -1.68 | 0.0000 |
| Cdyl | -0.67 | 0.0005 |
| Rpp40 | -0.94 | 0.0053 |
| Fars2 | 0.35 | 0.0330 |
| Riok1 | -0.36 | 0.0041 |
| Snrnp48 | -0.47 | 0.0128 |
| Bloc1s5 | 0.37 | 0.0436 |
| Eef1e1 | -1.36 | 0.0000 |
| Slc35b3 | -0.46 | 0.0010 |
| Pak1ip1 | -0.57 | 0.0003 |
| Nedd9 | 0.38 | 0.0858 |
| Tmem170b | -1.34 | 0.0000 |
| Edn1 | -1.19 | 0.0000 |
| Sirt5 | 0.99 | 0.0000 |
| Rnf182 | 1.49 | 0.0000 |
| Mylip | 1.63 | 0.0201 |
| Gmpr | 1.40 | 0.0000 |
| Atxn1 | -0.57 | 0.0000 |
| Kif13a | 0.47 | 0.0424 |
| Kdm1b | 0.64 | 0.0004 |
| Dek | 0.39 | 0.0546 |
| Zfp169 | 0.45 | 0.0804 |
| Ptpdc1 | 0.51 | 0.0002 |
| Ecm2 | 1.94 | 0.0000 |
| Nol8 | -0.47 | 0.0011 |
| Iars | -0.83 | 0.0018 |
| Fbxw17 | -0.46 | 0.0032 |
| Gadd45g | -1.05 | 0.0000 |
| Auh | 0.70 | 0.0004 |
| Nfil3 | -1.27 | 0.0000 |
| Simc1 | -0.59 | 0.0001 |
| Nop16 | -0.69 | 0.0000 |
| Uimc1 | -0.41 | 0.0557 |
| Nsd1 | 0.33 | 0.0192 |
| Mxd3 | 0.94 | 0.0000 |
| Rgs14 | 1.31 | 0.0095 |
| Dbn1 | -0.38 | 0.0001 |
| Pdlim7 | -0.95 | 0.0000 |
| Dok3 | -0.78 | 0.0084 |
| Tmed9 | -0.30 | 0.0608 |
| B4galt7 | -0.70 | 0.0000 |
| Txndc15 | 0.46 | 0.0006 |
| Pcbd2 | 0.48 | 0.0948 |
| Fbxl21 | 1.04 | 0.0024 |
| Tgfbi | 1.75 | 0.0000 |
| Klhl3 | 1.74 | 0.0000 |
| Hnrnpa0 | -0.31 | 0.0153 |
| 2210016F16Rik | 0.39 | 0.0464 |
| Hnrnpk | -0.53 | 0.0001 |
| Isca1 | 0.48 | 0.0109 |
| Ctla2b | -1.17 | 0.0000 |
| Ctla2a | -0.59 | 0.0011 |
| Zfp808 | 0.55 | 0.0421 |
| 6720489N17Rik | 1.28 | 0.0017 |
| Fbp2 | 1.50 | 0.0000 |
| Fbp1 | 1.25 | 0.0000 |
| Habp4 | 0.43 | 0.0145 |
| Aaed1 | 0.69 | 0.0002 |
| Ctsl | -0.33 | 0.0013 |
| Cdk20 | 0.64 | 0.0186 |
| Mterf3 | -0.51 | 0.0002 |
| Zfp759 | 1.81 | 0.0000 |
| Rsl1 | -0.44 | 0.0979 |
| Zfp458 | 0.65 | 0.0847 |
| Zfp457 | 1.79 | 0.0504 |
| Zfp953 | 0.48 | 0.0675 |
| Zfp273 | 1.36 | 0.0021 |
| Fastkd3 | -0.37 | 0.0582 |
| Nsun2 | -0.73 | 0.0000 |
| Adamts16 | 2.85 | 0.0514 |
| Slc6a3 | 1.40 | 0.0633 |
| Tert | 1.13 | 0.0026 |
| Slc12a7 | 0.98 | 0.0000 |
| Cep72 | 0.60 | 0.0093 |
| Exoc3 | 0.27 | 0.0838 |
| Ahrr | 1.42 | 0.0000 |
| Pdcd6 | -0.36 | 0.0025 |
| Gm10116 | -0.53 | 0.0081 |
| Erap1 | -0.58 | 0.0119 |
| Cast | -0.37 | 0.0033 |
| Ell2 | -1.03 | 0.0000 |
| Glrx | -0.50 | 0.0001 |
| Rhobtb3 | 0.39 | 0.0878 |
| Arsk | 0.78 | 0.0004 |
| Nr2f1 | 1.52 | 0.0014 |
| Arrdc3 | 1.42 | 0.0000 |
| Polr3g | -0.67 | 0.0034 |
| Mef2c | 1.17 | 0.0000 |
| Tmem161b | -0.50 | 0.0173 |
| Rps23 | -0.64 | 0.0466 |
| Ssbp2 | 0.90 | 0.0003 |
| Serinc5 | 2.43 | 0.0000 |
| Jmy | 0.74 | 0.0017 |
| Arsb | 1.31 | 0.0000 |
| Scamp1 | 0.48 | 0.0007 |
| Tbca | -0.50 | 0.0203 |
| Zbed3 | 0.40 | 0.0031 |
| Aggf1 | 0.46 | 0.0007 |
| F2rl1 | -1.47 | 0.0036 |
| F2r | 0.59 | 0.0109 |
| Poc5 | 0.49 | 0.0189 |
| Polk | 0.39 | 0.0742 |
| Hmgcr | -3.76 | 0.0000 |
| Hexb | 0.56 | 0.0505 |
| Enc1 | -0.59 | 0.0136 |
| Utp15 | -0.68 | 0.0000 |
| Ankra2 | -0.41 | 0.0954 |
| Btf3 | -0.60 | 0.0077 |
| Tmem171 | -1.24 | 0.0000 |
| Fcho2 | 0.39 | 0.0012 |
| Zfp366 | 1.39 | 0.0000 |
| Ptcd2 | 0.31 | 0.0593 |
| Mrps27 | 0.34 | 0.0116 |
| Map1b | -1.19 | 0.0000 |
| Mccc2 | 0.83 | 0.0000 |
| Naip2 | 1.20 | 0.0000 |
| Naip6 | 2.17 | 0.0000 |
| Ocln | 0.74 | 0.0034 |
| Ak6 | -0.59 | 0.0009 |
| Ccdc125 | 0.74 | 0.0211 |
| Cenph | 0.55 | 0.0247 |
| Slc30a5 | -0.33 | 0.0042 |
| Pik3r1 | 0.39 | 0.0019 |
| Mast4 | -0.64 | 0.0299 |
| Nln | -0.71 | 0.0000 |
| Adamts6 | 0.66 | 0.0048 |
| Cwc27 | -0.40 | 0.0305 |
| Ipo11 | 0.56 | 0.0005 |
| Dimt1 | -0.44 | 0.0799 |
| Zswim6 | -1.25 | 0.0000 |
| Smim15 | -0.57 | 0.0000 |
| Depdc1b | 0.86 | 0.0002 |
| Pde4d | 1.09 | 0.0001 |
| Plk2 | -0.29 | 0.0077 |
| Gpbp1 | -0.37 | 0.0035 |
| Map3k1 | 0.73 | 0.0000 |
| Il6st | -0.62 | 0.0051 |
| Ddx4 | 2.55 | 0.0018 |
| Ccno | -1.73 | 0.0010 |
| Arl15 | 0.57 | 0.0051 |
| Fst | -1.19 | 0.0000 |
| Itga2 | -0.94 | 0.0000 |
| Itga1 | 0.94 | 0.0000 |
| Pelo | -0.47 | 0.0243 |
| Parp8 | -1.09 | 0.0000 |
| Emb | 0.64 | 0.0015 |
| Mrps30 | -0.44 | 0.0846 |
| Hmgcs1 | -4.52 | 0.0000 |
| Nim1k | 1.20 | 0.0000 |
| Zfp131 | -0.86 | 0.0000 |
| Dtnb | 0.51 | 0.0010 |
| Efr3b | 0.63 | 0.0007 |
| Ncoa1 | 0.75 | 0.0003 |
| Fam228b | 1.91 | 0.0146 |
| Ubxn2a | 0.43 | 0.0597 |
| Rhob | -1.22 | 0.0000 |
| Sdc1 | 1.22 | 0.0000 |
| Wdr35 | 0.96 | 0.0141 |
| Mycn | 0.74 | 0.0763 |
| Ddx1 | -0.22 | 0.0538 |
| Fam84a | 0.62 | 0.0007 |
| Lpin1 | -1.83 | 0.0000 |
| Rock2 | -0.37 | 0.0908 |
| Pqlc3 | 0.38 | 0.0017 |
| Kcnf1 | 0.89 | 0.0349 |
| Pdia6 | -0.26 | 0.0139 |
| Atp6v1c2 | -2.71 | 0.0197 |
| Nol10 | -0.80 | 0.0000 |
| Odc1 | -1.58 | 0.0000 |
| Hpcal1 | 0.40 | 0.0007 |
| Taf1b | -0.32 | 0.0317 |
| Klf11 | -0.77 | 0.0000 |
| Mboat2 | 0.98 | 0.0000 |
| Id2 | 1.84 | 0.0000 |
| Rnaseh1 | -0.85 | 0.0000 |
| Adi1 | 0.56 | 0.0004 |
| Trappc12 | 0.35 | 0.0807 |
| Myt1l | -1.01 | 0.0237 |
| Sntg2 | 2.25 | 0.0000 |
| Tmem18 | 0.98 | 0.0000 |
| Fam110c | -1.87 | 0.0000 |
| Lamb1 | -0.74 | 0.0000 |
| Dld | -0.48 | 0.0000 |
| Dus4l | -0.78 | 0.0000 |
| Cog5 | 0.60 | 0.0007 |
| Ccdc71l | -0.97 | 0.0000 |
| Nampt | -0.80 | 0.0000 |
| Atxn7l1 | 0.91 | 0.0000 |
| Twistnb | -0.59 | 0.0001 |
| Ahr | 1.86 | 0.0000 |
| Tspan13 | 1.33 | 0.0000 |
| Ankmy2 | 0.74 | 0.0000 |
| Meox2 | 2.73 | 0.0000 |
| Agmo | 3.05 | 0.0686 |
| Scin | 1.59 | 0.0000 |
| Ifrd1 | -2.72 | 0.0000 |
| Dock4 | -0.87 | 0.0018 |
| Lrrn3 | 2.13 | 0.0921 |
| Dnajb9 | -0.88 | 0.0000 |
| Dtd2 | 0.88 | 0.0000 |
| Nubpl | 0.58 | 0.0288 |
| Egln3 | 2.09 | 0.0000 |
| Sptssa | 0.99 | 0.0000 |
| Cfl2 | -0.37 | 0.0009 |
| Baz1a | -0.59 | 0.0036 |
| Fam177a | -0.48 | 0.0002 |
| 1700047I17Rik2 | -0.52 | 0.0003 |
| Ppp2r3c | -0.36 | 0.0076 |
| Psma6 | -0.63 | 0.0000 |
| Nfkbia | -3.30 | 0.0000 |
| Clec14a | 1.99 | 0.0000 |
| Trappc6b | -0.47 | 0.0009 |
| Pnn | -0.42 | 0.0002 |
| Ctage5 | -0.66 | 0.0000 |
| Prpf39 | -0.69 | 0.0002 |
| Fancm | 0.61 | 0.0014 |
| Rps29 | -0.89 | 0.0571 |
| Nemf | -0.23 | 0.0731 |
| Arf6 | -0.36 | 0.0964 |
| L2hgdh | -0.33 | 0.0694 |
| Atl1 | 0.95 | 0.0000 |
| Sav1 | 0.38 | 0.0692 |
| Psma3 | -0.34 | 0.0667 |
| Arid4a | 0.50 | 0.0058 |
| 2700049A03Rik | 0.80 | 0.0001 |
| Jkamp | 0.28 | 0.0565 |
| Dhrs7 | 0.91 | 0.0000 |
| Ppm1a | -0.24 | 0.0962 |
| Six4 | 0.70 | 0.0102 |
| Slc38a6 | 1.20 | 0.0000 |
| Hif1a | -0.52 | 0.0614 |
| Snapc1 | -0.59 | 0.0021 |
| Rhoj | 0.39 | 0.0064 |
| Sgpp1 | 0.74 | 0.0073 |
| Hspa2 | 0.54 | 0.0000 |
| Sptb | 0.92 | 0.0002 |
| Max | -0.44 | 0.0002 |
| Eif2s1 | -0.56 | 0.0000 |
| Pigh | 1.01 | 0.0000 |
| Rdh11 | -0.83 | 0.0000 |
| Rdh12 | 0.63 | 0.0050 |
| Actn1 | -0.93 | 0.0000 |
| Dcaf5 | 0.57 | 0.0000 |
| Erh | -0.48 | 0.0801 |
| 4933426M11Rik | 0.34 | 0.0564 |
| Adam4 | -1.29 | 0.0020 |
| Ttc9 | -0.59 | 0.0007 |
| Sipa1l1 | 0.56 | 0.0009 |
| Dcaf4 | 0.90 | 0.0000 |
| Rbm25 | -0.79 | 0.0000 |
| Papln | 0.69 | 0.0529 |
| 2410016O06Rik | -0.57 | 0.0028 |
| Acot6 | 1.90 | 0.0034 |
| Dnal1 | 0.94 | 0.0000 |
| Ptgr2 | 0.96 | 0.0002 |
| Aldh6a1 | 0.93 | 0.0000 |
| Abcd4 | -0.50 | 0.0097 |
| Npc2 | -0.31 | 0.0073 |
| Isca2 | -0.42 | 0.0219 |
| Fcf1 | -0.89 | 0.0001 |
| Dlst | -0.51 | 0.0000 |
| Rps6kl1 | -0.46 | 0.0704 |
| Pgf | -1.58 | 0.0000 |
| Eif2b2 | -0.45 | 0.0016 |
| Zc2hc1c | -0.56 | 0.0409 |
| Nek9 | 0.54 | 0.0046 |
| Fos | -1.82 | 0.0000 |
| Mfsd7c | -2.03 | 0.0000 |
| 0610007P14Rik | -2.16 | 0.0000 |
| Tgfb3 | 2.61 | 0.0000 |
| Angel1 | 0.43 | 0.0023 |
| Tmed8 | 0.72 | 0.0042 |
| Noxred1 | -0.73 | 0.0394 |
| Ahsa1 | -0.29 | 0.0214 |
| Sptlc2 | -0.45 | 0.0020 |
| Alkbh1 | -0.83 | 0.0000 |
| Cep128 | 0.69 | 0.0019 |
| Gtf2a1 | -0.80 | 0.0013 |
| Flrt2 | 1.86 | 0.0000 |
| Galc | 0.76 | 0.0034 |
| Eml5 | -0.68 | 0.0002 |
| Ttc8 | 0.68 | 0.0000 |
| Nrde2 | 0.27 | 0.0687 |
| Calm1 | -0.35 | 0.0114 |
| Rps6ka5 | 1.50 | 0.0000 |
| Ccdc88c | 0.90 | 0.0001 |
| Smek1 | -0.37 | 0.0018 |
| Tc2n | 0.51 | 0.0000 |
| Fbln5 | 0.84 | 0.0000 |
| Cpsf2 | -0.53 | 0.0011 |
| Rin3 | 0.26 | 0.0328 |
| Itpk1 | 1.37 | 0.0000 |
| Tmem251 | -0.75 | 0.0000 |
| AK010878 | -0.39 | 0.0567 |
| Btbd7 | 0.38 | 0.0608 |
| Ifi27l2a | 0.84 | 0.0000 |
| Ifi27l2b | 1.06 | 0.0000 |
| Syne3 | 1.03 | 0.0090 |
| Cyp46a1 | 1.48 | 0.0029 |
| Eml1 | 0.49 | 0.0005 |
| Slc25a29 | 0.71 | 0.0334 |
| Slc25a47 | -0.60 | 0.0522 |
| Ppp2r5c | 0.48 | 0.0008 |
| Hsp90aa1 | -0.68 | 0.0000 |
| Mok | 1.05 | 0.0563 |
| Cinp | -0.32 | 0.0405 |
| Traf3 | -0.69 | 0.0005 |
| Amn | -1.95 | 0.0024 |
| A230065H16Rik | -1.69 | 0.0522 |
| Exoc3l4 | -0.97 | 0.0001 |
| Tnfaip2 | -1.28 | 0.0000 |
| Eif5 | -0.63 | 0.0000 |
| Ckb | 0.73 | 0.0000 |
| Klc1 | -0.38 | 0.0001 |
| Xrcc3 | -0.37 | 0.0495 |
| Ppp1r13b | -0.49 | 0.0034 |
| Aspg | 1.99 | 0.0026 |
| Kif26a | 0.70 | 0.0007 |
| A530016L24Rik | 2.07 | 0.0000 |
| Siva1 | 0.46 | 0.0261 |
| Akt1 | -0.26 | 0.0582 |
| Ahnak2 | -0.48 | 0.0002 |
| Jag2 | 0.62 | 0.0000 |
| Nudt14 | 0.44 | 0.0326 |
| Btbd6 | 0.68 | 0.0002 |
| Pacs2 | -0.29 | 0.0508 |
| Crip2 | 0.34 | 0.0824 |
| 4930427A07Rik | 0.66 | 0.0018 |
| Wdr60 | 0.47 | 0.0051 |
| Esyt2 | -0.33 | 0.0218 |
| Cdca7l | 1.60 | 0.0000 |
| Dnah11 | 2.57 | 0.0000 |
| Sp4 | 1.06 | 0.0000 |
| Ghr | 0.22 | 0.0663 |
| Oxct1 | 0.45 | 0.0035 |
| Card6 | 1.20 | 0.0000 |
| Osmr | -1.95 | 0.0000 |
| Lifr | 1.27 | 0.0000 |
| Gdnf | -1.81 | 0.0000 |
| 2410089E03Rik | 0.35 | 0.0560 |
| Nadk2 | 0.89 | 0.0000 |
| Skp2 | 0.40 | 0.0154 |
| Dnajc21 | -0.81 | 0.0000 |
| Brix1 | -0.68 | 0.0000 |
| Amacr | 0.67 | 0.0020 |
| Adamts12 | 1.40 | 0.0000 |
| Tars | -1.29 | 0.0000 |
| Cdh6 | 0.86 | 0.0716 |
| Acot10 | -0.51 | 0.0274 |
| Basp1 | -1.41 | 0.0000 |
| Myo10 | -0.42 | 0.0355 |
| Fbxl7 | 1.13 | 0.0000 |
| Trio | -0.75 | 0.0001 |
| Dap | 0.50 | 0.0153 |
| Fam173b | 0.45 | 0.0028 |
| Sdc2 | -0.37 | 0.0676 |
| Matn2 | -1.27 | 0.0000 |
| Rpl30 | -1.07 | 0.0000 |
| Pop1 | -0.85 | 0.0000 |
| Ankrd46 | 0.70 | 0.0046 |
| Zfp706 | 0.37 | 0.0017 |
| Grhl2 | -2.02 | 0.0000 |
| Klf10 | -0.23 | 0.0390 |
| Dcaf13 | -0.69 | 0.0000 |
| Rims2 | -0.49 | 0.0001 |
| Zfpm2 | -1.76 | 0.0000 |
| Oxr1 | -0.65 | 0.0015 |
| Eif3e | -0.32 | 0.0728 |
| Nudcd1 | -0.51 | 0.0046 |
| Eny2 | -0.38 | 0.0008 |
| Utp23 | -0.55 | 0.0002 |
| Med30 | 0.53 | 0.0103 |
| Samd12 | 1.22 | 0.0000 |
| Deptor | 0.85 | 0.0000 |
| Mtbp | -0.72 | 0.0000 |
| Sntb1 | 1.24 | 0.0001 |
| Has2 | -2.64 | 0.0000 |
| Zhx2 | -0.43 | 0.0728 |
| Derl1 | -0.22 | 0.0436 |
| 9130401M01Rik | -0.78 | 0.0001 |
| Atad2 | 0.35 | 0.0220 |
| Wdyhv1 | -0.75 | 0.0029 |
| Fbxo32 | -0.55 | 0.0131 |
| Trmt12 | 0.30 | 0.0999 |
| Tatdn1 | -0.41 | 0.0274 |
| Mtss1 | 0.49 | 0.0000 |
| Sqle | -3.48 | 0.0000 |
| Trib1 | -0.89 | 0.0000 |
| Fam84b | -0.48 | 0.0648 |
| Myc | -1.55 | 0.0000 |
| Asap1 | 0.44 | 0.0068 |
| Tmem71 | 0.94 | 0.0000 |
| Ndrg1 | -1.47 | 0.0000 |
| St3gal1 | -0.41 | 0.0228 |
| Khdrbs3 | -0.55 | 0.0201 |
| Col22a1 | -0.49 | 0.0532 |
| Trappc9 | 0.39 | 0.0240 |
| Chrac1 | -0.40 | 0.0514 |
| Ptk2 | 0.32 | 0.0621 |
| Dennd3 | -0.46 | 0.0087 |
| Ptp4a3 | 0.94 | 0.0000 |
| Them6 | 1.22 | 0.0000 |
| Lynx1 | 0.49 | 0.0029 |
| Ly6a | -0.32 | 0.0446 |
| Ly6c1 | 0.35 | 0.0053 |
| Ly6c2 | 0.63 | 0.0018 |
| Ly6g | -1.63 | 0.0690 |
| Gpihbp1 | 0.72 | 0.0000 |
| Rhpn1 | 1.64 | 0.0027 |
| Mafa | 1.35 | 0.0128 |
| Gsdmd | -1.31 | 0.0000 |
| Naprt | 0.44 | 0.0652 |
| Eef1d | -0.55 | 0.0017 |
| Pycrl | -0.38 | 0.0172 |
| Tsta3 | -0.33 | 0.0487 |
| Zfp623 | -0.44 | 0.0069 |
| Ccdc166 | 0.89 | 0.0996 |
| Scrib | -0.33 | 0.0450 |
| Puf60 | -0.37 | 0.0087 |
| Hgh1 | -0.59 | 0.0000 |
| Mroh1 | -0.36 | 0.0116 |
| Bop1 | -0.74 | 0.0000 |
| Hsf1 | -0.68 | 0.0003 |
| Fbxl6 | -0.74 | 0.0006 |
| Slc52a2 | -0.69 | 0.0001 |
| Cpsf1 | -0.32 | 0.0309 |
| Slc39a4 | -2.81 | 0.0000 |
| Tonsl | 0.60 | 0.0002 |
| Kifc2 | 1.01 | 0.0636 |
| Mfsd3 | 0.56 | 0.0101 |
| Zfp251 | 0.45 | 0.0043 |
| Zfp7 | -0.96 | 0.0008 |
| Apol8 | -1.30 | 0.0000 |
| Myh9 | -0.48 | 0.0243 |
| Eif3d | -0.38 | 0.0003 |
| Ift27 | 0.55 | 0.0041 |
| Csf2rb2 | 1.88 | 0.0000 |
| Csf2rb | 2.12 | 0.0000 |
| Tst | 0.75 | 0.0366 |
| Kctd17 | 0.45 | 0.0033 |
| C1qtnf6 | 0.56 | 0.0000 |
| Cyth4 | -0.60 | 0.0000 |
| Mfng | 1.06 | 0.0000 |
| Card10 | 1.16 | 0.0000 |
| Cdc42ep1 | 0.25 | 0.0140 |
| Lgals2 | 0.67 | 0.0172 |
| Sh3bp1 | 1.49 | 0.0727 |
| Nol12 | -0.60 | 0.0213 |
| H1f0 | 0.47 | 0.0000 |
| Gcat | -1.28 | 0.0735 |
| Slc16a8 | -0.69 | 0.0395 |
| Baiap2l2 | -1.32 | 0.0215 |
| Pla2g6 | -0.49 | 0.0225 |
| Csnk1e | -0.68 | 0.0000 |
| Cby1 | 1.13 | 0.0000 |
| Gtpbp1 | 0.49 | 0.0007 |
| Sun2 | -0.83 | 0.0000 |
| Nptxr | 0.26 | 0.0465 |
| Cbx6 | 0.93 | 0.0000 |
| Apobec3 | 0.26 | 0.0693 |
| Cbx7 | 1.46 | 0.0000 |
| Pdgfb | -1.47 | 0.0000 |
| Rpl3 | -0.72 | 0.0010 |
| Tab1 | 0.36 | 0.0730 |
| Mief1 | -0.45 | 0.0053 |
| Atf4 | -1.69 | 0.0000 |
| Rps19bp1 | -0.71 | 0.0027 |
| Tnrc6b | 0.56 | 0.0010 |
| Adsl | -0.24 | 0.0635 |
| Slc25a17 | -0.32 | 0.0187 |
| L3mbtl2 | 0.40 | 0.0022 |
| Chadl | 0.84 | 0.0513 |
| Zc3h7b | 0.40 | 0.0369 |
| Tef | 1.01 | 0.0000 |
| Pmm1 | 0.65 | 0.0059 |
| Desi1 | 0.36 | 0.0086 |
| Nhp2l1 | -0.53 | 0.0000 |
| Ccdc134 | -1.07 | 0.0000 |
| Srebf2 | -2.24 | 0.0000 |
| Shisa8 | -2.59 | 0.0000 |
| Serhl | 0.25 | 0.0974 |
| Rrp7a | -0.62 | 0.0000 |
| Ttll1 | 0.49 | 0.0005 |
| Ttll12 | 0.50 | 0.0053 |
| Parvb | -0.38 | 0.0007 |
| Parvg | -1.37 | 0.0000 |
| 1810041L15Rik | -2.18 | 0.0062 |
| Ldoc1l | 1.21 | 0.0097 |
| Phf21b | 0.70 | 0.0014 |
| 5031439G07Rik | -0.47 | 0.0107 |
| Fbln1 | 0.92 | 0.0069 |
| Ttc38 | 2.05 | 0.0000 |
| Gtse1 | 0.61 | 0.0001 |
| Celsr1 | 0.66 | 0.0394 |
| Cerk | 0.28 | 0.0173 |
| Tbc1d22a | 0.73 | 0.0000 |
| Brd1 | -0.63 | 0.0000 |
| Zbed4 | -0.49 | 0.0010 |
| Pim3 | -0.53 | 0.0002 |
| Mov10l1 | -1.19 | 0.0007 |
| Mapk11 | -0.43 | 0.0171 |
| Ppp6r2 | 0.57 | 0.0000 |
| Tymp | 0.48 | 0.0266 |
| Odf3b | 1.68 | 0.0000 |
| Cpt1b | -0.85 | 0.0284 |
| Arsa | -0.69 | 0.0000 |
| Alg10b | 0.72 | 0.0076 |
| Cpne8 | -0.33 | 0.0072 |
| Abcd2 | 2.18 | 0.0018 |
| Lrrk2 | 0.82 | 0.0015 |
| Zcrb1 | -0.41 | 0.0877 |
| Pus7l | -0.63 | 0.0000 |
| Ano6 | 0.42 | 0.0935 |
| Slc38a2 | -1.49 | 0.0000 |
| Amigo2 | 0.33 | 0.0227 |
| Endou | -1.53 | 0.0547 |
| Rapgef3 | -0.41 | 0.0072 |
| Hdac7 | 0.67 | 0.0000 |
| Vdr | 1.65 | 0.0262 |
| Tmem106c | 0.55 | 0.0072 |
| Ccdc184 | -0.51 | 0.0002 |
| Kansl2 | -0.35 | 0.0630 |
| Adcy6 | 0.31 | 0.0994 |
| Rnd1 | -2.75 | 0.0000 |
| Rhebl1 | -0.86 | 0.0009 |
| Lmbr1l | -0.42 | 0.0199 |
| Tuba1b | -0.36 | 0.0074 |
| Tuba1a | -1.64 | 0.0000 |
| Tuba1c | -1.08 | 0.0000 |
| Dnajc22 | 2.96 | 0.0847 |
| Spats2 | 0.49 | 0.0000 |
| Kcnh3 | 2.65 | 0.0000 |
| Fmnl3 | 0.68 | 0.0000 |
| Bcdin3d | -0.44 | 0.0728 |
| Asic1 | 0.35 | 0.0395 |
| Cers5 | 0.28 | 0.0930 |
| Lima1 | 0.52 | 0.0002 |
| Mettl7a1 | 2.80 | 0.0000 |
| Mettl7a3 | 2.81 | 0.0001 |
| Mettl7a2 | 2.84 | 0.0007 |
| Slc11a2 | -1.09 | 0.0000 |
| Csrnp2 | 0.41 | 0.0232 |
| Tfcp2 | 0.67 | 0.0000 |
| Pou6f1 | 0.84 | 0.0000 |
| Slc4a8 | 0.68 | 0.0000 |
| Ankrd33 | 1.27 | 0.0723 |
| Acvr1b | 0.25 | 0.0756 |
| Grasp | 0.25 | 0.0369 |
| Nr4a1 | -1.06 | 0.0000 |
| Soat2 | -2.17 | 0.0000 |
| Csad | 0.56 | 0.0000 |
| Rarg | 0.66 | 0.0000 |
| Mfsd5 | -0.43 | 0.0002 |
| Espl1 | 0.39 | 0.0099 |
| Amhr2 | -1.16 | 0.0465 |
| Pcbp2 | -0.32 | 0.0592 |
| Npff | -0.81 | 0.0833 |
| Hnrnpa1 | -0.62 | 0.0000 |
| Copz1 | 0.43 | 0.0011 |
| Gpr84 | -1.71 | 0.0639 |
| Zfp385a | 0.61 | 0.0101 |
| Itga5 | -0.22 | 0.0903 |
| Nckap1l | -0.93 | 0.0502 |
| Zfp174 | 1.38 | 0.0000 |
| Naa60 | 0.28 | 0.0153 |
| Trap1 | -0.24 | 0.0341 |
| Adcy9 | 1.78 | 0.0000 |
| Glis2 | 0.61 | 0.0000 |
| Coro7 | 0.33 | 0.0349 |
| Vasn | 1.22 | 0.0068 |
| Dnaja3 | -0.41 | 0.0020 |
| Nmral1 | 0.69 | 0.0018 |
| Anks3 | -0.36 | 0.0573 |
| Rogdi | 1.25 | 0.0000 |
| Sec14l5 | 2.00 | 0.0000 |
| Carhsp1 | -0.33 | 0.0081 |
| Usp7 | -0.23 | 0.0608 |
| 1810013L24Rik | -0.52 | 0.0000 |
| Dexi | 0.44 | 0.0514 |
| Clec16a | 0.62 | 0.0001 |
| Rmi2 | 1.04 | 0.0180 |
| Litaf | -0.73 | 0.0000 |
| Snn | 0.81 | 0.0000 |
| Zc3h7a | -0.68 | 0.0003 |
| Rsl1d1 | -0.81 | 0.0000 |
| Snx29 | 1.54 | 0.0000 |
| Cpped1 | 0.55 | 0.0000 |
| Mkl2 | 0.43 | 0.0215 |
| Parn | 0.49 | 0.0005 |
| 3110001I22Rik | -0.52 | 0.0493 |
| Rrn3 | -0.28 | 0.0417 |
| Marf1 | 0.29 | 0.0323 |
| Snai2 | 0.96 | 0.0014 |
| Ube2v2 | -0.35 | 0.0350 |
| Mcm4 | 0.36 | 0.0463 |
| Prkdc | 1.21 | 0.0000 |
| Pkp2 | -0.36 | 0.0272 |
| Yars2 | -0.57 | 0.0000 |
| Fgd4 | 0.46 | 0.0319 |
| Vpreb1 | 1.60 | 0.0630 |
| Sdf2l1 | 0.72 | 0.0001 |
| Med15 | -0.29 | 0.0163 |
| Scarf2 | 0.65 | 0.0007 |
| Dgcr14 | -0.21 | 0.0989 |
| Slc25a1 | -1.71 | 0.0000 |
| Prodh | -0.90 | 0.0000 |
| Zdhhc8 | -0.24 | 0.0344 |
| Ranbp1 | -0.72 | 0.0001 |
| Trmt2a | -0.35 | 0.0584 |
| Arvcf | 0.55 | 0.0542 |
| Gnb1l | 0.89 | 0.0000 |
| Sept5 | -1.06 | 0.0001 |
| Klhl6 | 1.92 | 0.0004 |
| Klhl24 | 0.51 | 0.0061 |
| Parl | -0.42 | 0.0022 |
| Abcc5 | -0.48 | 0.0005 |
| Abcf3 | -0.23 | 0.0876 |
| Vwa5b2 | -1.10 | 0.0378 |
| Camk2n2 | -0.58 | 0.0040 |
| Fam131a | 0.93 | 0.0000 |
| Polr2h | -0.56 | 0.0284 |
| Ephb3 | 1.15 | 0.0039 |
| 2510009E07Rik | 1.00 | 0.0000 |
| Tmem41a | 0.55 | 0.0035 |
| Senp2 | -0.51 | 0.0000 |
| Igf2bp2 | -0.77 | 0.0000 |
| Tra2b | -0.73 | 0.0000 |
| Etv5 | -0.48 | 0.0782 |
| Eif4a2 | -0.39 | 0.0062 |
| Masp1 | 1.31 | 0.0120 |
| Rtp4 | -0.30 | 0.0465 |
| Bcl6 | -0.37 | 0.0813 |
| Leprel1 | 0.46 | 0.0608 |
| Cldn1 | -4.66 | 0.0000 |
| Il1rap | -1.35 | 0.0000 |
| Ccdc50 | -0.31 | 0.0845 |
| Gp5 | -2.38 | 0.0000 |
| Atp13a3 | -1.51 | 0.0000 |
| Lsg1 | -0.71 | 0.0000 |
| Fam43a | 0.33 | 0.0034 |
| Xxylt1 | 0.44 | 0.0085 |
| Ppp1r2 | -0.80 | 0.0000 |
| Senp5 | -0.42 | 0.0481 |
| Cep19 | 0.96 | 0.0000 |
| Tfrc | -1.02 | 0.0000 |
| Tnk2 | -0.30 | 0.0269 |
| 1700021K19Rik | -0.40 | 0.0022 |
| Fyttd1 | -0.32 | 0.0046 |
| Iqcg | 0.35 | 0.0078 |
| Lmln | 1.30 | 0.0000 |
| Snx4 | -0.40 | 0.0007 |
| Slc12a8 | 1.13 | 0.0284 |
| Heg1 | 0.89 | 0.0000 |
| Umps | -0.36 | 0.0089 |
| Mylk | 0.76 | 0.0000 |
| Hspbap1 | -0.95 | 0.0004 |
| Parp14 | -1.00 | 0.0012 |
| Wdr5b | 0.48 | 0.0363 |
| Fam162a | -0.82 | 0.0001 |
| Ccdc58 | -0.45 | 0.0774 |
| Gm5483 | -2.91 | 0.0198 |
| Gm5416 | -2.96 | 0.0000 |
| Stfa2 | -3.35 | 0.0000 |
| Stfa3 | -2.97 | 0.0000 |
| Eaf2 | -1.07 | 0.0294 |
| Iqcb1 | -0.63 | 0.0053 |
| Hcls1 | -0.45 | 0.0000 |
| Hgd | -2.27 | 0.0121 |
| Lrrc58 | -0.27 | 0.0305 |
| Maats1 | -1.30 | 0.0457 |
| Pla1a | -2.68 | 0.0003 |
| Tmem39a | -0.66 | 0.0000 |
| Arhgap31 | 0.47 | 0.0542 |
| B4galt4 | 0.45 | 0.0195 |
| Qtrtd1 | -0.43 | 0.0051 |
| 2610015P09Rik | 0.30 | 0.0935 |
| Zdhhc23 | 1.95 | 0.0005 |
| Naa50 | -0.36 | 0.0040 |
| Spice1 | 0.47 | 0.0124 |
| Cfap44 | 0.46 | 0.0107 |
| Boc | 0.78 | 0.0000 |
| Slc35a5 | 0.37 | 0.0298 |
| Btla | 2.15 | 0.0015 |
| Cd200 | 0.61 | 0.0000 |
| Tmprss7 | -1.99 | 0.0840 |
| Tagln3 | -1.40 | 0.0992 |
| Abhd10 | -0.32 | 0.0608 |
| Phldb2 | -0.82 | 0.0000 |
| Plcxd2 | -0.89 | 0.0599 |
| Gm4737 | -0.34 | 0.0305 |
| C330027C09Rik | 0.34 | 0.0234 |
| Cd47 | -0.49 | 0.0021 |
| Bbx | 0.77 | 0.0000 |
| Cblb | -0.35 | 0.0276 |
| Alcam | -0.42 | 0.0026 |
| Nfkbiz | -2.68 | 0.0000 |
| Nxpe3 | 0.71 | 0.0008 |
| Cep97 | 0.84 | 0.0000 |
| Rpl24 | -0.96 | 0.0678 |
| Pcnp | -0.36 | 0.0512 |
| Trmt10c | -0.41 | 0.0150 |
| Senp7 | 0.80 | 0.0000 |
| Abi3bp | -1.41 | 0.0007 |
| Tfg | -0.29 | 0.0136 |
| Tomm70a | -0.34 | 0.0076 |
| Nit2 | 0.48 | 0.0020 |
| Cmss1 | -0.38 | 0.0827 |
| Filip1l | -0.29 | 0.0353 |
| Col8a1 | 0.97 | 0.0001 |
| Dcbld2 | -1.21 | 0.0000 |
| Mina | -0.55 | 0.0134 |
| Crybg3 | 0.80 | 0.0028 |
| Arl6 | 0.64 | 0.0085 |
| Nsun3 | 0.60 | 0.0020 |
| Pros1 | 0.84 | 0.0000 |
| Zfp654 | 0.30 | 0.0803 |
| Btg3 | -0.64 | 0.0410 |
| Jam2 | 0.73 | 0.0000 |
| Cyyr1 | 0.97 | 0.0000 |
| Adamts1 | -0.90 | 0.0007 |
| Adamts5 | 1.35 | 0.0000 |
| N6amt1 | -0.37 | 0.0053 |
| Usp16 | -0.38 | 0.0181 |
| Cct8 | -0.53 | 0.0000 |
| Bach1 | -0.41 | 0.0548 |
| Scaf4 | -0.49 | 0.0017 |
| Urb1 | -1.16 | 0.0000 |
| Eva1c | -0.58 | 0.0002 |
| Paxbp1 | -0.48 | 0.0118 |
| Ifnar1 | -0.24 | 0.0212 |
| Ifngr2 | -0.51 | 0.0005 |
| Tmem50b | 0.36 | 0.0166 |
| Dnajc28 | 1.96 | 0.0000 |
| Rcan1 | -1.38 | 0.0000 |
| Runx1 | -2.00 | 0.0000 |
| Cbr1 | 0.80 | 0.0003 |
| Morc3 | -0.39 | 0.0176 |
| Ripply3 | 2.55 | 0.0015 |
| Pigp | 0.55 | 0.0332 |
| Ttc3 | 0.29 | 0.0586 |
| Dscr3 | 0.39 | 0.0071 |
| Ets2 | 0.78 | 0.0000 |
| Psmg1 | 0.28 | 0.0725 |
| Wrb | 0.48 | 0.0008 |
| Bace2 | 0.76 | 0.0000 |
| Tmprss2 | -1.40 | 0.0789 |
| C2cd2 | 0.45 | 0.0071 |
| Zbtb21 | -1.12 | 0.0000 |
| Arid1b | 0.47 | 0.0079 |
| Zdhhc14 | 1.14 | 0.0000 |
| Dynlt1c | 0.49 | 0.0086 |
| Ezr | -0.64 | 0.0000 |
| Tagap1 | -0.49 | 0.0269 |
| Fndc1 | 1.48 | 0.0030 |
| Fgfr1op | -0.98 | 0.0000 |
| Mpc1 | -0.59 | 0.0476 |
| Sft2d1 | -0.77 | 0.0017 |
| Agpat4 | -1.02 | 0.0000 |
| Igf2r | 0.86 | 0.0006 |
| Tcp1 | -0.47 | 0.0000 |
| Acat2 | -3.52 | 0.0000 |
| Sod2 | -0.28 | 0.0495 |
| Thbs2 | -0.56 | 0.0384 |
| Phf10 | -0.89 | 0.0000 |
| Rgmb | 0.32 | 0.0052 |
| Zfp51 | -0.43 | 0.0170 |
| Zfp52 | -0.59 | 0.0001 |
| Zfp948 | -1.35 | 0.0000 |
| 3110052M02Rik | -0.69 | 0.0002 |
| Zfp760 | -0.31 | 0.0549 |
| Zfp820 | 0.46 | 0.0208 |
| Zfp942 | -0.63 | 0.0003 |
| Zfp13 | 0.48 | 0.0186 |
| Ccdc64b | -1.93 | 0.0064 |
| Tnfrsf12a | -1.14 | 0.0000 |
| Pkmyt1 | -0.57 | 0.0087 |
| Paqr4 | 1.34 | 0.0000 |
| Prss22 | -2.16 | 0.0001 |
| Prss27 | -2.47 | 0.0001 |
| Kctd5 | -0.33 | 0.0422 |
| Amdhd2 | 0.44 | 0.0356 |
| Atp6v0c | -0.48 | 0.0138 |
| Rnps1 | -0.47 | 0.0015 |
| E4f1 | -0.43 | 0.0415 |
| Pkd1 | 0.57 | 0.0641 |
| Tsc2 | 0.27 | 0.0599 |
| Nthl1 | 0.67 | 0.0093 |
| Zfp598 | -0.64 | 0.0000 |
| Tbl3 | -0.72 | 0.0000 |
| Msrb1 | 0.54 | 0.0001 |
| Fahd1 | 0.75 | 0.0000 |
| Eme2 | -0.61 | 0.0189 |
| Mapk8ip3 | -0.54 | 0.0071 |
| Ift140 | 0.89 | 0.0000 |
| Tmem204 | 2.19 | 0.0000 |
| BC003965 | -0.50 | 0.0046 |
| Tsr3 | -0.59 | 0.0206 |
| Chtf18 | 0.40 | 0.0191 |
| Mslnl | -1.79 | 0.0623 |
| Msln | -1.38 | 0.0042 |
| Haghl | 0.85 | 0.0093 |
| Metrn | 1.52 | 0.0714 |
| Jmjd8 | 0.95 | 0.0000 |
| Wdr90 | 1.06 | 0.0000 |
| Wfikkn1 | -0.77 | 0.0625 |
| Pigq | 0.37 | 0.0022 |
| Decr2 | 0.48 | 0.0036 |
| Axin1 | -0.40 | 0.0056 |
| Luc7l | -0.48 | 0.0026 |
| Neurl1b | 1.36 | 0.0000 |
| Dusp1 | -1.53 | 0.0000 |
| Ergic1 | -0.46 | 0.0000 |
| Crebrf | 0.65 | 0.0000 |
| Syngap1 | 0.64 | 0.0022 |
| Itpr3 | 0.33 | 0.0766 |
| Ip6k3 | 2.24 | 0.0000 |
| Lemd2 | -0.63 | 0.0000 |
| AI413582 | -0.67 | 0.0640 |
| Rps10 | -1.01 | 0.0000 |
| Uhrf1bp1 | -0.37 | 0.0356 |
| Def6 | 0.74 | 0.0000 |
| Ppard | -0.53 | 0.0000 |
| Srpk1 | -0.55 | 0.0000 |
| Mapk14 | 0.72 | 0.0005 |
| Srsf3 | -0.55 | 0.0001 |
| Rab44 | 1.62 | 0.0000 |
| Mtch1 | 0.36 | 0.0014 |
| Pim1 | -0.85 | 0.0010 |
| Tbc1d22b | 0.41 | 0.0052 |
| Rnf8 | -0.63 | 0.0234 |
| Cmtr1 | 0.36 | 0.0464 |
| Mdga1 | 0.61 | 0.0008 |
| Abcg1 | 2.59 | 0.0000 |
| Pde9a | 1.52 | 0.0000 |
| Cbs | 2.37 | 0.0000 |
| U2af1 | -0.46 | 0.0062 |
| Sik1 | -1.19 | 0.0000 |
| Wiz | -0.25 | 0.0468 |
| Cyp4f40 | 1.38 | 0.0000 |
| Zfp799 | 0.62 | 0.0024 |
| Zfp870 | 0.38 | 0.0513 |
| Zfp952 | 0.60 | 0.0004 |
| Zfp763 | 1.43 | 0.0000 |
| Zfp563 | 0.88 | 0.0000 |
| Zfp101 | 0.47 | 0.0115 |
| Angptl4 | -1.75 | 0.0000 |
| Kank3 | -0.46 | 0.0071 |
| Cd320 | 0.77 | 0.0000 |
| Kifc1 | 0.31 | 0.0244 |
| Tapbp | -1.13 | 0.0000 |
| Zbtb22 | -0.32 | 0.0040 |
| Wdr46 | -1.15 | 0.0000 |
| B3galt4 | -1.33 | 0.0914 |
| Rps18 | -0.78 | 0.0068 |
| H2-K1 | -3.00 | 0.0000 |
| Col11a2 | -0.64 | 0.0139 |
| Brd2 | -0.49 | 0.0000 |
| Psmb9 | -0.75 | 0.0002 |
| Tap1 | -0.60 | 0.0064 |
| Psmb8 | -0.78 | 0.0000 |
| Tap2 | -1.18 | 0.0000 |
| Notch4 | -0.26 | 0.0694 |
| Gpsm3 | -0.71 | 0.0828 |
| Pbx2 | 0.62 | 0.0000 |
| Rnf5 | 0.53 | 0.0051 |
| Egfl8 | -0.78 | 0.0028 |
| Prrt1 | -2.01 | 0.0001 |
| Fkbpl | -0.58 | 0.0232 |
| Tnxb | 0.71 | 0.0562 |
| Stk19 | -0.31 | 0.0784 |
| Cfb | -2.78 | 0.0000 |
| Neu1 | -0.94 | 0.0000 |
| Hspa1b | 0.34 | 0.0640 |
| Lsm2 | -0.42 | 0.0560 |
| Vars | -0.76 | 0.0000 |
| Vwa7 | 0.64 | 0.0172 |
| Clic1 | -0.51 | 0.0015 |
| Ly6g6d | 0.96 | 0.0074 |
| Ly6g5b | -1.13 | 0.0340 |
| H2-D1 | -0.65 | 0.0000 |
| H2-Q1 | -1.10 | 0.0000 |
| H2-Q4 | -1.22 | 0.0000 |
| H2-Q6 | -1.87 | 0.0000 |
| Psors1c2 | -1.12 | 0.0137 |
| Ddr1 | -0.59 | 0.0000 |
| Ier3 | -3.29 | 0.0000 |
| Tubb5 | -0.30 | 0.0059 |
| Mdc1 | 0.38 | 0.0838 |
| Ppp1r18 | -0.40 | 0.0002 |
| Mrps18b | -0.88 | 0.0000 |
| Abcf1 | -0.22 | 0.0905 |
| Gnl1 | -0.27 | 0.0425 |
| H2-T24 | 0.80 | 0.0168 |
| Gm11127 | -1.10 | 0.0331 |
| Trim39 | -0.54 | 0.0037 |
| Trim15 | -0.86 | 0.0370 |
| Ppp1r11 | -0.46 | 0.0227 |
| Ubd | -6.23 | 0.0000 |
| Mut | 0.82 | 0.0011 |
| Cd2ap | 0.42 | 0.0051 |
| Cyp39a1 | 1.53 | 0.0242 |
| Rcan2 | 1.13 | 0.0000 |
| Clic5 | -0.37 | 0.0745 |
| Nfkbie | -1.72 | 0.0000 |
| Slc35b2 | 0.29 | 0.0855 |
| Hsp90ab1 | -0.24 | 0.0371 |
| Tmem63b | 0.39 | 0.0001 |
| Vegfa | -1.42 | 0.0000 |
| Yipf3 | 0.29 | 0.0514 |
| Abcc10 | 0.53 | 0.0050 |
| Zfp318 | 0.29 | 0.0838 |
| Ptk7 | 1.00 | 0.0301 |
| Klc4 | 0.53 | 0.0004 |
| Cul7 | 0.31 | 0.0254 |
| Ppp2r5d | 0.30 | 0.0047 |
| Rpl7l1 | -0.36 | 0.0012 |
| Gltscr1l | 0.42 | 0.0072 |
| Mrps10 | -0.69 | 0.0027 |
| Bysl | -0.53 | 0.0000 |
| Usp49 | 0.55 | 0.0018 |
| Frs3 | 0.60 | 0.0669 |
| Foxp4 | -0.66 | 0.0000 |
| Mocs1 | 0.58 | 0.0000 |
| Plcl2 | -0.44 | 0.0090 |
| Tbc1d5 | 0.49 | 0.0619 |
| Pot1b | 0.80 | 0.0187 |
| Ccdc94 | -0.38 | 0.0330 |
| Shd | -0.83 | 0.0706 |
| Stap2 | -1.09 | 0.0001 |
| Chaf1a | 0.45 | 0.0420 |
| Hdgfrp2 | -0.39 | 0.0999 |
| Tnfaip8l1 | -0.84 | 0.0000 |
| Dpp9 | -0.29 | 0.0245 |
| Fem1a | 0.43 | 0.0072 |
| Safb2 | -0.47 | 0.0183 |
| Safb | -0.31 | 0.0224 |
| Rpl36 | -0.81 | 0.0274 |
| Lonp1 | -1.02 | 0.0000 |
| Catsperd | -2.52 | 0.0000 |
| Vmac | 1.38 | 0.0000 |
| Nrtn | 2.16 | 0.0000 |
| Dus3l | -0.36 | 0.0984 |
| Rfx2 | 0.90 | 0.0000 |
| Alkbh7 | 0.86 | 0.0004 |
| Gtf2f1 | -0.75 | 0.0004 |
| C3 | -4.29 | 0.0000 |
| Trip10 | -1.06 | 0.0000 |
| Nudt12 | 1.10 | 0.0000 |
| Efna5 | -0.52 | 0.0023 |
| Fbxl17 | 0.48 | 0.0836 |
| Man2a1 | 0.96 | 0.0001 |
| Vapa | -0.33 | 0.0036 |
| Rab31 | 0.66 | 0.0000 |
| Ddx11 | 0.80 | 0.0000 |
| Mtcl1 | -0.44 | 0.0968 |
| Ptprm | 0.45 | 0.0420 |
| Lama1 | 1.26 | 0.0672 |
| Emilin2 | -0.95 | 0.0000 |
| Wdr43 | -0.93 | 0.0000 |
| Ypel5 | -0.27 | 0.0255 |
| Ehd3 | 0.41 | 0.0264 |
| Memo1 | -0.36 | 0.0239 |
| Slc30a6 | -0.34 | 0.0726 |
| Birc6 | 0.44 | 0.0774 |
| Ttc27 | -0.67 | 0.0000 |
| Fez2 | -0.26 | 0.0305 |
| Strn | 0.52 | 0.0074 |
| Cebpz | -0.64 | 0.0000 |
| Ndufaf7 | 0.30 | 0.0537 |
| Qpct | 0.90 | 0.0001 |
| Cdc42ep3 | -1.59 | 0.0000 |
| Cyp1b1 | 0.49 | 0.0512 |
| Galm | 0.45 | 0.0087 |
| Srsf7 | -0.79 | 0.0000 |
| Morn2 | 0.82 | 0.0584 |
| Map4k3 | -0.61 | 0.0000 |
| Slc8a1 | 0.55 | 0.0395 |
| Pkdcc | 0.91 | 0.0000 |
| Eml4 | 0.60 | 0.0008 |
| Dync2li1 | 1.11 | 0.0000 |
| Lrpprc | -0.35 | 0.0543 |
| Prepl | 0.76 | 0.0000 |
| Prkce | 0.51 | 0.0348 |
| Epas1 | 0.78 | 0.0000 |
| Msh2 | 1.17 | 0.0000 |
| Msh6 | 0.44 | 0.0151 |
| Fbxo11 | -0.43 | 0.0005 |
| Foxn2 | -1.08 | 0.0000 |
| Ston1 | 0.92 | 0.0161 |
| Gm20939 | -1.37 | 0.0000 |
| Kdm5d | 0.35 | 0.0967 |
| Erdr1 | -1.71 | 0.0063 |
| Crem | -0.25 | 0.0437 |
| Bambi | 0.84 | 0.0020 |
| Map3k8 | -1.41 | 0.0000 |
| Mtpap | -0.32 | 0.0483 |
| Zeb1 | -0.49 | 0.0008 |
| Arhgap12 | -0.35 | 0.0270 |
| Mpp7 | 0.64 | 0.0015 |
| Fzd8 | 0.37 | 0.0277 |
| Thoc1 | -1.01 | 0.0000 |
| Usp14 | -0.28 | 0.0865 |
| Snrpd1 | -0.55 | 0.0356 |
| Cables1 | 0.83 | 0.0003 |
| Tmem241 | 1.12 | 0.0000 |
| Riok3 | -0.62 | 0.0000 |
| 3110002H16Rik | -0.46 | 0.0029 |
| Ankrd29 | 1.42 | 0.0000 |
| Impact | -0.82 | 0.0000 |
| Zfp521 | 1.34 | 0.0000 |
| Taf4b | -0.90 | 0.0000 |
| Kctd1 | 1.09 | 0.0000 |
| B4galt6 | 0.55 | 0.0006 |
| Rnf125 | 1.52 | 0.0000 |
| Rnf138 | -0.36 | 0.0256 |
| Nol4 | 0.84 | 0.0009 |
| Mapre2 | 1.05 | 0.0000 |
| Zfp35 | -0.58 | 0.0001 |
| Ino80c | -0.81 | 0.0000 |
| Slc39a6 | -0.91 | 0.0000 |
| Tpgs2 | 0.57 | 0.0021 |
| AW554918 | 0.39 | 0.0489 |
| Celf4 | 1.34 | 0.0008 |
| Gpr17 | 0.94 | 0.0246 |
| Map3k2 | 0.52 | 0.0614 |
| Bin1 | -1.02 | 0.0000 |
| Tslp | -1.65 | 0.0000 |
| Wdr36 | -0.76 | 0.0000 |
| Camk4 | 0.51 | 0.0338 |
| Stard4 | -1.47 | 0.0000 |
| Epb4.1l4a | 0.49 | 0.0047 |
| Srp19 | -0.34 | 0.0530 |
| Cdc23 | 0.57 | 0.0002 |
| Egr1 | -1.67 | 0.0000 |
| Etf1 | -0.45 | 0.0661 |
| Hspa9 | -0.93 | 0.0000 |
| Ctnna1 | -0.37 | 0.0009 |
| Lrrtm2 | -1.69 | 0.0149 |
| Matr3 | -0.26 | 0.0335 |
| Paip2 | 0.39 | 0.0069 |
| Dnajc18 | 0.79 | 0.0000 |
| Tmem173 | 0.61 | 0.0001 |
| Ube2d2a | -0.38 | 0.0007 |
| Cxxc5 | 1.28 | 0.0000 |
| Pura | 0.29 | 0.0849 |
| Cystm1 | -1.25 | 0.0001 |
| Hbegf | -2.21 | 0.0000 |
| Cd14 | -2.92 | 0.0000 |
| Tmco6 | -0.82 | 0.0001 |
| Ik | -0.35 | 0.0366 |
| Wdr55 | -1.10 | 0.0000 |
| Dnd1 | -0.84 | 0.0582 |
| Hars | -0.41 | 0.0049 |
| Pcdhb10 | -0.83 | 0.0358 |
| Pcdhb17 | -0.78 | 0.0001 |
| Pcdhb18 | -0.64 | 0.0032 |
| Pcdhb20 | -0.71 | 0.0012 |
| Pcdhb22 | -0.89 | 0.0000 |
| Taf7 | -0.38 | 0.0712 |
| Pcdhga5 | -1.51 | 0.0164 |
| PCDHGA12 | -1.45 | 0.0034 |
| PCDHGB6 | -0.50 | 0.0967 |
| Pcdhgc4 | 0.78 | 0.0016 |
| Arap3 | -0.38 | 0.0031 |
| Pcdh1 | -0.45 | 0.0311 |
| 0610009O20Rik | 0.43 | 0.0008 |
| Pcdh12 | 1.01 | 0.0000 |
| Spry4 | -1.10 | 0.0000 |
| Fgf1 | 1.00 | 0.0724 |
| Arhgap26 | -1.20 | 0.0000 |
| Yipf5 | 0.49 | 0.0015 |
| Lars | -0.94 | 0.0000 |
| Tcerg1 | -0.45 | 0.0007 |
| Eif3j2 | -0.43 | 0.0085 |
| Eif1a | -1.05 | 0.0000 |
| Atg12 | 0.37 | 0.0281 |
| Sema6a | 0.73 | 0.0000 |
| Srfbp1 | -0.54 | 0.0082 |
| Csnk1g3 | -0.37 | 0.0161 |
| Zfp608 | 1.02 | 0.0000 |
| Gramd3 | 0.39 | 0.0602 |
| Aldh7a1 | 0.27 | 0.0990 |
| Phax | -0.34 | 0.0890 |
| Lmnb1 | 0.38 | 0.0607 |
| March3 | 0.92 | 0.0966 |
| Megf10 | 3.76 | 0.0000 |
| Slc12a2 | -1.16 | 0.0000 |
| Isoc1 | 0.91 | 0.0015 |
| Smim3 | -0.55 | 0.0285 |
| Myoz3 | -1.31 | 0.0001 |
| Cd74 | -2.71 | 0.0138 |
| Tcof1 | -0.67 | 0.0006 |
| Ppargc1b | 1.63 | 0.0008 |
| Grpel2 | -1.25 | 0.0000 |
| Afap1l1 | 0.27 | 0.0339 |
| Adrb2 | 0.75 | 0.0489 |
| Piezo2 | 1.93 | 0.0000 |
| Txnl1 | -0.35 | 0.0062 |
| Wdr7 | 1.00 | 0.0001 |
| Fech | 0.53 | 0.0003 |
| Nars | -1.21 | 0.0000 |
| Atp8b1 | -1.03 | 0.0000 |
| Nedd4l | -0.77 | 0.0000 |
| Sec11c | -0.49 | 0.0627 |
| Ccbe1 | -2.85 | 0.0000 |
| Mppe1 | 1.38 | 0.0000 |
| Impa2 | 1.05 | 0.0021 |
| Tubb6 | -1.05 | 0.0000 |
| Afg3l2 | -0.33 | 0.0329 |
| Spire1 | -0.42 | 0.0964 |
| Ptpn2 | -0.60 | 0.0000 |
| Ldlrad4 | 1.97 | 0.0000 |
| Rnmt | -0.33 | 0.0549 |
| Tcf4 | -0.71 | 0.0000 |
| 4930503L19Rik | -0.71 | 0.0000 |
| Mbd2 | -0.58 | 0.0000 |
| Mex3c | -0.64 | 0.0000 |
| Acaa2 | 0.91 | 0.0000 |
| Lipg | -6.90 | 0.0000 |
| Rpl17 | -0.76 | 0.0068 |
| Zbtb7c | -0.53 | 0.0039 |
| Hdhd2 | 0.53 | 0.0255 |
| Pias2 | 0.31 | 0.0850 |
| 8030462N17Rik | -0.41 | 0.0097 |
| Haus1 | 0.69 | 0.0259 |
| Pstpip2 | -0.78 | 0.0743 |
| Ctdp1 | -0.64 | 0.0000 |
| Nfatc1 | 0.86 | 0.0001 |
| Tshz1 | -0.48 | 0.0003 |
| Rttn | 0.64 | 0.0018 |
| Cpt1a | 0.89 | 0.0000 |
| Lrp5 | 1.01 | 0.0000 |
| Chka | -0.87 | 0.0000 |
| Aldh3b1 | 0.85 | 0.0000 |
| Unc93b1 | 0.25 | 0.0665 |
| Acy3 | 1.76 | 0.0000 |
| Gstp1 | 0.53 | 0.0000 |
| Cabp4 | -1.06 | 0.0101 |
| Gpr152 | -2.26 | 0.0008 |
| Coro1b | -0.22 | 0.0787 |
| Carns1 | 0.33 | 0.0640 |
| Tbc1d10c | 1.24 | 0.0029 |
| Rad9a | -0.36 | 0.0115 |
| Clcf1 | -0.91 | 0.0000 |
| Ankrd13d | 1.03 | 0.0000 |
| Rhod | 0.40 | 0.0422 |
| Syt12 | 0.61 | 0.0560 |
| Pcx | 0.67 | 0.0004 |
| Rce1 | -0.35 | 0.0103 |
| Sptbn2 | -0.42 | 0.0042 |
| Rbm4 | -0.53 | 0.0297 |
| Ctsf | 0.78 | 0.0000 |
| Bbs1 | 1.56 | 0.0000 |
| Dpp3 | -0.23 | 0.0656 |
| Rin1 | 0.24 | 0.0617 |
| Rab1b | 0.46 | 0.0004 |
| Pacs1 | 0.63 | 0.0002 |
| Eif1ad | -0.52 | 0.0001 |
| Sart1 | -0.58 | 0.0025 |
| Drap1 | -0.45 | 0.0418 |
| AI837181 | -0.70 | 0.0000 |
| Fosl1 | -2.14 | 0.0000 |
| Efemp2 | -1.70 | 0.0000 |
| Mus81 | -0.38 | 0.0751 |
| Kat5 | -0.46 | 0.0019 |
| Rela | -0.99 | 0.0000 |
| Ehbp1l1 | -0.53 | 0.0000 |
| Sssca1 | -1.70 | 0.0039 |
| Ltbp3 | 0.40 | 0.0039 |
| Scyl1 | -0.37 | 0.0237 |
| Mrpl49 | 0.32 | 0.0151 |
| Tm7sf2 | -2.23 | 0.0000 |
| Cdca5 | 0.28 | 0.0608 |
| Ppp2r5b | -0.53 | 0.0019 |
| Ehd1 | -0.77 | 0.0000 |
| Map4k2 | 0.81 | 0.0000 |
| Sf1 | -0.48 | 0.0000 |
| Rps6ka4 | -0.56 | 0.0000 |
| Prdx5 | -0.79 | 0.0000 |
| Ppp1r14b | -0.98 | 0.0003 |
| Nudt22 | 0.59 | 0.0360 |
| Trpt1 | 0.72 | 0.0071 |
| Stip1 | -0.76 | 0.0000 |
| Macrod1 | -0.48 | 0.0051 |
| Rcor2 | 1.94 | 0.0000 |
| Mark2 | -0.47 | 0.0005 |
| AI846148 | -0.42 | 0.0918 |
| 2700081O15Rik | 0.46 | 0.0018 |
| Slc3a2 | -1.79 | 0.0000 |
| Wdr74 | -0.95 | 0.0000 |
| Nxf1 | -0.42 | 0.0353 |
| Gng3 | -0.94 | 0.0213 |
| Rom1 | 0.75 | 0.0000 |
| Tut1 | -0.81 | 0.0000 |
| Asrgl1 | 1.79 | 0.0012 |
| Rab3il1 | 3.10 | 0.0000 |
| Fads3 | -1.30 | 0.0000 |
| Fads2 | -2.39 | 0.0000 |
| Fads1 | -0.40 | 0.0109 |
| Syt7 | 2.04 | 0.0000 |
| Cpsf7 | -0.55 | 0.0042 |
| Cyb561a3 | 0.40 | 0.0064 |
| Dak | 0.58 | 0.0000 |
| Vwce | -1.29 | 0.0375 |
| Vps37c | -0.75 | 0.0000 |
| Tmem132a | 0.32 | 0.0671 |
| Tmem109 | 0.34 | 0.0066 |
| Prpf19 | -0.24 | 0.0229 |
| Ccdc86 | -0.51 | 0.0014 |
| Ms4a4d | -2.44 | 0.0058 |
| Ms4a6d | -0.30 | 0.0097 |
| Stx3 | -0.37 | 0.0168 |
| Olfr1420 | -1.26 | 0.0089 |
| Patl1 | -0.46 | 0.0017 |
| Dtx4 | 2.68 | 0.0000 |
| Glyat | 1.29 | 0.0787 |
| Psat1 | -0.53 | 0.0061 |
| Cep78 | 0.64 | 0.0007 |
| Gnaq | 0.63 | 0.0000 |
| Rfk | 0.59 | 0.0002 |
| Pcsk5 | 0.92 | 0.0000 |
| Nmrk1 | 0.59 | 0.0041 |
| Trpm6 | 1.57 | 0.0428 |
| Rorb | -1.87 | 0.0000 |
| Anxa1 | -0.53 | 0.0000 |
| Zfand5 | -0.85 | 0.0000 |
| Gda | 1.71 | 0.0000 |
| Trpm3 | 2.16 | 0.0008 |
| Fam189a2 | 1.53 | 0.0000 |
| Tjp2 | -0.63 | 0.0002 |
| Fxn | -0.39 | 0.0872 |
| Pip5k1b | 1.29 | 0.0308 |
| Pgm5 | 1.78 | 0.0000 |
| Dock8 | 0.63 | 0.0131 |
| Kank1 | 1.09 | 0.0000 |
| Smarca2 | 1.42 | 0.0000 |
| Vldlr | -0.51 | 0.0395 |
| D19Bwg1357e | -0.95 | 0.0000 |
| Ak3 | 0.88 | 0.0000 |
| Rcl1 | -0.72 | 0.0000 |
| Cd274 | -0.73 | 0.0051 |
| Ermp1 | 0.53 | 0.0170 |
| Ranbp6 | 0.62 | 0.0000 |
| Asah2 | 0.61 | 0.0004 |
| Papss2 | -1.02 | 0.0314 |
| Atad1 | -0.41 | 0.0023 |
| Pten | 0.28 | 0.0271 |
| Ch25h | -1.25 | 0.0000 |
| Ifit2 | -0.83 | 0.0166 |
| Pcgf5 | -0.50 | 0.0061 |
| Ppp1r3c | 2.23 | 0.0000 |
| Ide | 0.37 | 0.0308 |
| Kif11 | 0.49 | 0.0809 |
| Hhex | 1.06 | 0.0000 |
| Myof | -0.85 | 0.0000 |
| Noc3l | -0.85 | 0.0000 |
| Tbc1d12 | -0.40 | 0.0250 |
| Pdlim1 | -0.51 | 0.0000 |
| Sorbs1 | 1.48 | 0.0000 |
| Aldh18a1 | -1.04 | 0.0000 |
| Tctn3 | 1.20 | 0.0000 |
| Entpd1 | 2.08 | 0.0000 |
| Tll2 | -2.76 | 0.0000 |
| Arhgap19 | 0.74 | 0.0009 |
| Frat2 | -0.57 | 0.0364 |
| Rrp12 | -1.07 | 0.0000 |
| Exosc1 | -0.58 | 0.0010 |
| Ubtd1 | -0.67 | 0.0002 |
| Ankrd2 | -1.02 | 0.0075 |
| 4933411K16Rik | 1.82 | 0.0149 |
| Morn4 | 1.01 | 0.0000 |
| Pi4k2a | -0.32 | 0.0061 |
| Avpi1 | -0.45 | 0.0242 |
| Marveld1 | 1.01 | 0.0000 |
| Sfrp5 | -1.09 | 0.0562 |
| Pyroxd2 | 0.69 | 0.0016 |
| Got1 | -0.56 | 0.0003 |
| Nkx2-3 | 1.56 | 0.0007 |
| Slc25a28 | -0.41 | 0.0304 |
| Entpd7 | 0.60 | 0.0009 |
| Dnmbp | 0.38 | 0.0500 |
| Chuk | -0.32 | 0.0382 |
| Pkd2l1 | 1.51 | 0.0090 |
| Scd3 | 2.13 | 0.0000 |
| Scd1 | -0.76 | 0.0012 |
| Pax2 | 1.18 | 0.0000 |
| Sema4g | 0.78 | 0.0239 |
| Peo1 | -0.26 | 0.0576 |
| Poll | 0.60 | 0.0002 |
| Dpcd | 0.65 | 0.0007 |
| Npm3 | -0.86 | 0.0044 |
| Hps6 | -0.40 | 0.0007 |
| Pprc1 | -0.98 | 0.0000 |
| Nolc1 | -0.94 | 0.0000 |
| Gbf1 | 0.67 | 0.0009 |
| Psd | -2.23 | 0.0000 |
| Sufu | 0.63 | 0.0179 |
| Sfxn2 | 1.24 | 0.0009 |
| Cnnm2 | 1.58 | 0.0000 |
| Nt5c2 | -0.30 | 0.0201 |
| Pcgf6 | -0.54 | 0.0009 |
| Pdcd11 | -0.60 | 0.0000 |
| Calhm2 | 1.03 | 0.0000 |
| Neurl1a | -1.48 | 0.0149 |
| Sfr1 | -0.38 | 0.0152 |
| Add3 | 0.90 | 0.0003 |
| Mxi1 | 0.78 | 0.0000 |
| Smndc1 | -0.49 | 0.0000 |
| Dusp5 | -1.83 | 0.0000 |
| Rbm20 | 0.75 | 0.0398 |
| Gpam | 1.09 | 0.0000 |
| Acsl5 | -0.21 | 0.0804 |
| Vti1a | 0.44 | 0.0096 |
| Tcf7l2 | 0.64 | 0.0008 |
| Casp7 | 0.23 | 0.0887 |
| Plekhs1 | 0.40 | 0.0984 |
| Nhlrc2 | -0.47 | 0.0113 |
| Afap1l2 | 0.76 | 0.0000 |
| Ablim1 | 1.55 | 0.0000 |
| Trub1 | -0.48 | 0.0040 |
| Slc18a2 | -0.60 | 0.0777 |
| Nanos1 | 0.73 | 0.0009 |
| Eif3a | -0.52 | 0.0001 |
| Csf2ra | -0.77 | 0.0087 |
| mt-Nd1 | -0.93 | 0.0051 |
| mt-Nd2 | -1.07 | 0.0014 |
| mt-Co1 | -0.87 | 0.0119 |
| mt-Co2 | -0.91 | 0.0081 |
| mt-Atp6 | -0.96 | 0.0050 |
| mt-Co3 | -0.87 | 0.0075 |
| mt-Nd3 | -1.08 | 0.0019 |
| mt-Nd4l | -0.72 | 0.0279 |
| mt-Nd4 | -0.92 | 0.0063 |
| mt-Nd5 | -0.85 | 0.0074 |
| mt-Nd6 | -0.97 | 0.0023 |
| mt-Cytb | -0.94 | 0.0081 |
| Spry3 | 1.31 | 0.0508 |
| Tmlhe | 0.44 | 0.0234 |
| Cd99 | -1.15 | 0.0000 |

**Supplementary Table 5. Differentially expressed genes in chicken chorioallantoic membranes treated with methyl-β-cyclodextrin.**

| **ENSEMBL ID** | **symbol** | **Log2FC** | **padj** |
| --- | --- | --- | --- |
| ENSGALG00010003363 | HBE1 | -1.01 | 0.0370 |
| ENSGALG00010003868 | HBBA | -1.12 | 0.0148 |
| ENSGALG00010010791 | H-10 | -0.85 | 0.0251 |
| ENSGALG00010021710 |  | -1.04 | 0.0790 |
| ENSGALG00010024915 | STXBP5L | -0.86 | 0.0985 |
| ENSGALG00010000959 |  | -1.72 | 0.0271 |
| ENSGALG00010000985 |  | -2.88 | 0.0021 |
| ENSGALG00010004296 |  | 0.71 | 0.0790 |
| ENSGALG00010005413 | GAD2 | -0.93 | 0.0858 |
| ENSGALG00010005534 |  | -0.65 | 0.0121 |
| ENSGALG00010012483 |  | -1.22 | 0.0148 |
| ENSGALG00010024834 | INSIG1 | -0.74 | 0.0720 |
| ENSGALG00010002062 | GSTA4 | -0.91 | 0.0007 |
| ENSGALG00010017562 | SLC30A10 | -1.18 | 0.0105 |
| ENSGALG00010007877 | MSMO1 | -0.66 | 0.0446 |
| ENSGALG00010008751 |  | -1.61 | 0.0098 |
| ENSGALG00010012857 | NSDHL | -0.64 | 0.0501 |
| ENSGALG00010011634 | AVD | 1.40 | 0.0034 |
| ENSGALG00010006291 |  | -1.38 | 0.0105 |
| ENSGALG00010021777 |  | -1.08 | 0.0001 |
| ENSGALG00010015661 |  | -2.86 | 0.0523 |
| ENSGALG00010018361 | ASIC4 | -0.88 | 0.0374 |
| ENSGALG00010022552 | GPR155 | -0.87 | 0.0720 |
| ENSGALG00010020776 | MCOLN3 | -1.27 | 0.0453 |
| ENSGALG00010013759 | ATP13A4 | 0.79 | 0.0976 |
| ENSGALG00010024897 | ADAL | -0.75 | 0.0720 |
| ENSGALG00010028310 |  | -3.66 | 0.0705 |
| ENSGALG00010025325 | DPEP2 | -1.20 | 0.0338 |
| ENSGALG00010019253 | HBAD | -1.05 | 0.0271 |
| ENSGALG00010019446 |  | 2.19 | 0.0956 |
| ENSGALG00010019732 | HBA1 | -1.20 | 0.0079 |
| ENSGALG00010016324 | TUBB1 | -1.05 | 0.0173 |
| ENSGALG00010024286 | ADA | -0.81 | 0.0105 |
| ENSGALG00010024537 | EPB42 | -1.05 | 0.0414 |
| ENSGALG00010029150 | NARF | -0.79 | 0.0252 |
| ENSGALG00010024451 | PHOSPHO1 | -1.20 | 0.0790 |
| ENSGALG00010024885 | SLC4A1 | -0.98 | 0.0522 |
| ENSGALG00010018193 | ANK1 | -1.20 | 0.0720 |
| ENSGALG00010018944 | NEFL | -1.13 | 0.0358 |
| ENSGALG00010024061 |  | 1.68 | 0.0001 |
| ENSGALG00010025927 |  | 2.09 | 0.0790 |
| ENSGALG00010028216 | NKX2-3 | 1.92 | 0.0004 |
| ENSGALG00010028374 | FDPS | -0.60 | 0.0790 |
